# Genotype to phenotype: Diet-by-mitochondrial DNA haplotype interactions drive metabolic flexibility and organismal fitness

**DOI:** 10.1371/journal.pgen.1007735

**Published:** 2018-11-06

**Authors:** Wen C. Aw, Samuel G. Towarnicki, Richard G. Melvin, Neil A. Youngson, Michael R. Garvin, Yifang Hu, Shaun Nielsen, Torsten Thomas, Russell Pickford, Sonia Bustamante, Antón Vila-Sanjurjo, Gordon K. Smyth, J. William O. Ballard

**Affiliations:** 1 School of Biotechnology and Biomolecular Sciences, The University of New South Wales, Sydney, NSW, Australia; 2 School of Biological Sciences, Washington State University, Pullman, Washington, United States of America; 3 The Walter and Eliza Hall Institute of Medical Research, Melbourne, Victoria, Australia; 4 Centre for Marine Bio-Innovation and School of Biological, Earth and Environmental Sciences, The University of New South Wales, Sydney, NSW, Australia; 5 Bioanalytical Mass Spectrometry Facility, Mark Wainwright Analytical Center, The University of New South Wales, Sydney, NSW, Australia; 6 Grupo GIBE, Bioloxía Celular e Molecular, Facultade de Ciencias, Universidade da Coruña (UDC), Campus Zapateira s/n, A Coruña, Spain; 7 School of Mathematics and Statistics, The University of Melbourne, Melbourne, Victoria, Australia; The Francis Crick Institute, UNITED KINGDOM

## Abstract

Diet may be modified seasonally or by biogeographic, demographic or cultural shifts. It can differentially influence mitochondrial bioenergetics, retrograde signalling to the nuclear genome, and anterograde signalling to mitochondria. All these interactions have the potential to alter the frequencies of mtDNA haplotypes (mitotypes) in nature and may impact human health. In a model laboratory system, we fed four diets varying in Protein: Carbohydrate (P:C) ratio (1:2, 1:4, 1:8 and 1:16 P:C) to four homoplasmic *Drosophila melanogaster* mitotypes (nuclear genome standardised) and assayed their frequency in population cages. When fed a high protein 1:2 P:C diet, the frequency of flies harbouring Alstonville mtDNA increased. In contrast, when fed the high carbohydrate 1:16 P:C food the incidence of flies harbouring Dahomey mtDNA increased. This result, driven by differences in larval development, was generalisable to the replacement of the laboratory diet with fruits having high and low P:C ratios, perturbation of the nuclear genome and changes to the microbiome. Structural modelling and cellular assays suggested a V161L mutation in the ND4 subunit of complex I of Dahomey mtDNA was mildly deleterious, reduced mitochondrial functions, increased oxidative stress and resulted in an increase in larval development time on the 1:2 P:C diet. The 1:16 P:C diet triggered a cascade of changes in both mitotypes. In Dahomey larvae, increased feeding fuelled increased β-oxidation and the partial bypass of the complex I mutation. Conversely, Alstonville larvae upregulated genes involved with oxidative phosphorylation, increased glycogen metabolism and they were more physically active. We hypothesise that the increased physical activity diverted energy from growth and cell division and thereby slowed development. These data further question the use of mtDNA as an assumed neutral marker in evolutionary and population genetic studies. Moreover, if humans respond similarly, we posit that individuals with specific mtDNA variations may differentially metabolise carbohydrates, which has implications for a variety of diseases including cardiovascular disease, obesity, and perhaps Parkinson’s Disease.

## Author summary

The detection and quantitation of mtDNA polymorphisms in populations and across whole habitats continues to be used as a central investigatory tool in evolutionary genetics. But, the approach is laden with assumptions about selection that are rarely examined. We present a series of studies that traverse the genotype to the phenotype. The studies were designed to experimentally test the interaction between diet and mitotype in *Drosophila* flies and provide a mechanism by which selection occurs. We start with population cage studies that include four laboratory diets and four mitotypes. We then directly compete two mitotypes (Alstonville and Dahomey) on a high protein and a high carbohydrate diet and show a flip in their relative fitness that is driven by differences in larval development. Next, we identify a single naturally-occurring point mutation, which drives the cage results. We track the ripple effects up to the level of the organelle (mitochondria), through the labyrinth of metabolic pathways and on to the phenotype. Notably, when flies were fed the high carbohydrate diet, energy metabolism was extensively remodelled in both mitotypes causing increased physical activity in Alstonville flies. These data invite an extensive experimental re-evaluation of the assumption that mtDNA inescapably evolves in a manner consistent with a strictly neutral equilibrium model. It also motivates investigation of genotype-specific dietary manipulation as an integrative treatment of human disorders involving mitochondrial metabolism and offers the potential for future therapeutic strategies.

## Introduction

"*Dis-moi ce que tu manges*, *je te dirai ce que tu es*." Anthelme Brillat-Savarin 1826

Diet and an organism’s genes contribute towards its phenotype and impact a range of scientific disciplines that span from the more fundamental disciplines of evolutionary biology and quantitative genetics to the more medically applied fields of nutrigenomics and pharmacogenomics. In nature, the dietary macronutrient balance is a strong selective force within and among populations. The relative proportions of macronutrients in food can fluctuate seasonally, vary when species colonise new habitats and can influence the frequency of alleles in populations [1, 2]. It is well documented that nutritional responses vary with genotype [3–8] and it has been convincingly argued that the human genome is maladapted to our 21^st^ century diet [9]. Dietary modification is an established treatment for certain diseases including cardiovascular disease, diabetes, and obesity [10], yet, we still have an incomplete knowledge of how genetic variants that modulate susceptibility to disease are influenced by exogenous factors. This study explores the potential for diet to differentially influence mitochondrial function and the organismal health and fitness of *Drosophila melanogaster* flies harbouring distinct mtDNA types (mitotypes).

Protein and carbohydrate are the two primary energy-yielding macronutrients in fly food, and their ratio has been shown to have profound impacts on various aspects of physiology, behaviour, and biochemistry [11, 12]. In adult females of *D*. *melanogaster* Canton S, a 1:2 Protein: Carbohydrate (P:C) ratio of food yielded the highest egg-laying rate and a 1:16 P:C ratio maximised survival [13]. Aw et al. [14] and Towarnicki and Ballard [15] demonstrated a more complex scenario whereby diet interacted with *Drosophila* mitotype and with other environmental factors such as temperature. For adults, Aw et al. [14] reported sex-specific influences of mitotype and diet on mitochondrial functions and physiological traits in males harbouring the Alstonville and Japan mitotypes. In larvae, Towarnicki and Ballard [15] manipulated food and temperature to study the development of the Alstonville and Dahomey mitotypes. We observed that larvae harbouring the latter mitotype developed more slowly than the former when fed a high protein diet at all temperatures, but more quickly when fed the high carbohydrate diet at higher temperatures. These studies did not determine the magnitude of selection at an organismal level or differentiate the relative importance of the interactions in larvae and adult stages, nor did they provide a mechanism of action. Toward these goals, we constructed laboratory diets that differed in their P:C ratios (1:2, 1:4, 1:8 and 1:16 P:C) and also tested natural fruits that differed in their P:C ratio.

Laboratory population cage studies are a sensitive method to test for selection in *Drosophila* and the frequency of each genotype type in cages is taken as an indicator of fitness [16, 17]. Previous cage studies have provided evidence that distinct mitotypes can influence the frequency of flies in the laboratory, but none of these studies manipulated the diet [17–19]. The cage study paradigm used here does not involve flies breeding until termination of fecundity or lifespan; instead, it enforces a short window for flies to lay eggs. As a consequence, repeatable changes in the frequency of genotypes are caused by differences in immature development time and the fitness of young adults during the period that larval fat body remains [20]. Other experimental methods that have been utilised to estimate fitness of flies harbouring different mitotypes include *in vivo* competition and assaying mitotype frequencies of wild-caught animals [e.g., 21, 22, 23]. Ma and O’Farrell [21] created fly lines with multiple mitotypes and utilised the uniparental mode of inheritance in mitochondria to test for selection. They observed that non-coding differences in the origin of replication region could cause the frequency of individuals harbouring a genome with a detrimental mutation to increase, but then lead to population death after several generations. The mechanism for this is still unknown. Thermal selection has been proposed to shape the pattern of mtDNA variation in eastern Australian *D*. *melanogaster*, but no experimental information has been provided on which mutation(s) may be driving these data [22, 24]. Here, we chose the population cage paradigm for its high sensitivity and have quantified the frequencies of four globally sourced *D*. *melanogaster* mitotypes (Alstonville, Dahomey, Japan and *w*^1118^) fed our four P:C diets. We then directly compete two mitotypes fed two diets.

Mechanistically, provisioning of dietary macronutrients to mitochondria may be influenced by genetic variations that influence the activity of the electron transport system, organelle retrograde signalling to the nuclear genome, anterograde signalling to the mitochondrion and epigenetic modifications [12, 25]. These variations may result from mtDNA mutations, mito-nuclear interactions and nuclear-encoded differences [7, 22, 26–28]. Mitochondria produce energy by utilising electrons harvested from oxidisable dietary substrates and O_2_ to build up a proton-motive force by pumping protons from the mitochondrial matrix into the intermembrane space. The subsequent backflow of protons to the matrix across complex V (ATP synthase) of the inner membrane drives the synthesis of ATP. Functional differences in mitochondrial energy production influence evolutionarily important physiological and organismal traits. In *Drosophila*, these traits include development time and egg production, and in humans, they include inherited disease and the decline in mitochondrial function with advancing age [25, 29–31]. Here, we identified functionally significant differences between mitotypes by carefully controlling the nuclear genetic background, modelling quaternary and secondary structures, conducting multiple independent *in vitro* assays, adding electron transport system inhibitors to the diets, and assaying independently collected mitotypes [32–35].

Is it possible that a given mtDNA mutation could be slightly deleterious in one environment but advantageous in another? If a mtDNA mutation is functionally deleterious, and linked mutations are neutral or nearly neutral, current models predict that the mitotype will have a selective disadvantage, causing it to decline in frequency in nature and population cage studies. Slightly deleterious mutations have been reported in *Drosophila* [26, 36–38], purifying selection has been demonstrated in the mouse female germline [39, 40], and deleterious mtDNA mutations are well-known in humans [41–43]. However, as dietary stress increases, genotype-specific mitochondrial responses may trigger flexible and broad cytosolic and nuclear reactions that have collectively been termed mitohormesis [44]. Remarkably, rather than being harmful, these changes caused by low levels of stress can result in a reconfiguration of metabolism, which in turn can enable increased production of ATP, increased evolutionary potential, and decreased susceptibility to disease [12, 45]. Again, the mechanisms for this are not well understood. Various mechanisms by which stressed mitochondria may signal outward to the cytosol and the nucleus have been proposed. These include regulation of ATP levels, altering mitochondrial membrane potential to allow recruitment and assembly of signalling molecules, and the production of reactive oxygen species (ROS). These are, however, not the only available pathways in the mitochondrial repertoire [44]. For instance, calcium signalling from the endoplasmic reticulum likely influences a multitude of mitochondrial functions [46, 47]. Above a genotype-specific threshold, increasing the level of a specific stressor is expected to be deleterious and disease-causing, with the distribution of the response determined by the “norm of reaction” [48]. The norm of reaction describes the pattern of phenotypic expression across a range of environments and may be entirely different for two mitotypes. To investigate the possibility of functional compensation through mitohormesis, here, we conducted transcriptomics and metabolomics studies. We then experientially examined the mechanisms involved by manipulating dietary sugars and inhibiting specific metabolic pathways.

Our series of studies show that a diet by mitotype interaction mediated the remodelling of carbohydrate metabolism in two *Drosophila* mitotypes. When fed the high protein 1:2 P:C diet, the slightly deleterious ND4 mutation in complex I of Dahomey mtDNA caused the mitotype to have a selective *disadvantage* compared to those harbouring Alstonville mtDNA. Complex I is the primary entry point for electrons into the mitochondrial electron transport system and is a site of electron leak to oxygen and the generation of ROS [49]. In contrast, when fed the high carbohydrate 1:16 P:C diet, mitotypes differentially remodelled energy metabolism, and this resulted in an evolutionary *advantage* to Dahomey. Were the same mechanisms found to occur in humans, the enhanced lipogenesis in individuals with slightly deleterious complex I mutations could make them more susceptible to obesity when eating a high carbohydrate diet, however, for those individuals with a predisposition to Parkinson’s disease, which has been linked to defects in lipogenesis [50], this diet could delay onset or rate of decline.

## Results and discussion

Unravelling the influence of diet on DNA variations is a challenge with broad evolutionary, health care and disease implications [12, 51], yet we still have an incomplete knowledge of the mechanisms involved. In this series of studies, we tested the interaction between diet and mitotype in *Drosophila* to determine the presence and mechanism of selection. We tracked mitotype specific effects up to the level of the mitochondrion, through the morass of metabolic pathways and on to the phenotype. We concluded that differential provisioning of macronutrients to mitochondria harbouring distinct mitotypes led to phenotypic changes in food consumption, starvation resistance, and movement, as oxidative phosphorylation and β-oxidation of fatty acids were differentially regulated.

### Study 1: Population cages, larval development and adult fitness of four mitotypes

To test the hypothesis that the fitness of mitotypes can be differentially influenced by diet [12], we fed four diets varying in P:C ratio to four *Drosophila* mitotypes and assayed their frequency in population cages over 12 generations. The nuclear genome was standardised to *w*^1118^ and the microbiome controlled each generation by adding a ground homogenate of laboratory males. Given random mating of *Drosophila* harbouring distinct mitotypes [18], population cage studies are a sensitive method to detect positive selection [17, 18].

We investigated whether differences in immature development or adult fitness best described the observed mitotype frequencies on the four diets. Demonstrating that natural selection acts on mitochondrial genes is now firmly established [e.g., 14, 28, 52–56], but the specific life history stages and exogenous conditions through which mtDNA variations benefit the organism have rarely been experimentally identified. When all else is equal, reduced immature development time is beneficial in nature as it reduces exposure to predators and limited food supply [57]. It is also advantageous in population cages if a higher proportion of females of a specific mitotype develop into adults and more eggs are laid. The fitness of young females is experimentally determined by assaying fecundity and fertility. Female fecundity is sensitive to dietary changes and is experimentally measured as the number of eggs laid [13, 14]. Fertility is a central determinant of an animals inclusive fitness and is quantified as the number of offspring, per female [58].

#### Population cages including four mitotypes and four diets

**Fig 1:**
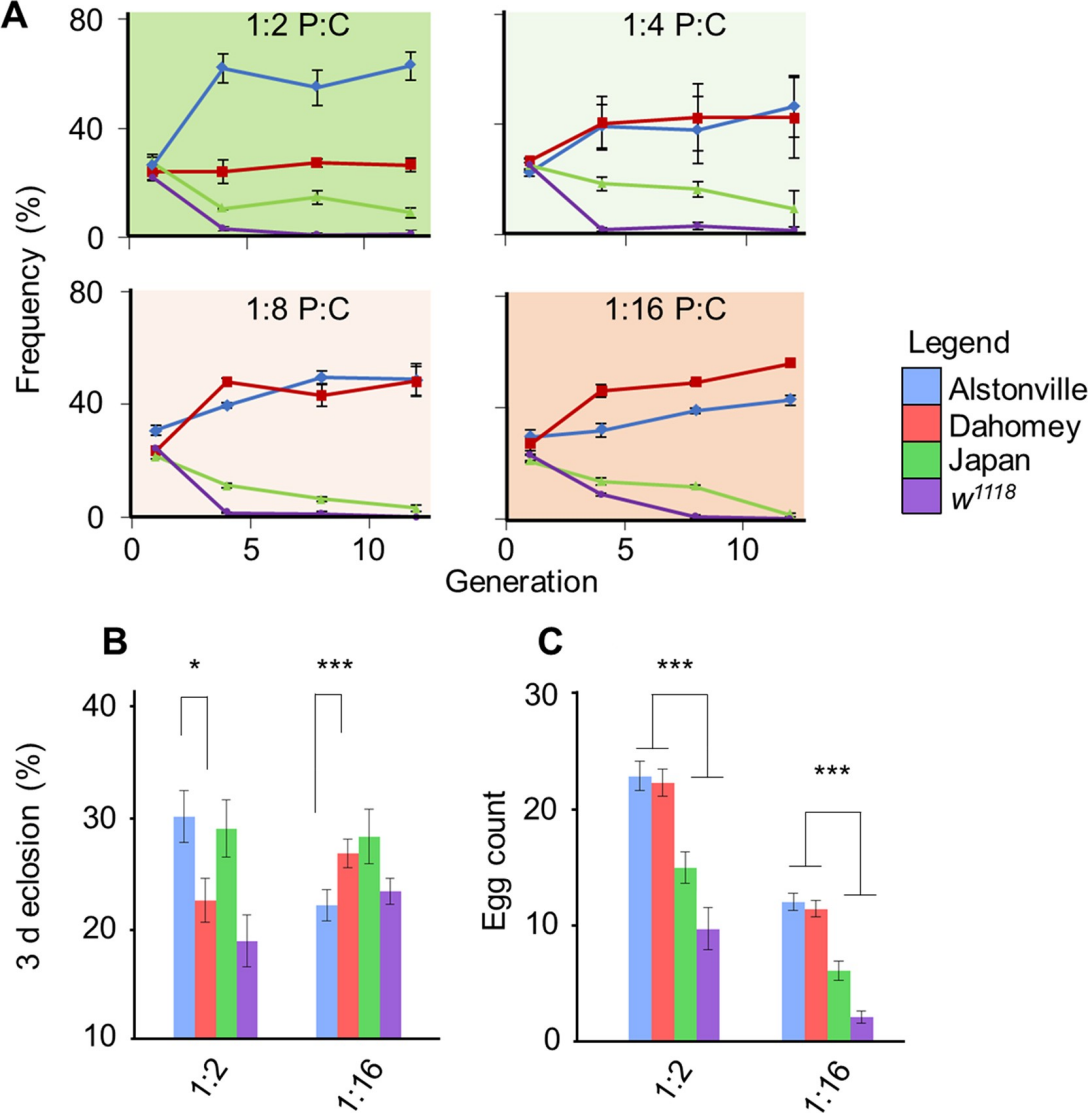
Population cages, larval development and adult fecundity of four mitotypes. Population cage studies, numbers of flies eclosing in 3 d, and egg counts show that larvae harbouring Alstonville mtDNA had an advantage on the 1:2 P:C diet while those with Dahomey mtDNA had the advantage when fed 1:16 P:C food. **(A)** Initial studies competed *D*. *melanogaster w*^1118^ flies harbouring four mitotypes fed four Protein: Carbohydrate (P:C) diets (n = 3 cages/diet). The mitotypes were Alstonville, Dahomey, Japan and *w*^1118^. The P:C ratios were 1:2 (top left), 1:4 (top right), 1:8 (bottom left) and 1:16 (bottom right). The mitotype of 4,608 flies was determined by amplifying ~900bp of mtDNA and then Sanger sequencing. After 12 generations, Alstonville had the highest frequency on the 1:2 P:C diet while Dahomey was highest on 1:16 P:C food. Flies with Japan or *w*^1118^ mtDNA declined in frequency in all diets. Symbols show mean± s.e.m. **(B)** Eclosion percentage in a 3 d window for four mitotypes fed the 1:2 P:C or 1:16 P:C diet (n = 6 bottles/mitotype/diet) with each bottle seeded with 214.5± 14.2 eggs/bottle. t-tests compared Alstonville with Dahomey (see text). **(C)** Flies from each mitotype were assayed for fecundity on the 1:2 P:C and 1:16 P:C diets by egg count over 3 d, with an average of 32 flies/mitotype/diet. t-tests compared Alstonville + Dahomey with Japan + *w*^1118^ (see text). Bars show mean± s.e.m. * p< 0.05, ** p< 0.01, *** p< 0.001.

Flies harbouring Alstonville mtDNA rose to the highest frequency when fed a high protein laboratory diet (1:2 P:C). In contrast, flies with Dahomey mtDNA reached the highest prevalence when fed high carbohydrate food (1:16 P:C) indicating a selective advantage (Fig 1A and S1A & S1B Fig). On all four P:C ratios tested, females with Japan mtDNA decreased in frequency, while those harbouring *w*^1118^ mtDNA went to extinction. The four isocaloric P:C diets were prepared by varying yeast, treacle, semolina and sucrose and resulted in increasing the time to first eclosion of flies suggesting increasing stress with decreasing P:C ratio (~14d, ~18d, ~24d and ~28d on the 1:2, 1:4, 1:8 and 1:16 P:C diets, respectively).

#### Distinguishing immature and adult affects

Differences in immature development time, as evidenced by the number of females eclosing in 3 d, elucidated the fitness differences of Alstonville and Dahomey mitotypes on the 1:2 P:C and 1:16 P:C diets. Overall, ANOVA showed no significant effects for mitotype, diet, or their interaction (F_3_, _40_ = 2.3, p = 0.09; F_1_, _40_ = 3.9, p = 0.06 and F_3_, _40_ = 2.1, p = 0.11, respectively; Fig 1B). We then focused on the Alstonville and Dahomey mitotypes, because cage studies showed they rose to high frequency when fed the 1:2 P:C and 1:16 P:C diets, respectively. In this case, 25% more Alstonville flies eclosed in a 3 d window when fed the 1:2 P:C diet (t_10_ = 2.38, p = 0.04) but 16% more Dahomey eclosed in this period when fed the 1:16 P:C diet (t_10_ = 12.96, p< 0.001; Fig 1B). To determine whether the flip in the numbers of Alstonville and Dahomey eclosing in 3 d resulted from differences in larval development or larval mortality we assayed time to pupation and numbers eclosing in a 6 d window. Alstonville larvae developed to pupation faster than those harbouring Dahomey mtDNA on the 1:2 P:C diet (t_158_ = 3.16, p = 0.02), but Dahomey larvae developed faster than Alstonville when fed the 1:16 P:C food (t_168_ = 3.27, p = 0.001; S1C Fig). Differences in larval development time between the mitotypes were lost when adults were allowed to eclose for 6 d (t_6_ = 0.26, p = 0.80 for the 1:2 P:C diet and t_6_ = 1.00, p = 0.35 for the 1:16 P:C diet; S1D Fig). Therefore, we concluded that the observed differences in the numbers eclosing in 3 d resulted from changes in larval development time.

Differences in adult fecundity explained the decline in the frequency of the Japan and *w*^1118^ mitotypes in cages but did not drive the population cage results for Alstonville and Dahomey. The number of eggs laid by 3–5 d old females in 24 h showed egg production of Japan and *w*^1118^ females was low on both diets and was lower on the 1:16 P:C diet for all mitotypes (F_3_, _257_ = 103.5, p< 0.001; F_1_, _257_ = 344.6, p< 0.001 and F_3_, _257_ = 2.2, p = 0.09, for mitotype, diet and their interaction, respectively; Fig 1C). Comparing Alstonville and Dahomey with Japan and *w*^1118^, the former pair produced ~82% more eggs on the 1:2 P:C diet (t_132_ = 10.78, p< 0.0001) and ~180% more on the 1:16 P:C diet (t_129_ = 12.90, p< 0.0001; Fig 1C). There were no differences in the fecundity or fertility of the Alstonville and Dahomey mitotypes on the 1:2 P:C (t_83_ = 0.49, p = 0.62, t_18_ = 0.91, p = 0.38, respectively) or the 1:16 P:C diet (t_85_ = 0.87, p = 0.38, t_18_ = 0.13, p = 0.9, respectively; Fig 1C and S1E Fig).

#### Summary of Study 1

Here, we have shown that flies harbouring Alstonville and Dahomey mtDNA have a reciprocal fitness advantage when fed the 1:2 P:C and 1:16 P:C diets. The flip in fitness advantage was caused by changes in larval development time and resulted in significant disparities in the number of flies eclosing in 3 d. Towarnicki and Ballard [15] previously reported similar differences in larval development at 23° C and 27° C. Interestingly, however, these differences were lost when larvae were raised at 19° C. Larval development strategies are likely important in nature and have been the subject of multiple experimental evolution studies aimed at understanding the evolutionary strategies associated with adaptations to heterogeneous food supplies [59, 60]. Differences in larval diet have recently been shown to influence adult metabolism and lifespan [61].

We were interested in determining the mechanisms underpinning the flip in frequencies of the Alstonville and Dahomey mitotypes on the 1:2 P:C and 1:16 P:C diets and focus on these mitotypes and diets hereafter. We did not pursue the observed differences in adult fitness. Deleterious mutations are well known to occur in *Drosophila* mtDNA [26, 36–38] and are expected to reduce mitotype fitness, as we observe for Japan and *w*^1118^. Adult males and females of the Japan mitotype had lower levels of complex I mediated oxidative phosphorylation determined from permeabilized fibres than those harbouring the Alstonville mitotype [14]. Further, males harbouring the Japan and *w*^1118^ mitotypes have previously shown high levels of maximum H_2_O_2_ production [62]. In Study 2, we assessed the reproducibility and generality of the results obtained in Study 1.

### Study 2: Population cages, reproducibility, and generalisability including two mitotypes and two diets

We conducted three additional cage studies to corroborate the hypothesis that the fitness of the Alstonville and Dahomey mitotypes was differentially influenced by diet [12]. In the first set of cage studies, we permute the diet to determine whether the mitotype specific responses are generalisable. Here, we include the 1:2 P:C diet for generations 1–4, swapped to the 1:16 P:C diet for generations 4–20, and then returned to the 1:2 P:C diet for generations 20–26. In a second set of cages, we include fruits with ~1:2 and ~1:16 PC ratios. Fruits have previously been used to validate laboratory diets as they effectively control for artificial differences in amino acids, lipids, and micronutrients [63]. We include passionfruit (~1:2 P:C) and banana (~1:16 P:C). In the third set of cages, we compete the two *D*. *melanogaster* mitotypes independently against *Drosophila simulans* (*Wolbachia* uninfected with the *si*III mitotype collected from Kenya [64]). These species are sympatric through large parts of their range and compete for similar resources.

We assay immature development time and test for reproducibility, permute the nuclear genome, replace the laboratory diets with natural fruits and include the microbiome from orchard fed flies. To test for reproducibility, development time was assayed at ~6-month intervals. Mito-nuclear interactions have been shown to influence a range of molecular and organismal traits in insects, crustaceans, fish, and mice [7, 65–68]. Here, we substituted the *w*^1118^ nuclear genome with Oregon R and with Canton S using balancer chromosomes and then conducted five generations of backcrossing before our experiments [7]. The *w*^1118^ nuclear genome diverged from the wild caught Oregon R line in 1984, and they have been separated for more than 800 generations. The Canton S line was collected before 1916 in Canton, Ohio [69]. To corroborate the cage studies that included fruit, we test the development times of the mitotypes fed passionfruit and banana. Host-associated microbiota can impact metabolism and gene expression at cellular and organism-level scales [70, 71]. Adair et al. [72] quantified the bacterial communities associated with natural populations of *D*. *melanogaster* and found microbes were predominantly of two to three taxa. Here, we focus on levels of *Acetobacter* and *Lactobacillus* as they dominated the microbial communities in our populations.

#### Population cages including two mitotypes and two diets

**Fig 2:**
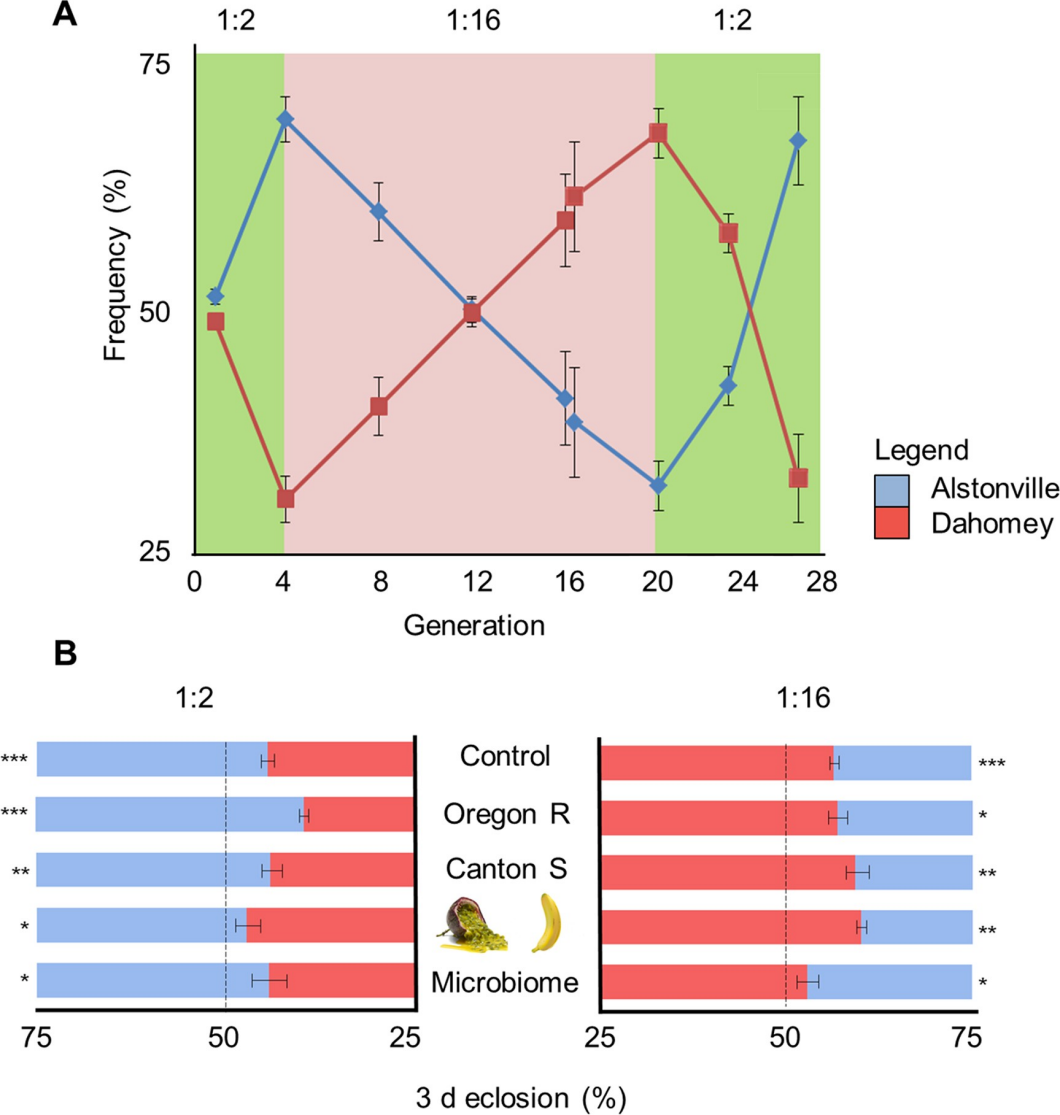
Population cages and larval development of two mitotypes. **(A)** Flies with Alstonville or Dahomey mtDNA were competed (n = 3 cages). Initially, the diet had a 1:2 P:C ratio. After four generations, the diet was switched to 1:16 P:C. An incubator malfunction killed all *Drosophila* in generation 16, so cages were re-established with a similar frequency of each mitotype. In generation 20, the diet was swapped back to 1:2 P:C. Plotted data are mean± s.e.m. mitotype/generation/cage/diet from ~80 flies/cage. **(B)** The number of females eclosing in 3 d was determined. The control was flies with *w*^1118^ nuclear background fed 1:2 P:C and 1:16 P:C laboratory diets (n = 29 replicates of ~80 flies/rep for the 1:2 P:C diet, and n = 48 replicates of ~80 flies/rep for the 1:16 P:C diet). First, the nuclear genetic background was replaced with Oregon R (n = 6 biological rep/mitotype/diet). Second, the nuclear genetic background was replaced with Canton S (n = 5 biological rep/mitotype/diet). Third, passionfruit and banana replaced the laboratory diets (*w*^1118^ nuclear background, P:C ratio of ~1:2 P:C and ~1:16 P:C, respectively; n = 5 rep/mitotype/diet). Finally, the microbiome obtained from wild-caught flies was introduced (*w*^1118^ nuclear background, n = 6 biological rep/mitotype/diet). The 3 d eclosion window began on day 14 for 1:2 P:C diet and day 28 d for 1:16 P:C diet. Plotted data are mean± s.e.m. * p< 0.05, ** p< 0.01, *** p< 0.001 as calculated by t-tests (see text).

Dietary perturbation cage studies conducted over 26 generations support the hypothesis that diet acted as a directional selection pressure on flies harbouring distinct mitotypes (Fig 2A). In all permutations, Alstonville mtDNA had an adaptive advantage when fed the 1:2 P:C laboratory diet (generations 1–4 and 20–26) while Dahomey mtDNA had the advantage when fed 1:16 P:C food (intervening generations; Fig 2A). The fitness difference estimated by the selection coefficient (*s*) was 0.183 on the 1:2 P:C diet and 0.097 on the 1:16 P:C food [73]. To specifically test for the involvement of accumulated nuclear-encoded mutations, we tested the change in frequency of flies during the first 4 and last 6 generations (on the 1:2 P:C diet). This was also tested during intervening generations (on the 1:16 P:C diet), both before and after an incubator malfunction at generation 16. There were no significant differences between slopes for the first 4 and last 6 generations (1:2 P:C diet: Alstonville t_4_ = 0.754, p = 0.49; Dahomey t_4_ = 0.676; p = 0.54); nor for generations 4–16 and 17–20 either side of the incubator malfunction (1:16 P:C diet: Alstonville t_4_ = 0.069, p = 0.95; Dahomey t_4_ = 0.409; p = 0.70). To explicitly test for heteroplasmy and investigate the potential for accumulation of mtDNA mutations, a ~900bp region of mtDNA that had been used to distinguish the mitotypes in the initial study was sequenced from 186 females (62 from each cage) at the conclusion of the study. No heteroplasmy or novel mutations were detected. However, it remains possible that novel mutations occurred outside the region sequenced.

In a second set of cage studies, we controlled for the possible accumulation of strain-specific nuclear encoded mutations and then tested the frequencies of mitotypes fed passionfruit and banana diets. Flies harbouring the two mitotypes were maintained on instant *Drosophila* food for five generations, allowed to mate indiscriminately and isofemale lines were then constructed. Equal numbers of eggs from females harbouring each mitotype then seeded three population cages with passionfruit and three cages with banana. Mitotype frequencies were then assayed. Consistent with the laboratory foods, flies with Alstonville mtDNA increased in frequency when fed passionfruit (*s* = 0.165) while those with Dahomey mtDNA increased in frequency when fed banana (*s* = 0.331).

In the final set of cage studies, the the *D*. *melanogaster* mitotypes were competed against *D*. *simulans*. On both diets, the *D*. *melanogaster* mitotypes outcompeted *D*. *simulans*; however, the rate of increase of Alstonville and Dahomey mtDNA was diet dependent and again consistent with previous results. For Alstonville, *s* was 0.078 higher than Dahomey on the 1:2 P:C diet, but 0.076 lower on the 1:16 P:C food.

#### Reproducibility and generality of immature development

Dietary induced changes in immature development are reproduceable and not sensitive to the nuclear genome, the diet, or the microbiome. Overall, on the 1:2 P:C diet, larvae with the Alstonville mitotype developed 34% faster than Dahomey (t_56_ = 5.48, p< 0.0001). The reverse was true in larvae fed the 1:16 P:C diet where Dahomey developed 30% faster than Alstonville (t_94_ = 12.43, p< 0.0001; Fig 2B). One-way ANOVA on the frequency of Alstonville shows the data are reproduceable over time (for 1:2 P:C diet, 5 time periods F_4,24_ = 2.12, p = 0.11 and for 1:16 P:C food, 8 time periods F_7, 40_ = 0.71, p = 0.67).

Permuting the nuclear genome reproduced the flip in immature development of the two mitotypes on the two diets. There were, however, differences between strains. When the mitotypes were in the Oregon R genetic background and fed the 1:2 P:C diet, ~32% more Alstonville flies eclosed in a 3 d window than Dahomey (t_10_ = 10.30, p< 0.0001; Fig 2B). Conversely, when these larvae were fed the 1:16 P:C diet ~16% more Dahomey eclosed in the window compared to Alstonville (t_10_ = 2.84, p = 0.02; Fig 2B). When the mitotypes were introgressed into the Canton S nuclear genetic background, ~25% more Alstonville eclosed when fed the 1:2 P:C diet (t_8_ = 9.85, p< 0.0001; Fig 2B). Contrariwise, when fed the 1:16 P:C diet ~36% more Dahomey than Alstonville eclosed (t_8_ = 5.31, p = 0.006; Fig 2B).

The specific laboratory diets are not driving the flip in immature development times. Consistent with the population cage results, replacing the laboratory diets with natural fruits corroborated the diet specific flip in the numbers of each mitotype eclosing in 3 d (Fig 2B). When fed passionfruit 21% more Alstonville females eclosed in 3 d (t_8_ = 2.81, p = 0.02). When banana was the food, 48% more Dahomey females eclosed (t_8_ = 5.56, p = 0.001).

We suggest the microbiome is not driving the population cage results above, for two reasons. First, when we aliquoted the homogenate of ground wild-caught males containing an exogenous microbiome into vials containing developing larvae, the mitotype-by-diet interaction was corroborated (t_10_ = 3.28, p = 0.01 and t_16_ = 3.56, p = 0.003 for the 1:2 P:C and 1:16 P:C diets, respectively; Fig 2B). Second, when we examined intragenerational microbiome changes, higher levels of *Acetobacter* were observed in slower developing Alstonville larvae fed the 1:16 P:C food (S2 Fig). *Acetobacter* has previously been associated with faster development in *Drosophila* [74].

#### Summary of Study 2

Here, we conducted perturbation cage studies and repeated the result that flies harbouring Alstonville and Dahomey mtDNA have a reciprocal fitness advantage when fed the 1:2 P:C and 1:16 P:C diets. We then showed the flip in evolutionary advantage was reproduceable and not caused by the nuclear genetic background, the laboratory diet, or the larval laboratory microbiome. The selection coefficients observed here are similar or larger to those experimentally determined in peppered moth *Biston betularia* in England (*s* = 0.1–0.2) [75] and higher than the *D*. *simulans* mtDNA mitotypes in laboratory population cages (*s* = 0.1) [18].

We did not test whether Alstonville or Dahomey goes to extinction on either diet. Warbrick-Smith and colleagues [76] showed that *Plutella xylostella* caterpillars reared for multiple generations on carbohydrate-rich foods progressively developed the ability to consume excess carbohydrate without laying it down as fat. In their study the metabolic adaptation observed may have resulted from selection on the underlying genetic variation and epigenetic changes. Transgenerational epigenetic inheritance is of great interest as it has important evolutionary and human-health implications [77, 78]. Next, in Study 3 we employ quaternary and secondary structure modelling, long read sequencing, cellular assays and protein gels to identify a single point mutation in complex I of Dahomey mtDNA that is likely driving the population cage results.

### Study 3: Testing the significance of candidate mutations

To predict whether a nonsynonymous change, an RNA mutation, or variation in A+T repeat number was likely to be functionally significant we generated quaternary and secondary structure models and then assayed repeat number variation [79–82]. There are three nonsynonymous difference between Alstonville and Dahomey mtDNA [66] and we modelled each complex harbouring a change—complex I (V161L, ND4 subunit), complex IV (D40N, COIII subunit) and complex V (M185I, ATP6 subunit) [79–81]. There are also three rRNA differences (two srRNA and one lrRNA) [83] and 52 A+T-rich region variations [84] (S1 Table). Towarnicki and Ballard [15] mapped the two srRNA mutations on the human mitoribosome and proposed that they are unlikely to influence mitochondrial function [83, 85] so they are not considered here. Selection has rarely been shown to act on the mitochondrial A+T rich or control region [but see, 21, 27], and no differences were identified in secondary structures or the central T-stretch between the Alstonville and Dahomey mitotypes [15]. However, differences in the number of repeats have been recently shown to influence mitochondrial functions [86].

We tested hypotheses generated from the modelling by extracting mitochondria and assaying organelle function, independent of cellular interaction. These cellular assays included electron transport system complex activity assays, *in vitro* mitochondrial oxygen consumption, Western blots and native protein gels. Activity was included because it has previously been employed to corroborate the influence of a mtDNA mutation [87, 88]. We predicted that functionally significant mutations would reduce the activity of the complex. *In vitro* mitochondrial oxygen consumption is increasingly recognised as a fundamental measure of mitochondrial function [89, 90] and we assayed the rate from extracted mitochondria using complex I and II substrates [91]. Complex I substrates assay the combined mitochondrial functions of complexes I, III and IV, while complex II substrates assay the collective functions of complexes II, III and IV. Western blots were used to measure expression of complex I and complex V and native protein gels to determine native protein masses of complex I and its protein–protein complexes.

#### Quaternary and secondary structure modelling and assay for A+T rich repeat number variation

**Fig 3:**
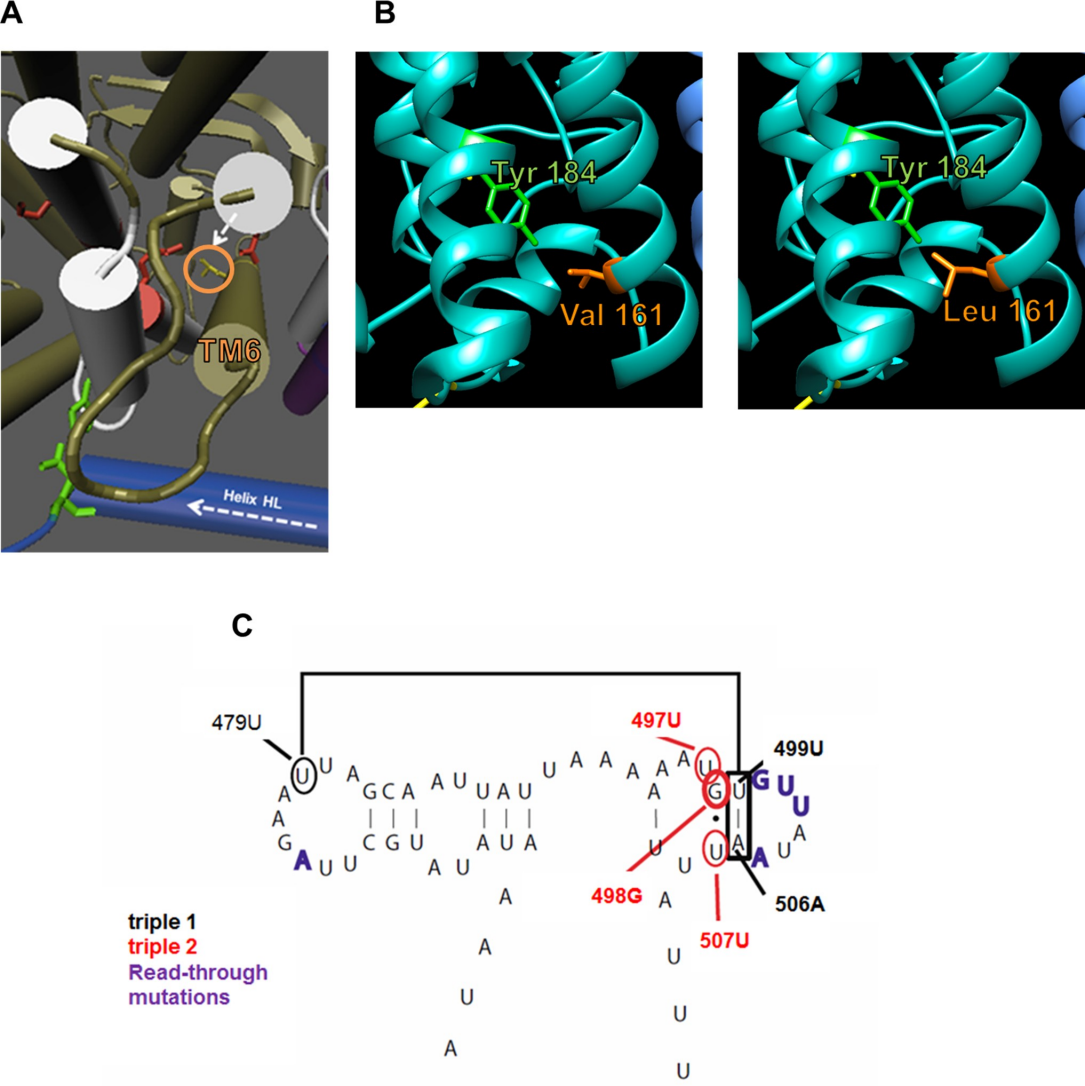
Quaternary and secondary structure modelling. **(A)** The V161L site on TM6 was predicted to slow the cycling of the proton pump. The shorter arrow indicates the movement of TM5 as the Helix HL moves. (**B**) The mutation of site 161 from valine (left panel) to leucine (right panel) likely increases steric hindrance with tyrosine 184, narrowing the proton channel. **(C)** The secondary structure of the GTPase center in the lrRNA of *D*. *melanogaster* showing the site of mutation and structurally related residues.

Structural modelling of the complex I mutation presaged that it reduced proton pumping into the mitochondrial intermembrane space in Dahomey flies (Fig 3A & 3B, S3A Fig). The V161L in the ND4 proton pump corresponds to NuoM 192 in transmembrane domain six (TM6) of *Escherichia coli*, which is adjacent to four highly conserved residues in TM5 of the proton pump [79]. If this model is correct, the V161L site on TM6 will provide increased steric hindrance for the movement of TM5 into this space because the extra methyl group on leucine protrudes further into this space (Fig 3B). This is expected to slow the cycling of the proton pump, which is proposed to initiate the proton movement across the inner mitochondrial membrane. The V161L ND4 site does not physically interact with any nuclear-encoded amino acids, further supporting the hypothesis that nuclear-mitochondrial interactions are not driving the observed effects.

Modelling does not suggest the complex IV or complex V variations are likely to be functionally significant. For the COIII mutation, we visualised the change in complex IV and the supercomplex [80]. The amino acid site faces COX7A, which lies between complex IV and complex I in the supercomplex, but it does not appear to interact with any sites on either subunit (S3A & S3B Fig). For the ATP6 mutation, we investigated the published complex V structure [81]. Unfortunately, the resolution of the structure is not to the amino acid level, and so it is currently not possible to model the M185I, ATP6 change. However, in the currently available model, it is not predicted to interfere with the action of the ATPase itself.

The lrRNA mutation may have a subtle effect on the translation of proteins in the mitochondrion. Heterologous inferential analysis [35] suggested that the lrRNA mutation [83] may plausibly affect the functionally important GTPase center of the mitoribosome and therefore influence protein translation (Fig 3C). Two conserved base triplets were observed in tandem in this region (triple 1 and triple 2). The G498A mutation in Dahomey replaced a G•U wobble with a canonical A:U within triple 2, potentially disrupting its structure. Given the functional importance of the region, it is possible that even a small distortion in its structure, due to the G498A base change, might affect mito-ribosomal function. Despite this structural prediction *Cellular assays* (see below), indicate the lrRNA mutation is unlikely to be driving the population cage results.

We conducted continuous long-read Pacific BioSciences sequencing of the Alstonville and Dahomey mtDNA genomes to determine whether there was variation in the number of A+T repeats between the mitotypes [86]. These analyses showed no differences from the published short-read A+T rich region sequences [84] and no differences in repeat number between mitotypes.

#### Cellular assays

**Fig 4:**
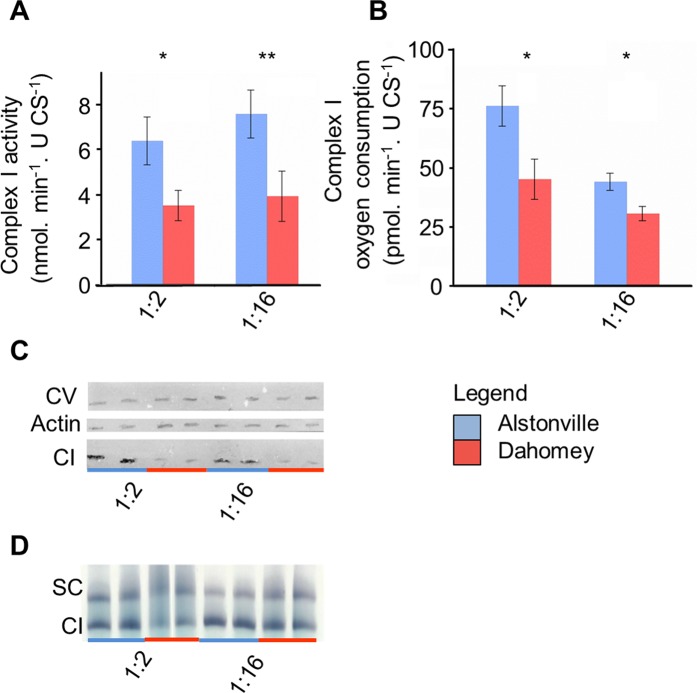
The V161L ND4 amino acid change in complex I of Dahomey mtDNA influenced mitochondrial functions. **(A)** The activity of complex I is higher in Alstonville larvae (n = 7 biological rep/mitotype/diet- with two failed reactions removed). **(B)** Oxygen consumption rate with complex I substrates is higher in Alstonville larvae (n = 6 biological rep/mitotype for 1:2 P:C diet; n = 9 biological rep/mitotype for 1:16 P:C diet). **(C)** Representative western blot showing reduced levels of complex I (CI) in Dahomey compared to Alstonville on both diets. There were no obvious differences in complex V (CV) or actin protein levels. Repeat gels showed all CI bands were the same size. **(D)** Native protein gel showing reduced activity of the peripheral arm of complex I (CI) and the supercomplex (SC) in larvae harbouring Dahomey mtDNA on both diets. Bars (mean± s.e.m). t-tests between mitotypes * p< 0.05, ** p< 0.01 (see text).

Activity assays supported the hypothesis that Dahomey larvae harbour a compromised complex I. Complex I activity in Dahomey was ~53% lower than Alstonville on both diets (Fig 4A). ANOVA showed a significant effect of mitotype, but not diet, nor their interaction (F_1_, _24_ = 15.98, p = 0.0005; F_1_, _24_ = 0.35, p = 0.56 and F_1_, _24_ = 0.38, p = 0.54, respectively). t-tests showed significant differences between the mitotypes on the 1:2 and 1:16 P:C diets (t_12_ = 2.53, p = 0.026 and t_12_ = 3.10, p = 0.009, respectively). The nonsynonymous mutations in COIII and ATP6 did not affect activity of complexes IV and V further suggesting they were not functionally significant. There was no effect of mitotype in either complex (F_1_, _16_ = 1.83, p = 0.20, F_1_, _22_ = 1.17, p = 0.29 for complexes IV and V, respectively; S4A & S4B Fig). Both complexes showed higher activity when larvae were fed the 1:2 P:C diet (F_1_, _16_ = 11.45, p = 0.004; F_1_, _22_ = 32.12, p< 0.0001 for complexes IV and V, respectively; S4A & S4B Fig). There were no significant mitotype-by-diet effects in either complex (F_1_, _16_ = 0.48, p = 0.49; F_1_, _22_ = 0.02, p = 0.88; for complexes IV and V, respectively; S4A & S4B Fig).

Mitochondrial oxygen consumption data provide further evidence to suggest that Dahomey larvae harbour a compromised complex I. The prediction is that a slightly deleterious ND4 mutation would decrease oxygen consumption from complex I, but not affect oxygen consumption from complex II substrates. In contrast, the lrRNA variant may be expected to decrease oxygen consumption from both complex I and II substrates. When complex I substrates were included, oxygen consumption in mitochondria extracted from Alstonville was ~69% higher than Dahomey for larvae fed the 1:2 P:C diet and ~44% higher than Dahomey for the 1:16 P:C diets (Fig 4B). Overall, larvae had 63% higher oxygen consumption on the 1:2 P:C diet. ANOVA showed significant effects of mitotype and diet, but the interaction was not significant (F_1_, _26_ = 16.08, p< 0.001; F_1_, _26_ = 17.69, p< 0.001 and F_1_, _26_ = 2.51, p = 0.126, respectively). t-tests showed significant differences between the mitotypes on both diets (t_10_ = 2.70, p = 0.022 and t_16_ = 2.69, p = 0.02, for the 1:2 and 1:16 P:C diets respectively). For the complex II substrates, there were no clear differences between mitotypes, but larvae fed the 1:2 P:C diet had more than double the oxygen consumption than larvae fed the 1:16 P:C food (S4C Fig). ANOVA showed a significant effect of diet (F_1_, _20_ = 39.18, p< 0.0001) but no significant difference in oxygen consumption between the mitotypes (F_1_, _20_ = 0.15, p = 0.70) or significant mitotype by diet interaction (F_1_, _20_ = 0.38, p = 0.54).

Western blot analysis shows reduced complex I protein in Dahomey as compared to Alstonville on both diets, but no obvious differences in ATP synthase between the mitotypes (Fig 4C). Native protein gel analysis shows the peripheral arm of complex I as well as the supercomplex have reduced activity in Dahomey larvae on both diets (Fig 4D). These data corroborate the hypotheses that Dahomey larvae harbour a functionally compromised complex I and strongly suggest the lrRNA variant has, at most, subtle effects.

#### Summary of Study 3

Complex I is a crucial enzyme in oxidative phosphorylation. It uses NADH oxidation and ubiquinone reduction to build the proton motive force across the mitochondrial inner membrane, which catalyzes respiration and drives ATP synthesis [92, 93].The ND4 V161L mutation in Dahomey flies reduced complex I activity by about 56% and caused a decline in frequency in population cages with 1:2 P:C food. In contrast, we propose that a 1:16 P:C diet triggers a compensatory mitohormetic response in these larvae that results in a net benefit and increased frequency in the population cages. Positive Darwinian selection has previously been detected in the mitochondrial‐encoded subunits that comprise complex I from diverse taxa with seemingly dissimilar bioenergetic life histories [28]. The observed reduction in complex I activity suggests the mutation is mildly deleterious and not disease causing. In nonsynaptic rat brain mitochondria complex I could be decreased by 72% before disease-like changes in mitochondrial respiration took place [94].

We suggest the Dahomey lrRNA mutation causes a negligible or, at most, subtle effect. In the *Cellular assays* section, we did not find any evidence to indicate the lrRNA mutation influenced mitochondrial functions or levels of electron transport system proteins. In Study 5, we assay RNA expression and do not find evidence for significant upregulation of genes involved in mitoribosome structure in Dahomey larvae. Conversely, there is evidence for significant upregulation of ribosome related genes in Alstonville larvae fed the 1:16 P:C diet. It is believed that insults to mitochondrial translation should result in detectable retrograde signalling events [95]. However, the nature of these signalling events has not yet been elucidated. In bacteria, mutations at neighbouring residues have been shown to cause readthrough of termination codons without noticeable growth effects in some cases [96]. Fully understanding the impact of the lrRNA mutation would likely require its recreation in a heterologous organism like *E*. *coli* [97].

Although strongly indicative, Study 3 assays do not confirm that the ND4 mutation alone, is driving the population cage results. As suggested, the lrRNA mutation may have caused subtle effects and substitutions in the A+T region may have had a functional impact that is difficult to detect [98]. Further, all mitotypes were derived from wild-caught lines and it is possible that a co-segregating maternally inherited factor (such as a virus) could have influenced the population cage and development time results [99]. Next, we experimentally address these concerns by adding complex I inhibitors to the diet and include two other mitotypes to test whether the diet specific flip in immature development time is specific to Alstonville and Dahomey.

### Study 4: An electron transport system complex I mutation in Dahomey drives the population cage results

Chemical impairment of complex I reproduces the observed flip in development rates. Here, complex I inhibitors were added to the diet to create phenocopies in Alstonville of the Dahomey ND4 mutation. Goldschmidt [100] coined the term “phenocopy” to describe morphological alterations in *Drosophila* that could be induced by the imposition of stress during development. Thus, a phenocopy is produced environmentally and shows features characteristic of a genotype other than its own. Chemically induced phenocopies in *Drosophila* are well studied with the production of eyeless mutants by feeding food containing borate [101] and production of bithorax mutants by treating embryos with diethyl ether [102]. We added rotenone to the diet to phenocopy the ND4 mutation in Dahomey because it inhibits electron transfer from the iron-sulphur centres in complex I, leading to a partial blockade of oxidative phosphorylation with reduced synthesis of ATP [103]. We then quantify the rate of development. If the slightly deleterious V161L ND4 mutation in Dahomey was driving the differences in development time (Fig 1B), we predicted that Alstonville larvae fed food containing rotenone (the phenocopy) would develop more slowly than untreated larvae on the 1:2 P:C diet, but faster on the 1:16 P:C food. In contrast, we predicted that Dahomey larvae fed rotenone would develop more slowly when fed both diets as the complex I dysfunction would be the combined effects of the mutation and the inhibitor. We then tested the generality of the rotenone result with paraquat. Paraquat is a common herbicide that has been proposed to cause mitochondrial dysfunction by complex I toxicity following lipid peroxidation of the mitochondrial inner membrane [104].

Next, we tested whether dietary addition of rotenone influenced complex I activity, superoxide dismutase (SOD) activity, and larval dry weight. Complex I activity in larvae fed the standard diets was measured in Study 3 (Fig 4A). It was included here to test whether the ND4 mutation and the dietary addition of rotenone had similar effects on the complex. SOD constitutes the first line of defence in the antioxidant enzyme network [105, 106], is the primary scavenger of the ROS superoxide [107], and total activity was assayed. Larval weight was assayed as an organismal trait that can influence development time [reviewed in 108] and patterns of adult reproductive investment [109].

To test whether the flip in immature development time was generalisable to a second pair of mitotypes, one of which harboured the V161L ND4 mutation, we compared the immature development times of flies harbouring Madang (Papua New Guinea) and Victoria Falls (Zimbabwe, Africa) mtDNA [84]. Madang mtDNA has the same ND4, and lrRNA mutations and differs from Dahomey by 27 A+T rich region mutations (S1 Table). Victoria Falls does not harbour either the ND4 or the lrRNA mutations. It has three nonsynonymous (ND2, ATP6 and COIII), two sRNA and 49 A+T rich region differences from Alstonville (S1 Table). For experimentation, both mitotypes were harboured in the *w*^1118^ nuclear genetic background and the microbiome was controlled.

#### Effect of dietary inhibitors on immature development

**Fig 5:**
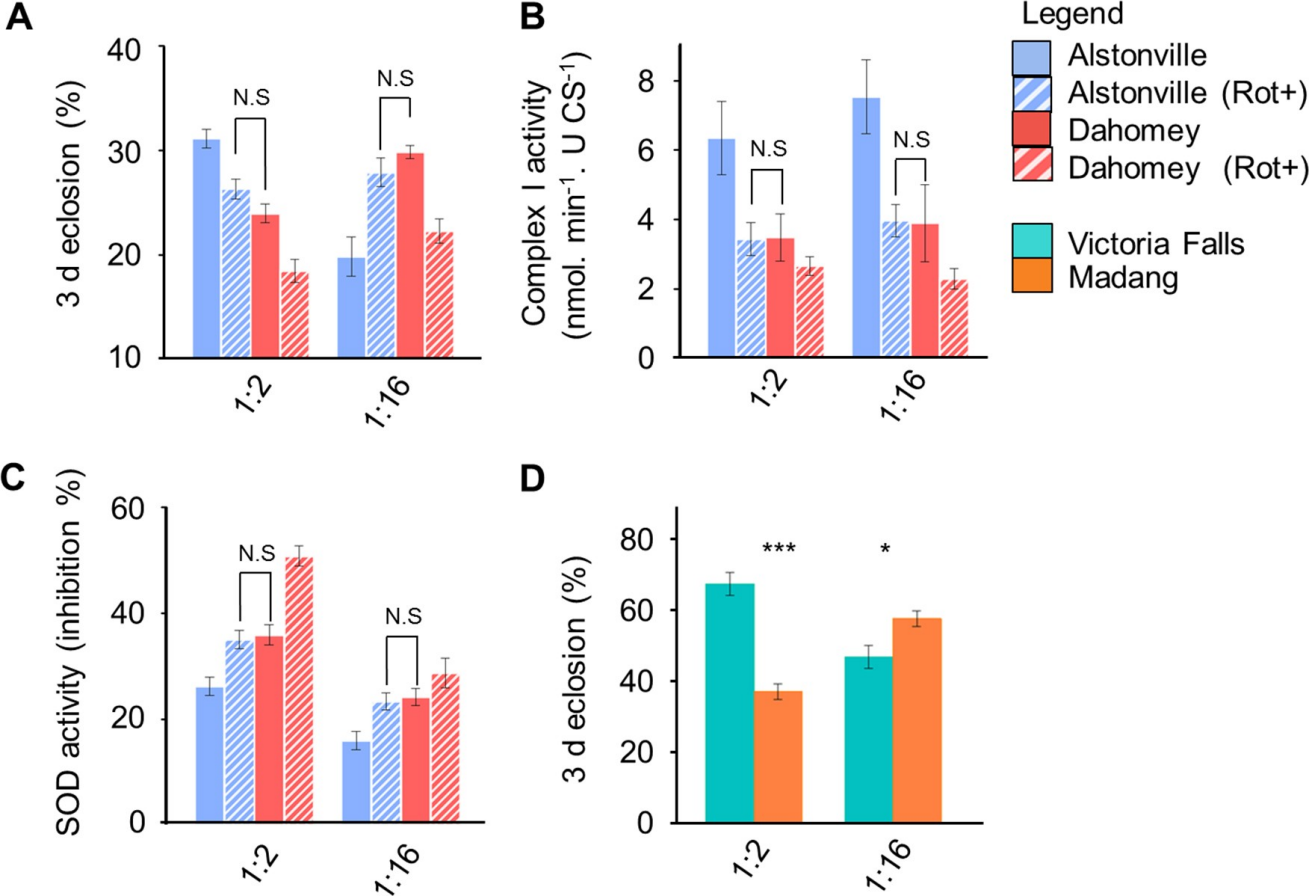
Corroboration that the complex I mutation in Dahomey drives the population cage results. (**A**) Adding rotenone to the Alstonville diet created a Dahomey phenocopy. This phenocopy developed more quickly than controls when fed the 1:16 P:C food showing that partial inhibition of complex I was beneficial. Adding rotenone to the Dahomey fly food created a disease model and these larvae developed more slowly on both diets (n = 5 biological rep/mitotype/diet with and without rotenone treatment). (**B**) Complex I activity was decreased in the phenocopy, mimicking the Dahomey mitotype (n = 7 biological rep/mitotype/diet without rotenone treatment and 6 biological rep/mitotype/diet with rotenone treatment). (**C**) SOD activity increased in rotenone-treated larvae. On both foods, SOD activity in the phenocopy was not different from the Dahomey mitotype (n = 5 biological rep/mitotype/diet with and without rotenone treatment). (**D**) Larval development times of *D*. *melanogaster* harbouring the Madang (with the V161L ND4 mutation) and the Victoria Falls (without the ND4 mutation) mitotypes shows the same flip in development times as Dahomey and Alstonville (n = 6 bottles/mitotype/diet). Plotted data were mean± s.e.m. * p< 0.05 and ** p< 0.001, as calculated by t-tests (see text). Note: complete *post-hoc* analyses including all treatments for panels A-C are presented in S5 Fig.

In terms of immature development, dietary addition of rotenone caused Alstonville larvae to phenocopy development rates of Dahomey fed the standard diets (Fig 5A, S5A Fig). Specifically, addition of rotenone to the 1:2 P:C diet caused fewer Alstonville larvae to eclose in 3 d than the untreated control. Conversely, more eclosed when the inhibitor was added to the 1:16 P:C diet. ANOVA showed that rotenone treatment had a significant main effect on numbers eclosing (F_1,32_ = 14.65, p = 0.001; Fig 5A, S5A Fig), but mitotype and diet did not (F_1,32_ = 2.09, p = 0.16; F_1,32_ = 1.67, p = 0.20. respectively). The two-way interactions of mitotype-by-diet and mitotype-by-rotenone significantly affected the numbers of flies eclosing (F_1_, _32_ = 11.75, p = 0.002; F_1_, _32_ = 5.81, p = 0.02, respectively), while the diet-by-rotenone interaction did not (F_1_, _32_ = 3.25, p = 0.08). The three-way interaction also significantly affected the numbers of flies eclosing (F_1,32_ = 6.31, p = 0.01). As we were particularly interested in testing whether rotenone treatment of the Alstonville mitotype phenocopied Dahomey, we performed t-tests of these treatments. t-tests show no significant differences between the Dahomey control and Alstonville rotenone treatment on the 1:2 P:C diet (t_8_ = 0.61, p = 0.56) nor the 1:16 P:C food (t_8_ = 1.25, p = 0.25).

To test the generality of the rotenone phenocopy we added paraquat to the diets and assayed larval development. As expected, Alstonville larvae fed food treated with paraquat phenocopied Dahomey fed the standard diet. Addition of paraquat to the 1:2 PC diet caused a 40% decrease in the proportion of the phenocopy developing in 3 d. Conversely, when paraquat was added to the 1:16 P:C diet the proportion of the phenocopy developing in 3 d increased by 25% (S6 Fig). Therefore, we concluded that adding paraquat to the diets produced similar results to that induced by rotenone (Fig 5A, S5A Fig, S6 Fig). Undesirably, paraquat caused ~40% mortality and so it was not studied further.

#### Effect of rotenone on complex I activity, SOD activity and larval weight

As reported in Study 3, complex I activity was higher in Alstonville than Dahomey (Fig 4A). Adding rotenone to the diets of Alstonville larvae decreased complex I activity to levels that were similar to those of Dahomey (Fig 5B, S5B Fig). Adding rotenone to the diets of Dahomey larvae further decreased complex I activity. ANOVA showed significant effects of mitotype and rotenone treatment (F_1_, _44_ = 20.55, p< 0.0001 and F_1_, _44_ = 11.05, p = 0.002, respectively) but no main effect of diet (F_1_, _44_ = 0.34, p = 0.56). Complex I activity was significantly affected by the mitotype-by-rotenone interaction (F_1_, _44_ = 5.61, p = 0.02), but not by the mitotype-by-diet or the diet-by-rotenone interactions (F_1_, _44_ = 0.85, p = 0.36; F_1_, _44_ = 0.20, p = 0.66, respectively). The three-way interaction was not significant (F_1_, _44_ = 0.029, p = 0.89). Next, we performed t-tests to determine whether rotenone treatment of the Alstonville mitotype phenocopied Dahomey. As expected, there was no significant difference on the 1:2 P:C (t_11_ = 0.49, p = 0.63) or the 1:16 P:C diet (t_11_ = 0.88, p = 0.40).

When larvae were fed the standard diet, SOD activity was 42% higher in Dahomey larvae than Alstonville larvae and 54% higher in mitotypes fed the 1:2 P:C diet (Fig 5C, S5C Fig). Adding rotenone to the diets of Alstonville larvae caused them to phenocopy Dahomey larvae (Fig 5C, S5C Fig). Dietary addition of rotenone to Dahomey caused further increases in SOD activity. ANOVA showed significant main effects of mitotype, diet, and rotenone treatment (F_1,32_ = 25.82, p< 0.0001; F_1,32_ = 52.53, p< 0.0001; F_1,32_ = 21.56, p< 0.0001, respectively). SOD activity was not significantly affected by any interaction (F_1_, _32_ = 2.40, p = 0.13; F_1_, _32_ = 0.18, p = 0.67; F_1_, _32_ = 2.33, p = 0.14, F_1,32_ = 1.35, p = 0.25 for mitotype-by-diet, mitotype-by-rotenone, diet-by-rotenone, and the three-way interaction, respectively). t-tests showed Alstonville treated with rotenone did not differ significantly from the Dahomey control on either diet (t_8_ = 0.28, p = 0.79; t_8_ = 0.18, p = 0.86. for the 1:2 and 1:16 P:C diets, respectively).

Our prediction that adding rotenone to the diet of Alstonville flies would phenocopy the larval weights of Dahomey flies was not observed. Larval weights were similar when fed the 1:2 P:C diet but Dahomey were 21% heavier than Alstonville when fed the 1:16 P:C food (S5D Fig). Addition of rotenone to the 1:2 P:C diet, caused both mitotypes to be ~27% lighter than the non-rotenone treated control. Further, addition of the inhibitor to the 1:16 P:C food caused Dahomey to be ~24% lighter than those not fed the inhibitor (S5D Fig). Rotenone did not cause a dramatic reduction in the weight of Alstonville larvae fed the 1:16 P:C food. ANOVA showed a significant effect of diet and rotenone treatment (F_1,36_ = 6.87, p = 0.013; F_1,36_ = 47.44, p< 0.0001, respectively) but not of mitotype (F_1,36_ = 0.39, p = 0.54). The two-way interaction of mitotype-by-rotenone significantly affected larval weight (F_1_, _36_ = 9.28, p = 0.004), while the interactions between mitotype-by-diet, diet-by-rotenone and the three way interaction did not (F_1_, _36_ = 0.04, p = 0.85; F_1_, _36_ = 1.25, p = 0.27; F_1,36_ = 4.10 p = 0.05, respectively). There was a significant difference in the weights of Alstonville larvae fed rotenone-treated food and the Dahomey control on both diets (t_8_ = 2.39, p = 0.04 and t_8_ = 2.76, p = 0.02, 1:2 and 1:16 P:C, respectively).

#### Testing development times with a second pair of mitotypes

We examined the immature development times of the larvae harbouring Madang (with the V161L ND4 mutation) and Victoria Falls (without the ND4 mutation) mtDNA. Consistent with the expected influence of the ND4 mutation 46% fewer Madang eclosed in 3 d on the 1:2 P:C diet, but 21% more eclosed when fed the 1:16 P:C diet (Fig 5D). ANOVA showed a significant effect of mitotype and an interaction between mitotype and diet on larval development time (F_1_, _20_ = 16.98, p = 0.0005; F_1_, _20_ = 60.80, p< 0.0001, respectively). Diet had no main effect (F_1_, _20_ = 0, p = 1.0). As expected, t-tests showed significant differences between the mitotypes on the 1:2 and 1:16 P:C diets (t_10_ = 7.91, p< 0.0001 and t_10_ = 2.79, p = 0.019, respectively; Fig 5D).

#### Summary of Study 4

We propose that the data from the Dahomey phenocopies provide additional layers of support to the tenet that the ND4 mutation drove the population cage results. Dahomey phenocopies and Dahomey larvae showed a similar flip in the relative development times on the two diets, had a similar reduction in complex I activity and a comparable elevation of SOD activity. Larvae fed food supplemented with rotenone were, however, lighter in three of the four comparisons suggesting the mechanism of action of rotenone and the consequences of the ND4 mutation are not identical. A reduction in the weight of rotenone-treated rats has previously been reported with microarray data showing differences in transcriptional regulation and regulation of cell death/apoptosis [110, 111]. Rotenone is also known to inhibit microtubule assembly [112], and chronic levels have been shown to recapitulate critical aspects of Parkinson’s Disease in *Drosophila* adults [113].

Comparison of the immature development times of Madang and Victoria Falls generalise the diet-specific flip in development time results to a second pair of mitotypes. A limitation of this comparison is that the ND4 and lrRNA mutations, as well as 27 A+T region mutations, are common to Madang and Dahomey. It is not known if these mutations occurred independently or these mitotypes have a shared evolutionary history. Future studies may explore naturally occurring variation in West Africa and Papua New Guinea to determine if the ND4 and lrRNA mutations are completely linked in nature.

Additional strategies to experimentally test the consequences of the ND4 mutation would be to manipulate the Alstonville mtDNA genome and add substrates to complex II. Progress in the area of mtDNA manipulation has been glacial. The claim that the mitochondrial genome can be edited by CRISPR/Cas9 [114] has been questioned and the existence of an endogenous mechanism for nucleic acid import into mitochondria remains controversial [115]. Employing targeted restriction enzymes to manipulate mtDNA is a very clever strategy but is limited by the locality of restriction sites and the low survival of germline precursor cells [116]. An alternative approach may be to create a heteroplasmic fly line with both Dahomey and Alstonville mtDNA using microinjection [23], and then manipulate the heteroplasmy of these two mtDNA’s using engineered nucleases such as mtZFN and mitoTALEN [115]. Adding substrates to complex II has strong potential to by-pass complex I. Unfortunately, the currently available complex II pro-drugs are optimised for *in vitro* use, have a half-life ranging from a few seconds to a few minutes and also release aldehydes [117]. Replacing sucrose with succinate in the diet is not expected to provide substrates to mitochondrial complex II as succinate is a substrate for intestinal gluconeogenesis [118] and exogenously given succinate has limited uptake into cells [117, 119]. Next, we conduct transcriptomics and metabolomics assays to start unravelling the mechanisms underpinning the dietary effects. We report the results for Alstonville before Dahomey when larvae were fed the 1:2 P:C diet. Conversely, we report Dahomey first when larvae were fed the 1:16 P:C food. We take this approach because Alstonville increased in frequency on the former diet while Dahomey had the advantage on the latter food.

### Study 5: Transcriptomics and metabolomics

To gain mechanistic insight into the processes underpinning the flip in the development times of the mitotypes we include transcriptomics and metabolomics studies. Next-generation RNA sequencing has permitted the mapping of transcribed regions of the genomes of a variety of organisms [120–122]. Studies of *Drosophila* reveal a transcriptome of high complexity that is expressed in discrete, tissue- and condition-specific mRNA and ncRNA transcript isoforms [120]. This enables a dynamic ensemble of transcript isoforms that gives rise to substantial diversity. Recently, Crofton and colleagues [123] asked whether *D*. *melanogaster* mothers who experience poor nutrition during their own development change their gene product contribution to the egg. They find an increase in transcripts for transport and localization of macromolecules and for the electron transport chain. In this study flies were raised for at least two generations on instant *Drosophila* food. Eggs were then transferred to each diet and a standard microbiome added after 2 d. A limitation of the technique is that not all transcripts currently have a known function.

Metabolomic profiling provides an additional layer of knowledge for the most complete representation of the phenotype of the animal, revealing the combined contributions of gene expression, enzyme activity, and environmental context [124]. Here, we include gas chromatography-mass spectroscopy (GC/MS), which is capable of measuring small molecules with a mass <500 Da. One constraint of the GC/MS method for metabolomics studies is that distinct molecules may have similar retention times and it is necessary to validate results with standards [125].

#### Transcriptomics

**Fig 6:**
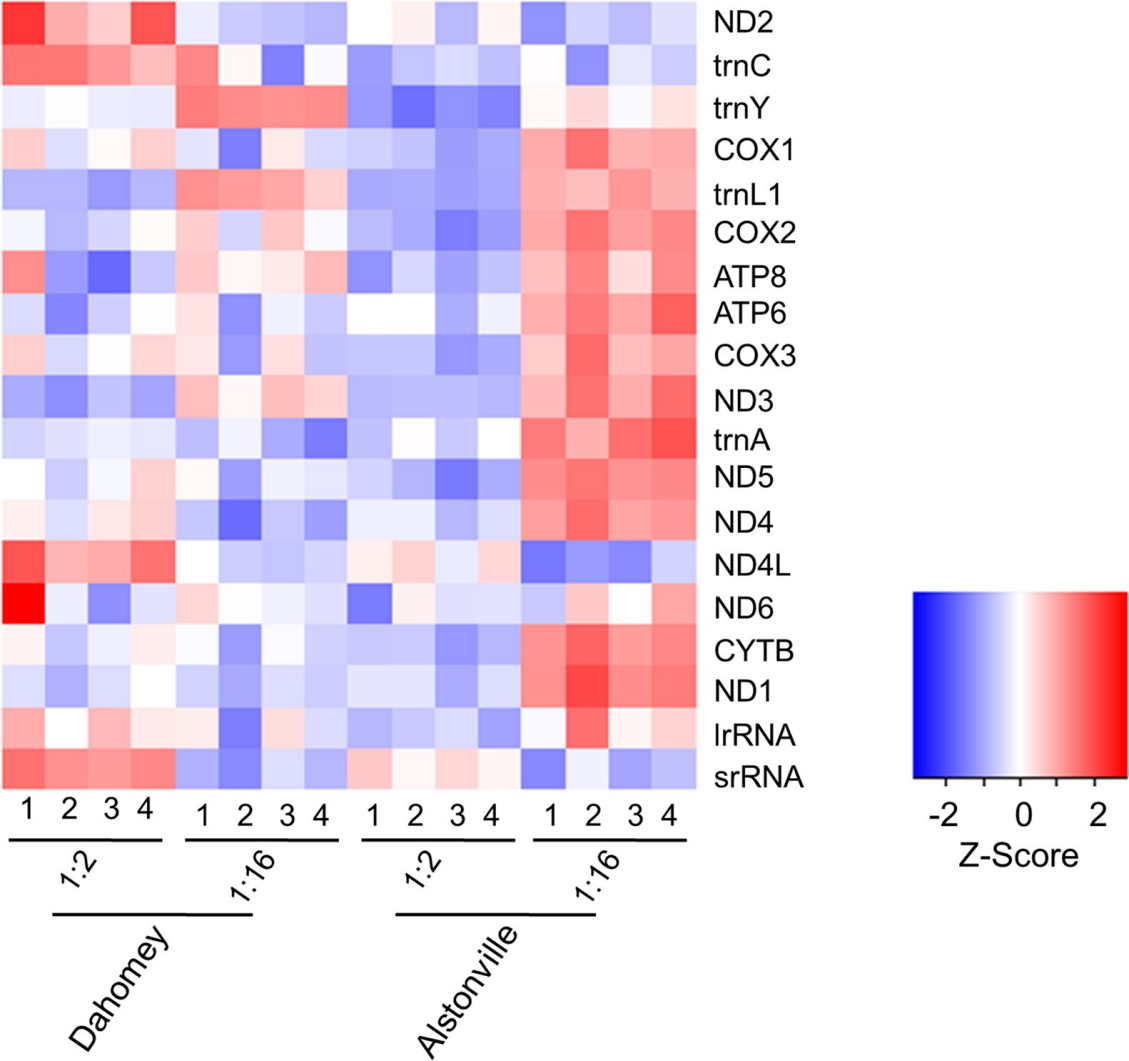
Heat map showing relative expression of mtDNA encoded genes in third instar female wandering larvae harbouring Dahomey and Alstonville mtDNA that were fed on 1:2 P:C and 1:16 P:C ratio diets. Within each treatment, there were four replicates (1–4). Log-expression values were batch corrected and standardised by gene. The darker the blue the more negative the Z score. The darker the red the more positive the Z score.

RNA-seq profiling suggested the expression of mtDNA-encoded genes was diet-dependent and could be differently regulated at the level of transcription in *Drosophila*. Over the whole genome, 1208 (S2A & S2B Table) genes showed a significant interaction between diet and mitotype meaning that they were differentially expressed in a diet-dependent manner (FDR< 0.05). For the mtDNA encoded genes, the expression levels tended to be diet-dependent and were not consistently up in one mitotype (Fig 6). Most mtDNA-encoded genes were downregulated in larvae with Alstonville mtDNA fed 1:2 P:C diet and upregulated in these larvae fed the 1:16 P:C food. The mtDNA-encoded genes of Dahomey fed 1:2 P:C and 1:16 P:C diets were expressed at intermediate levels (Fig 6).

**Fig 7:**
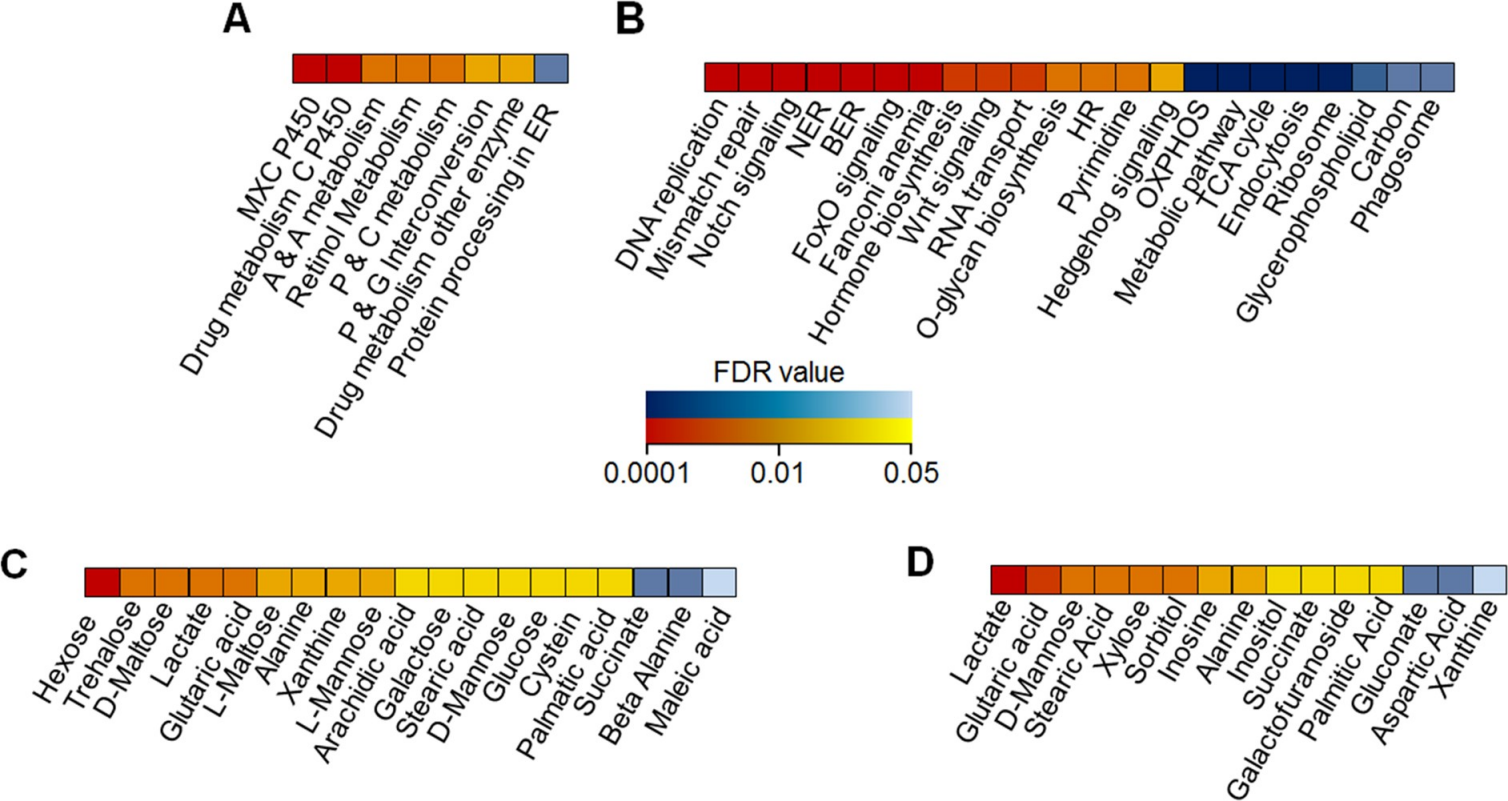
Transcriptomics and metabolomics assays. Differentially expressed KEGG pathways and metabolites from wandering third instar female larvae fed the 1:2 P:C and 1:16 P:C food. Red indicates elevated in Dahomey, blue elevated in Alstonville with darker colours representing smaller FDRs. Detailed FDRs are shown in S3 Table. (**A**) The differentially expressed KEGG pathways observed in RNA-seq profiling for the 1:2 P:C diet (n = 4 biological rep/mitotype). MXC P450 is Methoxychlor-Cytochrome P450, C P450 is Cytochrome P450, A & A is Ascorbate and Aldarate, P & C is Porphyrin and Chlorophyll, P & G is Pentose and Gluconate, ER is endoplasmic reticulum. (**B**) The differentially expressed KEGG pathways observed in RNA-seq profiling for the 1:16 P:C diet (n = 4 biological rep/mitotype). NER is nucleotide excision repair, BER is base excision repair, FoxO is Forkhead box, HR is homologous recombination, Pyrimidine is pyrimidine metabolism, OXPHOS is oxidative phosphorylation, TCA is tricarboxylic acid, Glycerophospholipid is glycerophospholipid metabolism, and Carbon is carbon metabolism. Detailed FDRs are shown in S5 Table. **(C)** Differentially abundant metabolites observed in GC/MS profiling (n = 5 biological rep/mitotype) for the 1:2 P:C diet. (**D**) Differentially abundant metabolites observed in GC/MS profiling (n = 5 biological rep/mitotype) for the 1:16 P:C diet.

For the 1:2 P:C diet, 93 genes were differentially expressed (S2A Table). On this high protein diet, there was one differentially upregulated KEGG pathway (p< 0.01) and seven differentially downregulated KEGG pathways (p< 0.01) in Alstonville compared to Dahomey larvae (Fig 7A and S3A & S3B Table). Of those upregulated in Dahomey, at least two (MXC P450 and Drug Metabolism C P450) are linked with a stress response [126], suggesting these larvae were oxidatively stressed. Gene Ontology analyses of the two mitotypes on the 1:2 P:C diet (S4 Table) shows the molecular function of “pyruvate dehydrogenase kinase activity” (GO:0004740) is reduced in Dahomey (S4B Table), suggesting reduced activity of the electron transport system in these larvae on this diet. There is no evidence for the differential expression of genes encoding the mitochondrial ribosome in larvae harbouring the two mitotypes.

For the 1:16 P:C food, 3114 genes were differentially expressed suggesting extensive remodelling of metabolism (S2B Table). There were 14 differentially upregulated KEGG pathways (p< 0.01) and eight differentially downregulated KEGG pathways (p< 0.01) in Dahomey compared to Alstonville larvae (Fig 7B and S3C & S3D Table). Of the 14 differentially upregulated pathways in Dahomey at least three (replication, Notch, and FOXO signalling) have been associated with development [127, 128]. Gene Ontology analyses of the two mitotypes on the two diets shows annotations with low probability (S4C & S4D Table). Comparing Dahomey with Alstonville the most upregulated was the cellular component “nucleus” (GO:0005634) (S4C Table). Plausibly, this suggests a broad anterograde response to reduced mitochondrial function. Significantly downregulated terms in Dahomey include the cellular component “mitochondrion” (GO:0005739) (S4D Table). Further, the molecular function of “NADH dehydrogenase activity” (GO:0003954) is down in Dahomey (S4D Table), corroborating the advantage of Dahomey flies raised on the 1:16 P:C diet is independent of complex I activity.

The most significantly upregulated KEGG pathway in Alstonville was “oxidative phosphorylation" (Fig 7B). Furthermore, the cellular component terms "large ribosomal subunit" (GO:0015934) and “mitochondrial ribosome” (GO:0005761) are strongly up-regulated on the 1:16 P:C diet compared with the 1:2 P:C diet (p = 3.53e-53 and 5.50e-37, respectively). Elevated expression of multiple mitochondrially encoded genes (Fig 7B), *mitochondrial RNA polymerase (mtRNApol)* [129] and the dual regulator of *mitochondrial transcription termination factor 3 (mTerf3)* [130], actively support the result that a general increase in mitochondrial gene expression is part of Alstonville rewiring on the 1:16 P:C diet (S2B Table). The high number of upregulated mitochondrial ribosomal proteins and of *mitochondrial translation elongation factor Tu1* (*mEFTu1)* strongly suggests that mitochondrial translation activity is higher in Alstonville larvae (S2B Table, S7 Table). Further, we note that five genes associated with mitochondrial fusion [131] were also upregulated (*Optic atrophy 1* (*Opa1*), *HTRA2-related serine protease*, *rhomboid-7* (*eho*), *Heat shock protein cognate 5* (*Hsc70-5*), and *Myofilin* (*mf*); S2B Table) suggesting that mitochondrial morphology may differ between the mitotypes.

#### Metabolomics

GC/MS data detected 26 differentially abundant *Drosophila* metabolites between larvae with Alstonville and Dahomey mtDNA fed the two diets (Fig 7C & 7D and S5A & S5B Table). For the 1:2 P:C diet, three were higher in Alstonville and 16 elevated in Dahomey (Fig 7C and S5A Table). Multiple sugars were differentially abundant in Dahomey larvae on the 1:2 P:C diet suggesting the mtDNA encoded complex I mutation negatively impacted the flow of these metabolites through the electron transport system. The most abundant metabolite in Alstonville was succinate (Fig 7C). Succinate has multiple biological roles as a metabolic intermediate, is converted to fumarate by succinate dehydrogenase in complex II of the electron transport system and is a signalling molecule reflecting the cellular metabolic state [132]. Succinate occurred in higher levels in Dahomey than Alstonville fed the 1:16 P:C diet (Fig 7C and S5B Table).

For the 1:16 P:C diet, 12 metabolites were higher in Dahomey and three elevated in Alstonville larvae (Fig 7D and S5B Table). Lactate, alanine, glutaric acid, stearic acid, palmitic acid and D-mannose were higher in Dahomey on both foods. The high levels of lactate and alanine in Dahomey on both diets further supports the hypothesis that the complex I mutation is slightly deleterious in this mitotype. Lactic acidosis resulting from impaired utilisation of pyruvate is a hallmark of complex I dysfunction [133] and of mitochondrial disease in general [134]. The blood concentration of alanine is increased in most patients with electron transport system disorders [135].

#### Summary of Study 5

Linking transcriptomic and metabolomic data sets has been used to provide new insights into the interplay between genetics and environmental factors in a range of systems including the glycolytic state of *Drosophila* larvae [136], larval physiology [137, 138], physiological effects of high fat diet [139], and cold tolerance [140]. Here, transcriptomics and metabolomics data provided evidence that Alstonville has an advantage on the 1:2 P:C diet because Dahomey larvae are oxidatively stressed and the flow of sugar through the electron transport system is negatively impacted. In comparison, metabolism is extensively remodelled in both mitotypes on the 1:16 P:C diet.

Three lines of evidence strongly suggest that differential rates of glycolysis [141] do not cause the observed differences in development of the two mitotypes on the 1:16 P:C diet. First, we added the glycolytic inhibitor 2-Deoxy-D-glucose (2DG) to the diet and assayed numbers of flies eclosing in a 3 d window. Addition of 2DG to the 1:2 and 1:16 P:C diets did not affect the overall trend in fly development. Similar to the control diet, Alstonville developed 41% faster than Dahomey (t_8_ = 5.60, p = 0.001) when fed the 1:2 P:C diet but Dahomey developed 24% faster than Alstonville (t_8_ = 4.18, p = 0.003) when fed the 1:16 P:C diet. Second, we experimentally assayed the expression of *Glycogen phosphorylase* (*GlyP*) and the *estrogen-related receptor* (*ERR*). *GlyP* limits glycolysis flux capacity in adult *Drosophila* [142], while *ERR* has been proposed to regulate glycolytic gene expression in larvae [136]. Consistent with the transcriptomic analyses, expression of *GlyP* and *ERR* did not differ between the mitotypes (S6 Table). Third, we mined the transcriptomics data. Four genes involved in glycolysis were found to be differently expressed in the transcriptomic data (*6-phosphofructo-2-kinase* (*Pfrx*), *Hexokinase* C (*Hex-C*), *Succinyl coenzyme A synthetase α subunit* (*Scsalpha*) and CG7069, which is reported to have pyruvate kinase activity (S2 Table)). All four were significantly upregulated in Alstonville and therefore does not suggest that differential rates of glycolysis provided ATP for Dahomey. Further, analyses of the transcriptomics data showed the gene ontology biological process “ATP metabolic process” (GO:0046034) was down-regulated in Dahomey (S4D Table). Next, we compare the influence of the 1:2 P:C and the 1:16 P:C diets on levels of ROS production, oxidative stress, mtDNA copy number and ATP levels.

### Study 6: Response of *Drosophila* larvae to the 1:2 P:C and 1:16 P:C diets

In this section, we explore the disadvantage to Dahomey on the 1:2 P:C diet and begin to investigate the mitohormetic responses of the mitotypes fed the 1:16 P:C diet. We assay basal ROS production and expression of two Glutathione S-transferase (GST) genes because SOD activity was higher in Dahomey than Alstonville larvae and higher in mitotypes fed the 1:2 P:C diet (Fig 5C). Basal mitochondrial ROS gives the levels produced at the resting state and are an indicator of mitochondrial coupling efficiency in respiration [143]. ROS production and detoxification are tightly balanced, and numerous stress response mechanisms have evolved [144]. GSTs are a large supergene family of an ancient detoxifying enzyme and respond to endogenous and exogenous substrates through glutathione conjugation [145]. Transcriptomic data showed that Dahomey larvae fed the 1:2 P:C diet exhibited an elevation in cytochrome P450 metabolism (Fig 7A), and had higher expression levels of *GstE1* and *GstE5* (S2A Table). Here, we perform quantitative reverse transcription PCR (RT-qPCR) to confirm that the genes that were identified in the original RNA-seq were also altered as expected.

Next, mtDNA copy number and levels of ATP were assayed. Copy number is regulated by ROS in yeast [146], is positively linked to levels of ATP [147], and is crucial for maintaining cellular energy supplies [147, 148]. In *Drosophila*, mtDNA copy number is proposed to impact the organismal phenotype by influencing the respiratory membrane and the efficiency of oxidative phosphorylation [86]. ATP production has been shown to influence many cellular processes and evolutionary important physiological parameters including development rates [29, 149].

#### Basal ROS levels and oxidative stress

**Fig 8:**
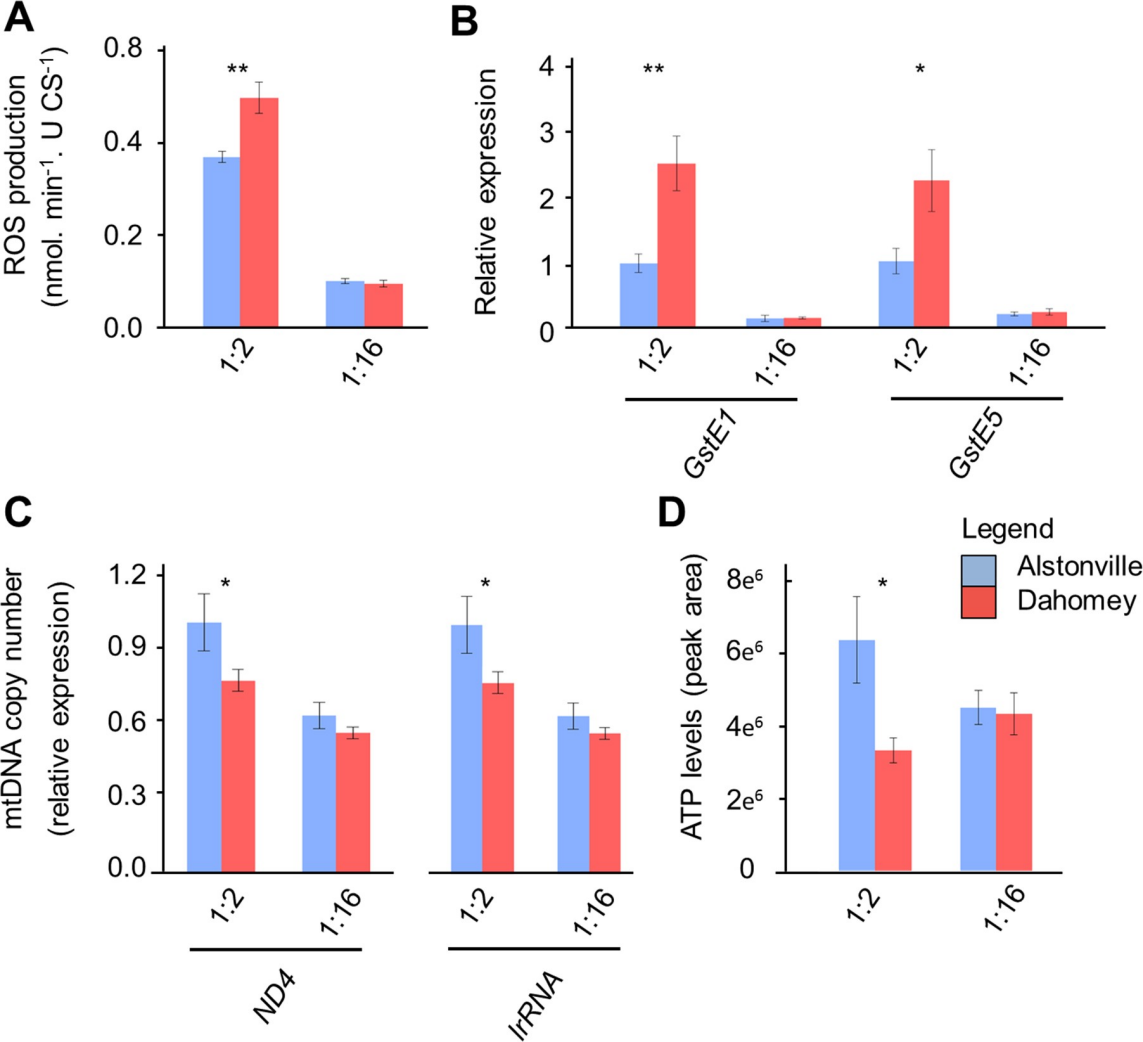
Basal ROS, antioxidant expression, mtDNA copy number and ATP levels Alstonville larvae had an advantage when fed the 1:2 P:C diet as the V161L ND4 amino acid change in complex I of Dahomey reduced the efficiency of ATP production. (**A**) Measurement of basal ROS shows higher levels in Dahomey fed the 1:2 P:C diet. ROS levels were similar when larvae were fed the 1:16 P:C diet (n = 9 biological rep/mitotype on the 1:2 P:C diet, and 8 biological rep/mitotype on the 1:16 P:C diet with 2 failed reactions in Alstonville). **(B**). *GstE1* and *GstE5* expression was highest in Dahomey larvae fed the 1:2 P:C diet (n = 6 biological rep/mitotype/diet for both genes). (**C)** Alstonville larvae had higher mtDNA copy number when fed the 1:2 P:C diet but both mitotypes had equivalent and lower copy number when fed the 1:16 P:C diet. MtDNA copy number show the relative expression of ND4 (ND4/Actin) and lrRNA (lrRNA/Rp49) (n = 8 biological rep/mitotype/diet with 2 failed reactions for ND4/Actin and 7 biological reps/mitotype/diet for lrRNA/Rp49 with 2 failed reactions). **(D)** Total cellular ATP levels were higher in Alstonville larvae fed the 1:2 P:C diet but were similar when fed the 1:16 P:C diet suggesting a mitohormetic response (n = 8 biological rep/mitotype/diet, with two failed reactions) Bars show mean± s.e.m. t-tests between mitotypes * p< 0.05, ** p< 0.01 (see text).

As predicted from the SOD and the transcriptomic data, basal ROS levels were higher (~35%) in Dahomey than Alstonville when both were fed the 1:2 P:C diet, but levels did not obviously differ between mitotypes fed the 1:16 P:C diet (Fig 8A). ANOVA showed significant main effects of mitotype, diet and their interaction (F_1, 28_ = 34.65, p< 0.0001, F_1, 28_ = 201.99, p< 0.0001, F_1, 28_ = 36.64, p< 0.0001, respectively). t-tests showed a significant difference between the mitotypes fed the 1:2 P:C diet (t_16_ = 6.93, p< 0.0001) but not between mitotypes fed the 1:16 P:C diet (t_12_ = 0.52, p = 0.61). The same trend was observed when superoxide levels were assayed (S4D Fig).

RT-qPCR corroborated the transcriptomic data that Dahomey larvae fed the 1:2 P:C diet were oxidatively stressed. For *GstE1*, Dahomey larvae had 156% greater expression than did Alstonville larvae when fed the 1:2 P:C diet (Fig 8B). There was no obvious difference in expression when larvae were fed the 1:16 P:C diet (S2B Table). Expression of *GstE1* showed significant effects of mitotype, diet, and their interaction (F_1,20_ = 12.00, p = 0.02, F_1,20_ = 29.73, p< 0.0001, F_1,20_ = 5.38, p = 0.03 respectively). t-tests showed a significant difference between mitotypes when fed the 1:2 P:C diet (t_10_ = 3.47, p = 0.006) but not when mitotypes were fed the 1:16 P:C diet (t_10_ = 0.14, p = 0.89). *GstE5* was upregulated ~121% in Dahomey on the 1:2 P:C diet and ~15% on the 1:16 P:C diet (Fig 8B). ANOVA showed significant effects of mitotype, diet, and their interaction (F_1,20_ = 6.04, p = 0.02, F_1,20_ = 29.73, p< 0.0001, F_1,20_ = 5.38, p = 0.03 respectively). t-tests showed a significant difference between the mitotypes when fed the 1:2 P:C diet (t_10_ = 2.41, p = 0.04) but not when the mitotypes were fed the 1:16 P:C diet (t_10_ = 0.06, p = 0.56).

#### MtDNA copy number and steady-state ATP levels

MtDNA copy number, assayed with independent primer sets, shows mitotype specific differences between larvae fed the 1:2 P:C diet, but not the 1:16 P:C diet. Overall, Alstonville had ~30% higher copy number on the 1:2 P:C diet and <10% higher copy number on the 1:16 P:C diet (Fig 8C). Comparing diets, mtDNA copy number was 52% higher on the 1:2 P:C than the 1:16 P:C diet. In regards to the ND4/actin set, mitotype and diet but not their interaction significantly affected copy number (F_1_, _26_ = 7.45, p = 0.01; F_1_, _26_ = 14.77, p = 0.0007 and F_1_, _26_ = 2.85, p = 0.10, respectively). t-tests showed significant differences between the mitotypes when fed the 1:2 P:C diet (t_13_ = 2.45, p = 0.03) but not when the mitotypes were fed the 1:16 P:C diet (t_13_ = 1.26, p = 0.23). Copy number with regards to the lrRNA/RP49 set showed a similar result (F_1_, _22_ = 5.33, p = 0.03; F_1_, _22_ = 30.41, p< 0.0001 and F_1_, _22_ = 2.89, p = 0.10, for mitotype, diet and their interaction, respectively). Again, t-tests showed significant differences between the mitotypes when fed the 1:2 P:C diet (t_10_ = 4.00, p = 0.003) but not when the mitotypes were fed the 1:16 P:C diet (t_10_ = 0.37, p = 0.72).

ATP levels support the hypothesis that metabolism is extensively remodelled in Dahomey larvae fed the 1:16 P:C diet (Fig 8D). ATP levels showed no significant effects of mitotype and diet but their interaction was significant (F_1_, _22_ = 1.74, p = 0.20; F_1_, _22_ = 1.79, p = 0.20 and F_1_, _22_ = 9.14, p = 0.01, respectively). t-tests showed significant differences between the mitotypes when fed the 1:2 P:C diet (t_11_ = 2.44, p = 0.03), but not when fed the 1:16 P:C diet (t_11_ = 0.19, p = 0.09).

#### Summary of Study 6

**Fig 9:**
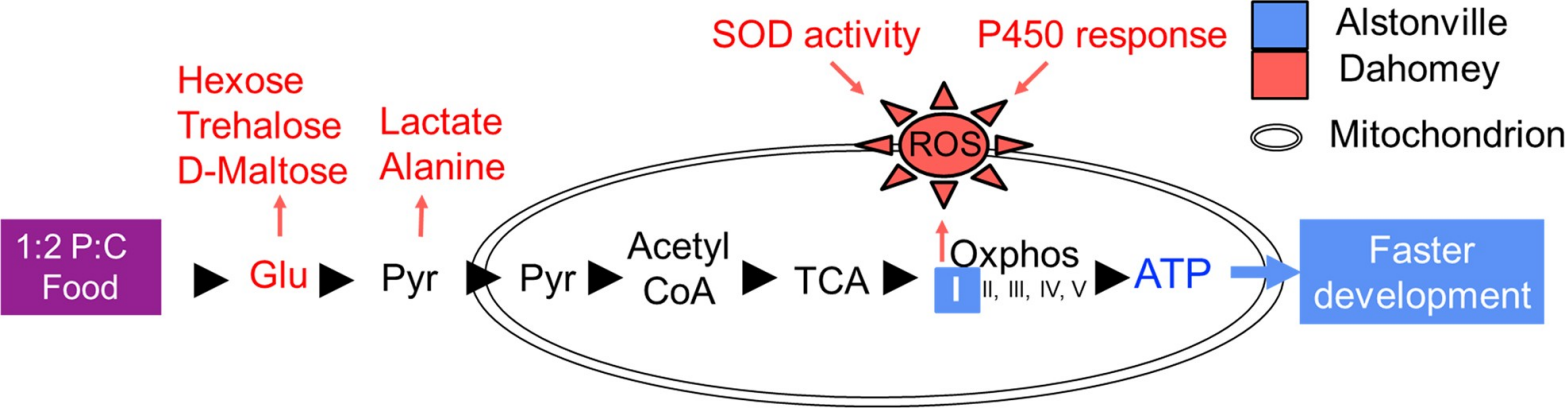
Proposed metabolic differences between *Drosophila* larvae fed the 1:2 P:C food. Development time for Alstonville larvae was faster than Dahomey because the V161L ND4 mutation in Dahomey caused reduced flow through the electron transport system. Red indicates elevated in Dahomey, blue higher in Alstonville. The mutation created a backup of glucose, which was likely metabolised to hexose, trehalose, and D-maltose. Lactate and alanine were also elevated. The mutation also caused an increase in ROS production, which resulted in an oxidative stress P450 response (including elevated levels of *GstE1* and *GstE5*), high SOD activity and a decrease in ATP level.

When fed the 1:2 P:C diet, the complex I mutation in Dahomey increased ROS production and oxidative stress and decreased mtDNA copy number and ATP levels (Fig 8, Fig 9). Consistent with this result, the rate of maximum ROS production increased more rapidly with age in Dahomey than Alstonville adult males when fed *Drosophila* instant food [62]. In adult females harbouring these mitotypes, Camus et al. [150] found the same rank order of mtDNA copy number when flies were fed an unknown diet. Our findings, however, conflict with the previous result that high mtDNA copy number correlates with longer development times [86]. One potential explanation for this difference is that we quantified copy number in third instar larvae while Salminen et al. [86] assayed adult virgins and pupae. A second experimental difference between the studies is the larval diets.

When fed the 1:16 P:C food, SOD activity was and superoxide levels were higher in Dahomey (Fig 5C, S4D Fig), but there were no significant differences in basal ROS production, *GstE1* and *GstE5* expression, mtDNA copy number or ATP levels (Fig 8). Future studies should assay the three major forms of SOD. The finding that ATP levels were similar but development rates differed predicts that either ATP does not constrain development or the mitotypes differentially allocate it. To explore these alternatives, we focus on the influence of the 1:16 P:C diet in the two mitotypes in Studies 7 and 8. In these studies we chose to manipulate the dietary sugars and partially block pathways in the mutant because the system is highly sensitive. We then assay development rate as a functional output that is directly related to the critical variable driving the population cage data. Again, we performed RT-qPCR to confirm that the genes that were identified in the original RNA-seq were also altered as expected. We start with the responses in Dahomey larvae, as Studies 1 and 2 showed the Dahomey mitotype increased in frequency when fed the 1:16 P:C diet.

### Study 7: Mitohormetic responses in Dahomey larvae fed 1:16 P:C food

We posited that the polyol pathway was mechanistically involved in the mitohometic response in Dahomey larvae due to the elevation of sorbitol levels in the metabolomics data and predicted that including dietary sugars in the pathway would be beneficial. If true, we hypothesised that the addition of the polyol pathway inhibitor Epalrestat would mitigate the net benefit. We then assayed the number of flies eclosing in 3 d, quantified the expression of *Notch* (*N*) and *Cyclic-AMP response element binding protein B* (*CrebB)*, and determined food consumption. In the non-disease context the polyol pathway is essential for cellular osmoregulation but, in the context of diabetes, it is associated with tissue-damage during hyperglycaemia [151]. In the pathway, glucose is reduced to sorbitol, via the action of the enzyme aldose reductase, and then oxidized to fructose. *O*-fucose and *O*-glucose are essential for normal Notch signalling [152] and their levels are regulated by derivatives of the polyol pathway including fructose, sorbitol and mannose, while xylose negatively regulates signalling [153, 154]. Notch regulates the cAMP responsive element binding protein (CREB) [155, 156], and experientially blocking CREB activity in *Drosophila* fat body has been shown to increase food intake [157]. *N* and *CrebB* were differentially expressed in the transcriptomics data (S2B Table).

The polyol pathway does not produce ATP so could not adequately account for the similarities in ATP levels between the mitotypes fed the 1:16 P:C diet. Here, we test the hypothesis that rates of β-oxidation differed between the mitotypes and add Etomoxir to the diet. β-oxidation of fatty acids generates NADH and FADH_2_ and thereby partially bypasses complex I of the electron transport system [91]. Etomoxir inhibits entry of long-chain fatty acids into the mitochondrion via the carnitine shuttle and we predicted its addition would result in loss of the selective advantage to Dahomey. We then quantified development, triglyceride levels, expression of *elongase F (eloF) and brummer* (*bmm*), β-oxidation activity, acetyl-coA enzyme activity, NAD^+^/NADH ratio and starvation survival. Metabolomic data showed high levels of stearic and palmitic acid in Dahomey larvae so we assayed triglycerides. To test for increased lipogenesis, we assayed the expression of *eloF* and *bmm*. *elofF* is a female-biased elongase involved in long-chain hydrocarbon biosynthesis [158]. *bmm* is a lipase which promotes fat mobilisation and is responsible for channelling fatty acids toward β-oxidation [159]. Both, *elofF* and *bmm* were differentially expressed in the transcriptomics data (S2B Table). β-oxidation was directly quantified using ^14^C-labelled palmitic acid. Acetyl-CoA was measured because the breakdown of carbohydrate influences its levels. NAD^+^ is required for fatty acid metabolism and the NAD^+^/NADH ratio was assayed. Starvation resistance was tested as a significant organismal trait [160]. When a larva is not feeding, energy can only come from the metabolism of existing resources [161], which occurs when fruits are small, when food quality declines and also in a fluctuating environment [162].

#### Upregulation of the polyol pathway in Dahomey

The polyol pathway converts glucose to fructose, however, fructose was not recorded as differentially present in Study 5 because glucose interferes with its detection in GC/MS [163]. Here, we assayed fructose with an enzymatic kit. As predicted from an increase in the activity of the polyol pathway, Dahomey larvae had significantly elevated levels of fructose (t_30_ = 3.40, p = 0.002).

**Fig 10:**
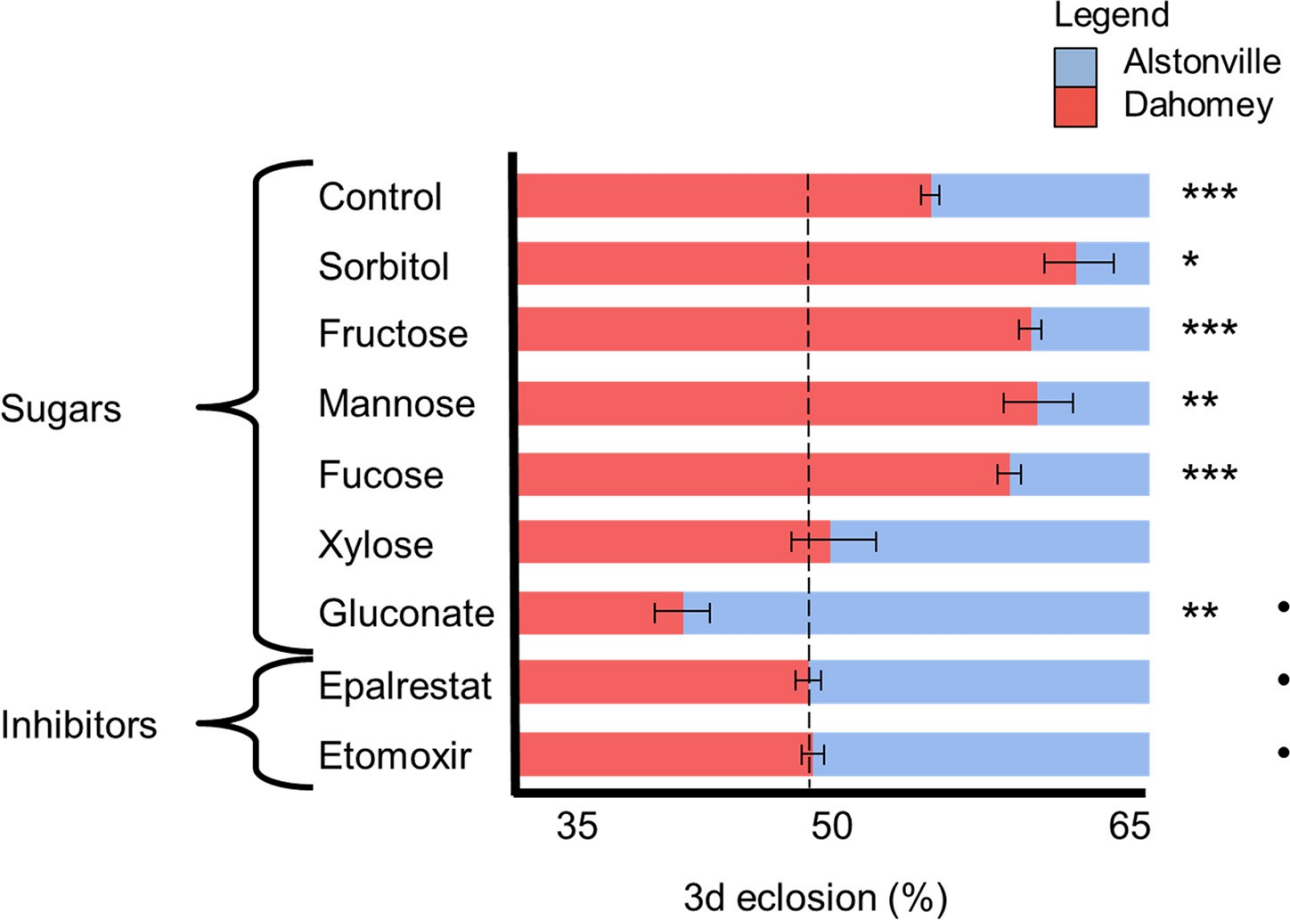
Tests of hypotheses using other sugars and inhibitors. Dietary modification of the 1:16 P:C diet with replacement of sugars (sucrose was the dietary sugar for the Control) and inhibitors. Replacement sugars were sorbitol, fructose, mannose, fucose, xylose, and gluconate (n = 4 rep/mitotype).The inhibitors were Epalrestat (Polyol pathway) (n = 5 rep/mitotype), and Etomoxir (β -oxidation) (n = 5 rep/mitotype). More Dahomey than Alstonville flies eclosed in a 3 d window when fed the control diet, as well as diets containing sorbitol, fructose, mannose, and fucose. Fewer Dahomey flies eclosed in a 3 d window when fed gluconate. There was no difference in the number of flies eclosing in 3 days between mitotypes when xylose was the dietary sugar or when Epalrestat or Etomoxir was added to the diet. * p< 0.05; ** p< 0.01, *** p<0.001 as determined by t-tests (see text). Dunnett’s tests compared Dahomey females fed the control diet to diets supplemented with inhibitors or the control diet compared with other sugars • p< 0.05 (see text).

Our predictions that including dietary sugars in the polyol pathway would be beneficial while addition of the inhibitor Epalrestat would mitigate the net benefit to Dahomey larvae were observed. Replacing sucrose (control) with sorbitol, fructose, mannose, and fucose caused more Dahomey than Alstonville to eclose in a 3 d window (t_6_ = 2.64, p = 0.038, t_6_ = 11.7, p< 0.0001, t_6_ = 4.42, p< 0.005, t_6_ = 11.60, p< 0.0001 for sorbitol, fructose, mannose and fucose, respectively; Fig 10). Differences in development time were lost when xylose was the dietary sugar (t_6_ = 1.23, p = 0.27; Fig 10). Dunnett’s test demonstrated that similar numbers of Dahomey females eclosed in 3 d when fed the control diet and when sucrose was replaced with sorbitol, fructose, mannose, fucose and xylose (*Q* = 2.80, p>0.05 in all cases; Fig 10). Addition of the inhibitor Epalrestat to the diet caused the repeatable differences in numbers of flies eclosing in 3 d to be lost (t_8_ = 0.475, p = 0.647; Fig 10). Dunnett’s test showed a significant difference between numbers of Dahomey larvae eclosing in 3 d when fed the control diet and the diet supplemented with the inhibitor (*Q* = 2.59, p = 0.001; Fig 10).

**Fig 11:**
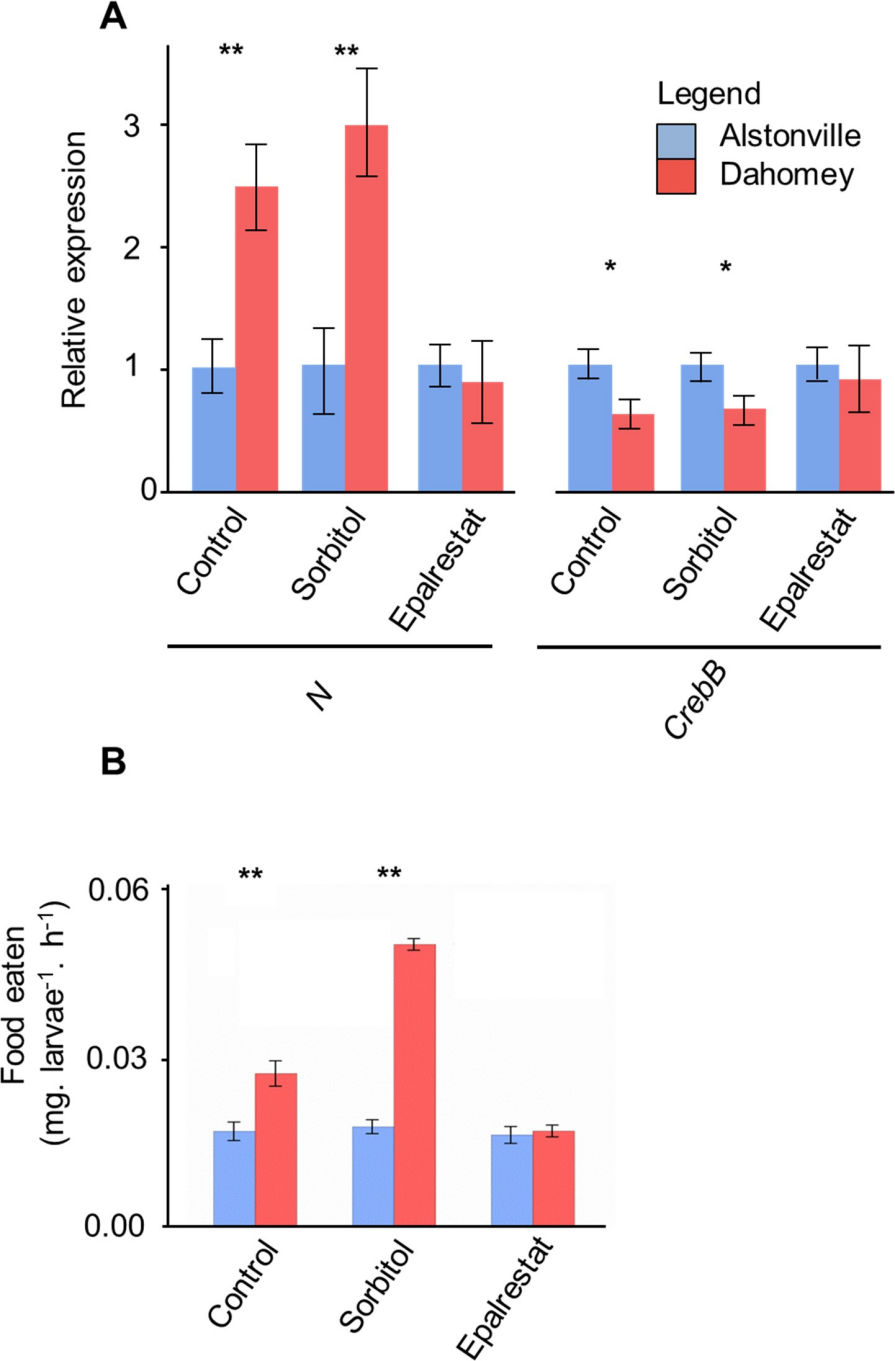
The polyol pathway is upregulated in Dahomey larvae fed the 1:16 P:C diet. (**A**) Expression of *N* and *CrebB* differed when larvae were fed the control (sucrose) diet, or sorbitol was the dietary sugar, but differences were lost when Epalrestat was added to the diet (n = 6 rep/mitotype, with n = 8 for Epalrestat fed Dahomey). (**B**) Food eaten was higher in Dahomey larvae than in Alstonville larvae. Food consumption increased when sorbitol was the dietary sugar and decreased when Epalrestat was added to the diet (n = 12 larvae/mitotype/diet were added to dye labelled food. Larvae with food visible in guts were collected and analysed: control-Alstonville = 7 larvae, control-Dahomey = 9 larvae, sorbitol-Alstonville = 8 larvae, sorbitol-Dahomey = 11 larvae, Epalrestat-both mitotypes = 10 larvae. Bars show mean ± s.e.m. * p< 0.05 and ** p< 0.01, as calculated by t-tests (see text).

As expected, increasing activity of the polyol pathway with the dietary addition of sorbitol increased *N* and decreased *CrebB* expression. Blocking the pathway with Epalrestat had the opposite effect. *N* expression increased 18% in Dahomey when the dietary sugar was sorbitol and decreased to the same level as Alstonville when Epalrestat was added (t_10_ = 4.36, p = 0.001, t_10_ = 3.90, p = 0.003, t_12_ = 0.06, p = 0.95 for control, sorbitol and Epalrestat, respectively; Fig 11A, S6B Table). *CrebB* expression was <5% higher in Dahomey larvae fed sorbitol compared to sucrose, but increased by 41% with the addition of Epalrestat (t_10_ = 2.23, p = 0.046; t_10_ = 2.72, p = 0.022, t_12_ = 0.38, p = 0.711 for sucrose, sorbitol and Epalrestat, respectively; Fig 11A, S6B Table).

For food consumption, larvae with Dahomey mtDNA ate 57% more when sucrose was the dietary sugar and 156% more when sorbitol was the dietary sugar (t_14_ = 4.58, p = 0.0004, t_17_ = 23.31, p< 0.0001; Fig 11B). This distinct difference in food consumption was lost when Epalrestat was added to the diet (t_18_ = 0.48, p = 0.64; Fig 11B). Therefore, we conclude that increased activity of the polyol pathway resulted in increased food consumption.

#### Increased β-oxidation of fatty acids in Dahomey

**Fig 12:**
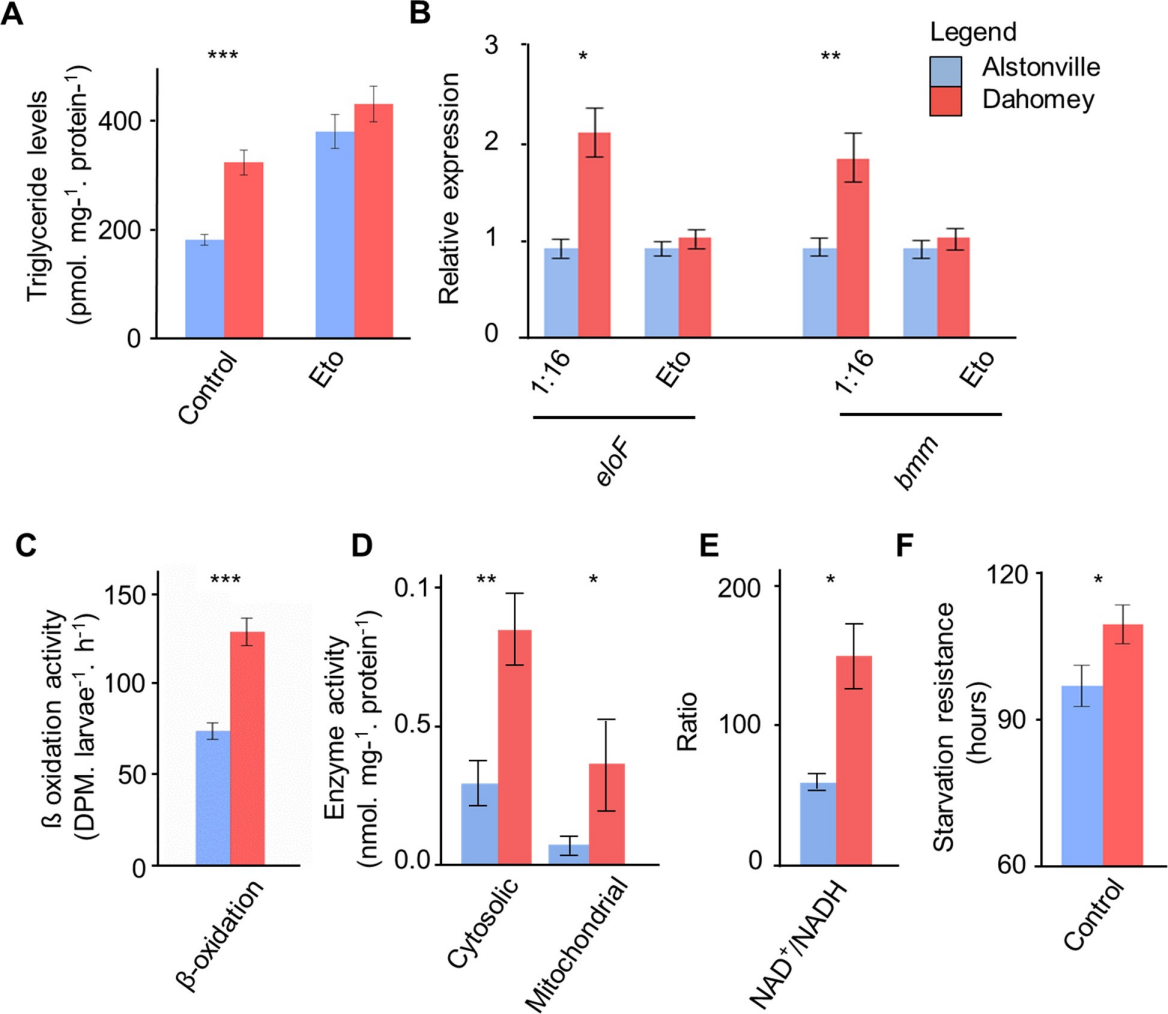
β-oxidation of fatty acids is upregulated in Dahomey larvae fed the 1:16 P:C diet. (**A**) Triglyceride levels were higher in Dahomey larvae fed the control diet. When Etomoxir (Eto) was added to the control diet, triglyceride levels increased, and differences between the mitotypes was lost (n = 14 rep/mitotype/treatment with 6 failed reactions). (**B**) Expression of *eloF*) and *bmm)* were higher in Dahomey larvae fed the control diet. Differences were lost when Etomoxir (Eto) was added to the control diet (n = 6 rep/mitotype). (**C**) β-oxidation activity was highest in Dahomey larvae (n = 10 biological rep/mitotype–with one outlier removed). (**D**) Acetyl-coA enzyme activity in the cytosol and extracted mitochondria was higher in Dahomey larvae. (n = 9 biological rep/mitotype–with four outliers removed from the cytosol data). (**E**). NAD^+^/NADH ratio was higher in Dahomey larvae (n = 7 rep/mitotype). (**F**) Starvation survival was greatest in Dahomey larvae (n = 56 for Alstonville and 91 for Dahomey). Bars (mean ± s.e.m). p< 0.05 and p< 0.01, as calculated by t-tests (see text).

β-oxidation activity was highest in Dahomey larvae and this enabled the partial bypass of the complex I mutation. As predicted, the dietary addition of Etomoxir removed Dahomey’s advantage. For development, Etomoxir caused the repeatable differences in numbers of flies eclosing in 3 d to be lost (t_8_ = 0.10, p = 0.92; Fig 10). There was a significant difference between numbers of Dahomey eclosing in 3 d when fed the control diet and the food supplemented with inhibitor (*Q* = 2.59, p< 0.02; Fig 10). For triglycerides, levels were 76% higher in Dahomey when fed the control diet, but this clear difference was lost when Etomoxir was added to the diet (t_25_ = 5.52, p< 0.0001, t_21_ = 1.07, p = 0.30, for control and Etomoxir, respectively; Fig 12A).

Lipogenesis was higher in Dahomey larvae. There was a 140% higher expression of *eloF* in Dahomey larvae fed the control diet (t_10_ = 3.02, p = 0.013; Fig 12B, S6B Table), but no difference when Etomoxir was added to the diet (t_10_ = 0.26, p = 0.803; Fig 12B). For *bmm*, Dahomey larvae showed 100% higher expression on the control 1:16 P:C diet (t_10_ = 3.65, p = 0.004; Fig 12B, S6B Table). Again, this difference was lost when Etomoxir was added to the diet (t_9_ = 0.51, p = 0.62; Fig 12B).

Increased rates of β-oxidation in Dahomey are consistent with the higher ATP levels (Fig 8D) on the 1:16 P:C food than the 1:2 P:C diet. The rate of β-oxidation in Dahomey larvae was almost double that observed in Alstonville larvae (t_17_ = 5.98, p< 0.0001; Fig 12C). Further, the levels of cytosolic and mitochondrial acetyl-CoA were also markedly elevated being 181% and 584% higher, respectively (t_12_ = 3.87, p = 0.002, t_16_ = 8.84, p< 0.0001, for cytosolic and mitochondrial, respectively Fig 12D). The NAD^+^/NADH ratio in Dahomey larvae was 131% higher than that observed in Alstonville suggesting that the rate of β-oxidation was not limited by NAD^+^ availability (t_12_ = 2.79, p = 0.016; Fig 12E).

As expected, starvation resistance was higher in Dahomey than Alstonville larvae (t_145_ = 2.82, p = 0.005; Fig 12F). Likely, starved larvae released lipids from the fat body and lipid droplets accumulated in oenocytes [161]. Oenocytes are secretory cells that express an extensive battery of lipid-synthesizing and -catabolizing enzymes including fatty acid elongases and fatty acid β-oxidation enzymes [164]. Plausibly, this could provide an advantage to Dahomey larvae in nature.

#### Summary of Study 7

**Fig 13:**
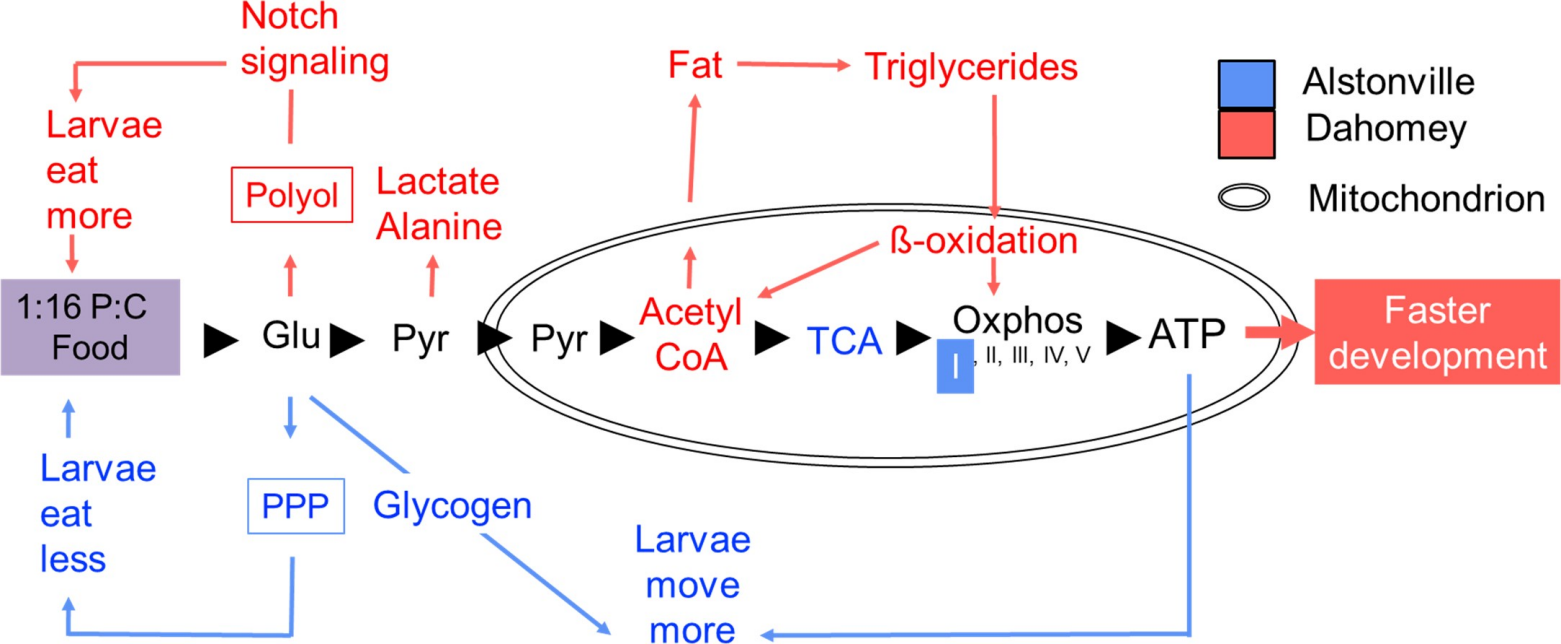
Proposed mitohormetic responses in *Drosophila* larvae fed the 1:16 P:C food (red indicates elevated in Dahomey, blue higher in Alstonville). The mitohormetic response, involving at least two separate pathways, enabled Dahomey to develop faster than Alstonville larvae. First, larvae with Dahomey mtDNA ate more, which caused third instar larvae to weigh more. Backup of sugars produced increased activity of the polyol pathway and increased *N* expression. Increased *N* expression blocked *CrebB* and fed back to increase food consumption. Second, pyruvate was metabolised to acetyl-CoA and exported from the mitochondrion for fatty acid synthesis and palmitic acid and stearic acid levels increased. The long-chain fatty acids were catabolised by β oxidation, resulting in the formation of NADH and FADH_2_. FADH_2_ shuttled electrons to the quinone pool and partially by-passed ETC complex I where the V161L mutation occurred. In contrast, Alstonville larvae upregulated glycogen metabolism and activity of the pentose phosphate pathway increased. Increased glycogen metabolism increased wandering, which diverted energy away from development. Increased insulin signalling decreased larval food consumption.

We posit that mitohormetic responses resulted in higher levels of ATP in Dahomey larvae fed the 1:16 P:C diet as compared to those raised on the 1:2 P:C food (Fig 13). Here, we have shown increased activity of the polyol pathway, corroborated differences in the expression of *N* and *CrebB* and revealed increased food consumption. We used an activator (sorbitol) and an inhibitor (Epalrestat) of this pathway and observed the expected gene expression changes with RT-qPCR and the expected changes to feeding behaviour.

The increased food consumption in Dahomey, fuelled an increase in lipogenesis and fat storage, which caused larvae to weigh more (Fig 5D) and develop more quickly than Alstonville larvae (Fig 13). Increased β-oxidation of fatty acids generated acetyl-coA, NADH, and FADH_2_, which partially bypassed complex I harbouring the V161L ND4 mutation. As expected, blocking long-chain fatty acid entry into the mitochondrion via the carnitine shuttle with Etomoxir caused the transcriptomic and RT-qPCR differences in *eloF* and *bmm* expression to be lost (Fig 12B), resulting in loss of the selective advantage (Fig 10).

The mitohormesis response in Dahomey is diet dependent and not dependent on the LKB1-SIK3 pathway or differential expression of *Sirtuin 2* (*Sirt2*). To explicitly test whether metabolic rewiring in these mitotypes was fixed or dependent on the diet, we assayed *eloF* and *bmm* expression on the 1:2 P:C diet. Expression levels were not statistically different supporting the hypothesis that the rewiring was diet dependent (t_10_ = 0.85, p = 0.42 and t_10_ = 1.00, p = 0.34 for *eloF* and *bmm*, respectively). To investigate the potential for the LKB1-SIK3 pathway to regulate lipid metabolism [165] we mined the transcriptomic data. The RNA-seq data did not show differential expression of the serine/threonine kinase *Lkb1*, *Salt inducible kinase 3* (*Sik3*) or *CREB-regulated transcription coactivator (Crtc)* (S2 Table). We did note, however, that *Salt inducible kinase 2* (*Sik2*) was significantly overexpressed in Dahomey larvae (S2B Table). *Sik2* is reported to have an important role in nutrient-dependent signalling homeostasis and to be a negative regulator of the conserved Hippo pathway [166]. We also checked for the differential expression *Sirt2*, as a buoyancy-based screen of *Drosophila* larvae revealed that it played a role in coupling fat storage to nutrient availability [167]. Transcriptomic data did not suggest that *Sirt2* was differentially expressed between mitotypes (S2 Table).

Overall, these data show that multiple pathways are involved in the Dahomey mitohormetic response. One potential explanation for the mitotype specific differences in development is disparities in fat metabolism. Triglyceride deposition increases throughout the larval stage in *Drosophila* before reducing three-fold during metamorphosis [168]. Consequently, differences in triglyceride content, particularly within the fat body, may alter the antagonistic relationship between insulin and ecdysone and affect developmental timing [169]. In support of this hypothesis, development rates were similar between mitotypes when Etomoxir was added to the diet (Fig 10). Differences in fat metabolism do not, however, provide an overarching explanation for developmental differences on both diets. An alternative explanation may be differential Notch and/or FOXO signalling [127, 128]. Neither hypothesis, however, fully explains the previous result that mitotype specific differences in development are lost at 19° C when food consumption but not movement differed [15]. Next, we focus on the influence of the 1:16 P:C diet on mitochondrial metabolism in Alstonville.

### Study 8: Mitohormetic responses in Alstonville larvae fed 1:16 P:C food

Transcriptomic data discussed in Study 5 actively support the result that a general increase in mitochondrial gene expression is part of rewiring in Alstonville on the 1:16 P:C diet. Furthermore, we hypothesised that glucose-6-phosphate was differentially metabolized in Alstonville due to the observed elevation in gluconate. Glucose 6-phosphate can be converted to store glycogen through the action of glycogen synthase and so we assayed levels of glycogen. *Glycogen synthase* and *insulin-like receptor* (*Inr*) are elevated in Alstonville (S2 Table). Glycogen is a primary source of energy for adult muscle function [170, 171] and the ubiquitous activation of *Inr* has previously been shown to cause larvae to feed less and to wander off the food [172]. Therefore, we assay development time and movement.

Glucose 6-phosphate is also metabolized by the pentose phosphate pathway and D-Gluconate can be phosphorylated to 6-phospho-D-gluconate to enter the oxidative phase of the pathway [173]. Here we quantified the expression of *Zwischenferment* (*Zw*) and assayed glucose-6-phosphate dehydrogenase (G6PD) activity. *Zw* was differentially expressed in the transcriptomics data (S2B Table). *Zw* catalyses the oxidation of glucose-6-phosphate (G6P) to 6-phosphogluconate. G6PD is the rate-limiting enzyme of the pentose phosphate pathway [174, 175]. We then assayed one aspect of insulin signalling. The insulin/insulin-like growth factor signalling pathway controls a wide variety of biological processes in metazoans [176] and stimulates glucose metabolism via the pentose phosphate pathway in *Drosophila* cells [177]. The most upstream central players in this pathway are members of the insulin-like peptide (ILP) family, which includes insulin and insulin-like growth factors in mammals [178], as well as multiple ILPs in worms and insects [179]. ILPs are regulated by nutritional status and *Insulin-like peptide 2* (*Ilp2*) is essential for maintaining normoglycemia [180]. We assayed *Ilp2* to corroborate the results from the transcriptomics data (S2B Table). Here, we replaced sucrose (control) with gluconate as the dietary sugar, but did not include any blockers because we considered this the wild-type pathway on the 1:16 P:C diet.

#### Increased glycogen metabolism

**Fig 14:**
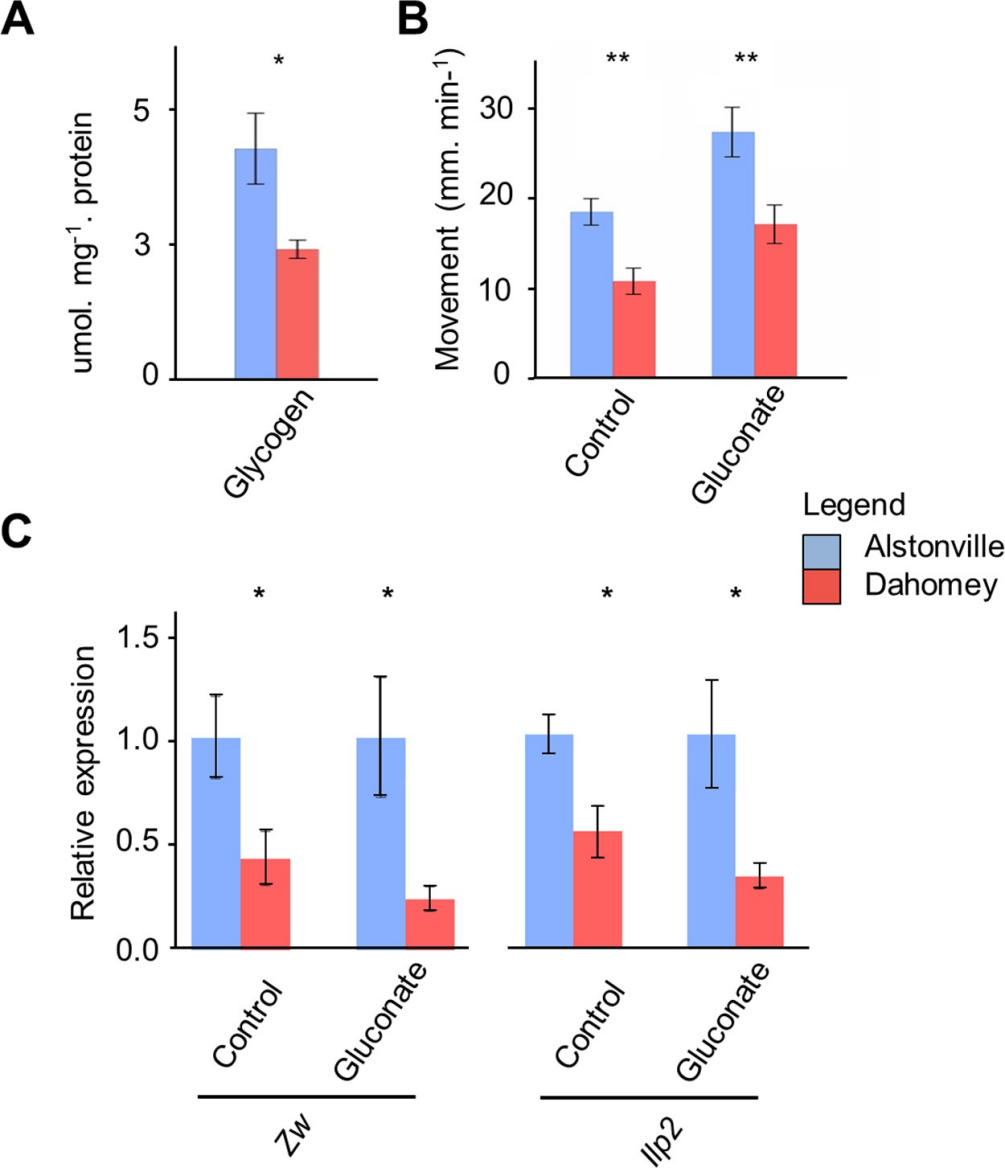
Glycogen metabolism is increased and the pentose phosphate pathway is upregulated in Alstonville larvae fed the 1:16 P:C diet. (**A**) Glycogen level was highest in Alstonville larvae (n = 10 biological rep/mitotype). (**B**) Physical activity was highest in Alstonville larvae fed the control (sucrose) diet and when gluconate was the dietary sugar (n = 16 larvae/mitotype with 3 outliers removed when fed sucrose and 12 larvae/mitotype when fed gluconate). (**C**) Expression of *Zw* and the *Ilp2* on control (sucrose) and gluconate diets were higher in Alstonville larvae (n = 6 rep/mitotype with 1 failed reaction). Bars (mean ± s.e.m). * p< 0.05 and ** p< 0.01, as calculated by t-tests (see text).

Glycogen is a multi-branched polysaccharide of glucose and is too large to detect by GC-MS. It serves as a form of energy storage in insects [181]. As predicted, glycogen levels were 72% higher in Alstonville than in Dahomey larvae (t_18_ = 2.59, p = 0.02; Fig 14A).

Dietary addition of gluconate delayed development in both mitotypes but the developmental delay was greater in Dahomey (Fig 10). Gluconate caused 54% more Alstonville than Dahomey to eclose in 3 d (t_6_ = 3.87, p = 0.008). Dunnett’s test demonstrated that more Dahomey females eclosed in 3 d when fed the control diet than when gluconate was the dietary sugar (*Q* = 2.59, p = 0.001; Fig 10).

Increasing gluconate levels increased larval physical activity. Alstonville larvae exhibited greater physical activity than Dahomey larvae and movement increased with the dietary addition of gluconate (Fig 14B). Physical activity was 72% greater in Alstonville larvae fed sucrose (t_27_ = 3.54, p = 0.002). When gluconate was the dietary sugar physical activity increased by 52% and remained higher in Alstonville (t_22_ = 2.81, p = 0.01; Fig 14B).

#### Upregulation of the pentose phosphate pathway in Alstonville

Alstonville had higher *Zw* expression than Dahomey and the relative difference in expression increased with the dietary addition of gluconate. Expression of *Zw* differed on the 1:16 P:C diet with Alstonville showing a 56% higher expression of *Zw* than Dahomey, while Alstonville larvae fed gluconate showed 75% higher expression than Dahomey (t_10_ = 2.46, p = 0.034, t_10_ = 2.66, p = 0.024, respectively; Fig 14C, S6B Table). Consistent with these results, G6PD activity was 109% higher in Alstonville larvae (t_14_ = 2.70, p = 0.02; S7 Fig).

Insulin signalling, suggested by increased *Ilp2* expression, was also higher in Alstonville. *Ilp2* expression was more than twice as high in Alstonville than in Dahomey larvae (t_9_ = 2.77, p = 0.02, t_10_ = 2.38, p = 0.04, for control and gluconate, respectively; Fig 14C, S6B Table). It has been convincingly argued that insulin signalling influences food consumption and locomotion in adults flies and feeding in *Drosophila* larvae [182, 183]. Future studies may consider including western blotting to assess insulin signalling.

#### Summary of Study 8

Alstonville fed the 1:16 P:C diet had elevated mitochondrial gene expression, produced more glycogen, were more active and had increased activity of the pentose phosphate pathway with greater insulin signalling. We postulate that the greater physical activity caused Alstonville larvae to redirect the resource away from development (Fig 13). Physical movement requires ATP [185] and is critical for dispersal behaviour seen upon nutrient deprivation [186]. The cost of increased physical activity is the diversion of energy away from cell growth and division resulting in slowed development [29, 149, 187]. Towarnicki and Ballard [15] raised larvae at 19° C, 23° C and 27° C and measured development time, food consumption and movement. At the two higher temperatures, Alstonville larvae developed more slowly, ate less and moved more. When larvae were raised at 19° C movement, and development time of these mitotypes did not differ, but differences in food consumption remained. Thus, temperature may have an indirect affect causing a reduction in movement of the poikilotherm. Further supporting this hypothesis, Wieser [188] presented evidence to suggest that the cost of growth decreases with rate of growth in ectotherms.

As observed in the transcriptomic data (S2B Table) *mitochondrial transcription factor A* (TFAM), a key regulator of mitochondrial gene expression, is not differentially expressed (t_10_ = 0.14, p = 0.89; S2B & S6B Tables). This is not surprising, as the levels of TFAM are known to be directly proportional to mtDNA copy number [184] and the latter is lower in Alstonville larvae on the 1:16 diet (Fig 8C). We conclude that the difference in mitochondrial gene expression is not due to TFAM.

Increased glycogen levels in Alstonville larvae predicted that we would see an elevation in HR38 [170], the single fly ortholog of the mammalian nuclear receptor 4A family of nuclear receptors. Mining of the transcriptomic database did not suggest that was the case (S2B Table). There was, however, evidence for increased expression of the *Drosophila p70/S6 kinase (S6K)*, which is reported to be a key organizer of hunger-driven feeding behaviours in *Drosophila* larvae [186].

### Conclusion

Over the past decade, it has become clear that diet is an evolutionary force that has immediate implications for our understanding of health and disease. Here, we provide substantial evidence to suggest that a single mtDNA encoded nonsynonymous mutation can differently influence the regulation of dietary metabolites and have significant phenotypic consequences. When fed the 1:2 P:C diet, Alstonville larvae had a relative advantage as the V161L ND4 mutation in Dahomey caused an increase in ROS production, which resulted in oxidative stress and a decrease in mitochondrial functions leading to reduced mtDNA copy number and ATP levels.

When fed the 1:16 P:C diet, Dahomey larvae had the relative advantage with multiple linked pathways working in a synergistic mitohormetic response that enabled larvae to eat more and develop more quickly. The remodelled pathways in Dahomey included upregulation of the polyol pathway, which fed back to increase food consumption and fuelled increased β-oxidation of fatty acids. Each cycle of β-oxidation results in the donation of electrons to the quinone pool downstream of complex I in the electron transport system, thereby bypassing the V161L, ND4 subunit mutation [189]. This process maintains levels of the quinone pool, which has been shown to be functionally important [190]. In Alstonville, mitochondrial gene expression was higher, glycogen metabolism increased and larvae were more active. We postulate that the greater physical movement in Alstonville larvae on the 1:16 P:C diet caused a reallocation of ATP away from cell division and growth, thereby slowing development. ATP drives many cellular processes and constrains development rates [29, 149]. An alternative explanation is that upregulation of Notch and/or FOXO signalling in Dahomey may be responsible for driving mitotype-specific differences in development [127, 128].

These data further question whether mtDNA can be assumed to accurately reflect species or population-level demographic processes when the dietary protein to carbohydrate ratio varies over time or space. It is now well documented that purifying selection affects the variability of mtDNA encoded genes, and the purging of deleterious variants will result in the removal of linked variants through background selection. In humans, deleterious mtDNA mutations are well-known [41–43], and evidence for a profound effect of accumulated mutations on men’s health has been reported [191]. Purifying selection has been demonstrated in the female mouse germline [39, 40] and in *Drosophila* slightly deleterious mutations have been reported [26, 36–38]. Evidence of positive selection on mitogenomes has been reported [27, 52], but to our knowledge, no specific mutation has been experimentally shown to result in an evolutionary advantage. Our observation that distinct mitotypes reached high frequency when fed different macronutrient ratios in population cages suggests that diet may also be a strong selective force in nature. Here, we advocate future studies test for selection on mtDNA within and among naturally occurring populations where macronutrients change over time and space.

The influence of diet is extensively studied in the literature but few studies investigate genotype-by-diet interactions and fewer still that have unravelled the underlying mechanisms [3–7, 51, 143, 192]. One prediction from these data is that experimentally increasing the P:C ratio (i.e., 1:20 P:C) may further increase the dietary-induced metabolic stress and cause increased mortality in larvae harbouring Dahomey mtDNA. Conversely, development time in Alstonville larvae may decrease if the polyol pathway is upregulated. Experimentally, such a dietary perturbation would be outside the range of P:C ratios encountered by *Drosophila* in nature, but perhaps would reflect the human genomes clash with modern life and the vending machine.

Most common mtDNA mutations are thought to be deleterious and involved in a variety mitochondriopathies and complex diseases like diabetes, cardiovascular disease, gastrointestinal disorders, skin disorders and elevated blood pressure [e.g. 193, 194–197]. Further, the accumulation of somatic mtDNA mutations likely influences primary cancers and the ageing process [198, 199]. The data presented here suggest that it is also possible that slightly deleterious mtDNA mutations may confer an advantage in certain situations. Our data, therefore, support matching an individual’s diet to their mitotype as an approach to treating mitochondriopathies, complex diseases or even for optimising health in non-disease populations. For example, were the same mechanisms found to occur in humans the enhanced lipogenesis in individuals with mild complex I mutations could make them more venerable to obesity when eating a high-carbohydrate diet, yet less susceptible to Parkinson’s disease, which has been linked to defects in lipogenesis [200].

## Materials and methods

### Study 1: Population cages, larval development, and fecundity of four mitotypes

#### Fly lines

Five *D*. *melanogaster* lines harbouring distinct mitotypes were initially collected in nature (Alstonville, Dahomey, Japan, Madang and Victoria Falls) and *w*^1118^ was a laboratory construct sourced from Bloomington Stock Center. Oregon R, Canton S and all balancer stocks were obtained from Bloomington.

For this study, isogenic lines were constructed by chromosome replacement using balancers and differed in their mitochondrial genomes and chromosome 4 [66]. Mitochondrial DNA encoded amino acid, tRNA, rRNA and A+T rich region differences in these fly lines were previously reported [66, 83, 84]. Since arrival in the lab, the Alstonville, Dahomey, Japan and *w*^1118^ mitotypes they have been subject to over 25 generations of backcrossing to *w*^1118^ thereby reducing chromosome 4 variation. Six generations of backcrossing occurred immediately before the commencement of the cage studies and additional backcrossing of each mitotype before each Study. To further reduce the potential for nuclear-encoded mutations to influence the results, three independently maintained lines were included in all physiological studies. The mitotype of all lines was checked every six months by Sanger sequencing [83]. Lines did not harbour *Wolbachia* infection or p-elements [83] and no evidence of heteroplasmy was detected. Samples in the study were randomised, but investigators were not blinded to the sample group.

#### Fly diets

Four isocaloric P:C diets were prepared by varying yeast, treacle, semolina and sucrose content in a standard base containing 1% agar, 0.1% nipagen, 0.1% propionic acid and 0.001% phosphoric acid. The P:C ratios were 1:2, 1:4, 1:8 and 1:16. The final concentration of ingredients was set at 180 g/L. The 1:2 P:C diet contained 79.4 g of yeast, 73.2 g of semolina, 27.2 g treacle and 0.2 g of sucrose per litre (so dietary sucrose comprised ~0.1% of the 1:2 P:C diet). The 1:4 P:C diet contained 46.5 g yeast, 42.4 g semolina, 89.16 g treacle and 1.4 g sucrose per litre. The 1:8 P:C diet contained 25.2 g yeast, 23.2 g semolina, 127.5 g treacle and 4.1 g sucrose per litre. The 1:16 P:C diet contained 12.9 g of yeast, 11.9 g of semolina, 145.9 g of treacle and 9.3 g sucrose per litre (so dietary sucrose comprised ~5% of the 1:16 P:C diet). The autolysed yeast (MP Biomedicals, catalogue no. 103304), contains 45% protein, 24% carbohydrate, 21% indigestible fibre, 8% water and 2% acids, minerals and vitamins. The treacle (CSR, Vic, Australia) contains 0.4% protein, 71% carbohydrate and 0.17% of sodium. The semolina (Quality Food Services, Qld, Australia) contains 11.8% protein, 68.8% carbohydrate, 1.6% fat, 3.2% dietary fibre and 0.0037% sodium. A limitation of these diets is that we could not modify the sugars while maintaining the P:C ratio and caloric content for the 1:2 P:C diets (protocol.io reference dx.doi.org/10.17504/protocols.io.r6xd9fn).

Unless otherwise stated for all studies, flies from each of the mitotypes were raised for at least two generations on instant *Drosophila* food (Carolina Biological Supply Company, NC, USA). Flies were placed in individual egg collection containers, and eggs manually added to either the 1:2 or 1:16 P:C diet with ~200 eggs per bottle. Microbiome was added after 48 hours. The microbiome was established by adding 130 μL of a homogenate from four males of each mitotype ground in 1.5 mL of distilled water.

#### Population cage studies including four mitotypes and four diets

Cage studies were started by placing ~210 eggs from each fly mitotype onto instant *Drosophila* food in bottles. Bottles, each with a different mitotype, were placed into population cages (22 cm x 21 cm x 36 cm) such that there were ~850 flies/cage. To establish a homogeneous gut flora each generation, four males from each mitotype were ground in 1.5 ml of distilled water and 130 μL of the homogenate containing gut microbes was aliquoted into each bottle. On the first day of eclosion (adult emerging from a pupal case), plugs were removed from bottles and flies were released into population cages for 3 d. Bottles were removed, and oviposition resources (yeast placed on top of the solidified agar-based medium containing 4% agar and 10% treacle) were put in cages and eggs were collected from 3–5 d old adult females. Surface sterilisation of eggs was achieved by washing in dilute bleach, and ~200 eggs were then placed on each diet. This protocol was then repeated for each generation. Following oviposition, adult flies were frozen. The frequency of adult females harbouring the distinct mitotypes was individually determined by sequencing and allele-specific PCR of 95 individual females from each cage. For the initial studies, DNA was extracted, and a ~900 bp region of mtDNA amplified using the ND4L forward 5’-TAAACAAACTAATCTAACTAATA-3’ and reverse 3’-GGTTGTGATATATTATCTTATGG-5’ primer and Sanger sequenced. Chromatograms were imported into Sequencher 4.5 (Gene Codes, MI, USA) and the proportion of each mitotype/diet/generation determined. DeLuca and O’Farrell [201] show that mature *D*. *melanogaster* sperm lack mtDNA. As a consequence, estimation of the frequencies of mitotypes in population cages is unlikely to be confounded by paternally derived mtDNA (dx.doi.org/10.17504/protocols.io.rqyd5xw).

#### Distinguishing immature and adult affects

To determine which life history stage drove the 4 *×* 4 population cage data, we first assayed immature (larval + pupal) development time and adult fitness. Flies that eclosed in a 3 d window were collected, counted and % eclosion was determined by dividing the number of eclosed flies from each mitotype by the sum of both mitotypes (dx.doi.org/10.17504/protocols.io.rqzd5x6).

Next, we determined whether the observed differences in the number of Alstonville and Dahomey flies that eclosed in 3 d resulted from differences in larval development, larval mortality or adult fecundity, we assayed time to pupation, numbers eclosing in a 6 d window and numbers of female offspring. Pupation was determined following Towarnicki & Ballard [15]. Briefly, 10 vials were established with 10 eggs per mitotype per P:C diet. Vials were observed every 6 h to determine time to pupation. Numbers eclosing in 6 d was assayed following (dx.doi.org/10.17504/protocols.io.rqzd5x6), with the numbers of flies eclosing recorded daily for 6 d.

Female fecundity was assayed using flies that had been transferred from instant food to each experimental diet for one generation. Briefly, 47 female flies of each mitotype and diet, ranging from 3–5 d old were randomly transferred into separate 6 mL glass vials (Sigma-Aldrich) and allowed to oviposit for 24 h. The numbers of eggs in each vial were then counted under the microscope (dx.doi.org/10.17504/protocols.io.rq2d5ye).

Female fertility was assayed using flies of both the Alstonville and Dahomey mitotypes that had been raised on the 1:2 P:C and 1:16 P:C diets. Virgin females were collected and placed in vials containing the same diet in lots of two, establishing 10 vials/mitotype/diet. After 24 h one male with the Alstonville mitotype and one with the Dahomey mitotype were added to each vial. Flies were allowed to mate for 24 h, and then swapped to new vials twice, in 24 h intervals. All flies were then removed. Eclosing adults were counted, and data recorded as number of offspring per female (https://dx.doi.org/10.17504/protocols.io.r6yd9fw).

### Study 2: Population cages, reproducibility, and generalisability including two mitotypes and two diets

#### Population cages including two mitotypes and two diets

Studies followed those described above, but the diet was permuted over the 26 generations of the study. Generations 1–4 were fed 1:2 P:C laboratory diet, 5–20 the 1:16 P:C diet and 21–26 the 1:2 P:C diet. For these cage studies, the mtDNA frequency of larvae harbouring Dahomey or Alstonville mtDNA was determined by allele-specific PCR and independently corroborated by Sanger sequencing, as above using the ND4L forward 5’-TAAACAAACTAATCTAACTAATA-3’, reverse 3’-GGTTGTGATATATTATCTTATGG-5’ and reverse 3’-TATATTAATTGGTATTTTTTCTG-5’ primer (dx.doi.org/10.17504/protocols.io.rq2d5ye).

A second population cage study controlled for functionally significant accumulated nuclear mutations and tested the influence of natural diets. Flies harbouring the two mitotypes were maintained on instant *Drosophila* food for five generations and allowed to mate indiscriminately. Isofemale lines with a randomised nuclear genome seeded three cages with passionfruit (~1:2 P:C) and three with banana (~1:16 P:C) diets. The fruit diets were prepared with 150 g of passionfruit or banana in a standard base containing 1% agar, 0.1% nipagen, 0.1% propionic acid and 0.001% phosphoric acid. The final volume was set at 300ml (dx.doi.org/10.17504/protocols.io.sdfea3n). Frequencies of flies harbouring the distinct mitotypes was assayed as described above by allele-specific PCR.

In a third population cage study, the two white-eyed *D*. *melanogaster* mitotypes were independently competed against an inbred red-eyed strain of *D*. *simulans si*III collected by JWOB in east Africa. Frequencies of flies were determined by eye colour.

#### Reproducibility and generality of immature development

The next set of experiments tested whether differences in the data were robust in regards to the interactions between the mitochondrial and nuclear genomes, the diet, and the microbiome (dx.doi.org/10.17504/protocols.io.r6yd9fw). Four trials of development time were conducted by counting the number of adult female flies that eclosed over 3 d. First, we tested the control laboratory diets (1:2 and 1:16 P:C) at ~6 month intervals.

The second trial tested the influence of the nuclear genome. Here, each of the mtDNA genomes was introgressed into the Oregon R (OreR) and the Canton S backgrounds using balancer chromosomes followed by five generations of backcrossing [15].

The third trial tested whether laboratory diets biased the outcome. Here, we fed *Drosophila* food based on passionfruit and banana (~1:2 and ~1:16 P:C, respectively). Fruit diets were constructed as described above (dx.doi.org/10.17504/protocols.io.sdfea3n).

The fourth trial examined the influence of the microbiome. First, we aliquoted the homogenate of ground wild-caught flies into vials containing developing larvae. *Drosophila* adults were collected from an Orange Orchard near Wiesman’s Ferry, NSW and their microbiota used to seed a development time study. Flies were kept at 23° C and 50% humidity on a 12 h light/dark cycle. Second, we examined intragenerational microbiome changes. Guts of early third instar female larvae were removed. Genomic DNA was extracted from 6 sets of 4 individuals among the four treatment groups using the NucleoSpin Tissue XS DNA extraction kit (Machery-Nagel, Düren, Germany) following manufacturer’s instructions. The V4 region of the lrRNA gene was amplified by PCR, in duplicate for each sample, using the 515 forward 5’-GTGCCAGCMGCCGCGGTAA-3’ and 806 reverse 3’-GGACTACHVGGGTWTCTAAT-5’ primer. PCR products were pooled and sequenced on a MiSeq platform with 2 x 250 bp chemistry at the Ramaciotti Centre for Genomics (NSW, Australia). Paired-end sequences were merged into contigs, quality filtered and taxonomically classified using Mothur (http://www.mothur.org) and the associated MiSeq pipeline, but with minor changes (here, singleton contigs were removed after the pre-clustering step). The classification was achieved using the Ribosomal Database Project taxonomic outline (http://rdp.cme.msu.edu) with 60% confidence threshold, and a phylotype approach (genus level) used to compare communities. Each sample was rarefied to the same total number of sequences (n = 41859), to account for differential sequencing depth, and then converted to relative abundance for data analysis (dx.doi.org/10.17504/protocols.io.shjeb4n).

### Study 3: Testing the significance of candidate mutations

#### Quaternary and secondary structure modelling and assay for A+T rich repeat number variation

Preliminary analyses suggested the nonsynonymous mutation in complex I may be functional, but the changes in complex IV and V were not. Modelling was based on the high-resolution three-dimensional structure from *E*. *coli* [32], the reported vertebrate structure for complex I [33] and the supercomplex [34]. Complex I structural modelling included the high-resolution three-dimensional structure of *E*. *coli* [32]. We also modelled the recently reported vertebrate structure for complex I [33] and the supercomplex [34]. The sequences for 11 *Drosophila* lines, which were not part of this study, were included in determining if sites were fixed or segregating within *D*. *melanogaster*. The initial alignment was performed with CLC Bio Sequence Viewer 7 (https://www.qiagenbioinformatics.com/). Small manual adjustments were made to align highly conserved and homologues residues. Latter residues were identified on the 3-dimensional structure (PDB 3RKO) with software from Visual Molecular Dynamics (www.ks.uiuc.edu/vmd). The R-groups and their orientation were then considered in combination with the functional model that has been proposed for proton translocation.

To investigate the effect of the rRNA mutations on mitoribosomal function, we used the HIA method [35] with the following variations. First, the *D*. *melanogaster* mitochondrial sequence (GenBank: U37541.1) was aligned with the sequences of the Alstonville and Dahomey sequences (Accession numbers KP843842 and KP843845) using T-COFFEE [202]. The sites of mutation were placed on the three-dimensional structure of the human mitoribosome (PDB IDs: 3J9M and 3J9N) [85], due to its close evolutionary distance and its structural quality. To more closely inspect the impact of the lrRNA mutation on the function of the GTPase center, the structure of the ribosome-bound *E*. *coli* Elongation Factor Tu from *E*. *coli* (EF-Tu; PDB ID: 5AFI) was superimposed with the program UCSF Chimera [203] onto the human mitoribosomal structure.

We conducted continuous long-read Pacific BioSciences sequencing of the Alstonville and Dahomey mtDNA genomes because short-read sequencing is problematic. The sequence of the A + T rich region showed no differences from the published short-read sequences [84] and no differences in repeat number between mitotypes (NCBI Project accession #PRJNA397821). Mitochondria were extracted from adult flies [91]. The mtDNA was extracted using the DNeasy Blood and Tissue Kit (Qiagen 69582). Sequencing was performed using PacBio RSII Chemistry P6-C4, 10kb template preparation, and sequencing with 3.24 μg input DNA at the Ramaciotti Center. PacBio long-read sequencing of Alstonville and Dahomey mtDNA produced 882.56 MB of raw data. Read lengths averaged 5,573 bases with the longest read length of 24,822 bases and mean coverage of 40X for each mitotype (dx.doi.org/10.17504/protocols.io.r9xd97n).

#### Cellular assays

For in vitro assays intact mitochondria were isolated, and citrate synthase activity measured from female third instar wandering larvae [204]. Citrate synthase activity was measured using a SpectraMax Plus spectrophotometer at 23° C (Molecular Devices Corp., CA, USA) and expressed as μmol. min^−1^. mg^−1^of total protein. There were no differences in citrate synthase activity of each mitotype (t_10_ = 0.362, p = 0.725 and t_10_ = 0.479, p = 0.645 for the 1:2 and 1:16 P:C diets, respectively) (dx.doi.org/10.17504/protocols.io.rq4d5yw).

Enzymatic activity of electron transport system complexes I, IV and V were measured from female third instar wandering larvae in 96-well plate format at 23° C [204–206]. The specific activity of each complex was expressed as nmol per min per unit of citrate synthase activity (nmol. min^−1^. U CS^−1^) (dx.doi.org/10.17504/protocols.io.rq5d5y6 for complex I; dx.doi.org/10.17504/protocols.io.shieb4e for complex IV and dx.doi.org/10.17504/protocols.io.rq5d5y6 for complex V).

Mitochondrial oxygen consumption of mitochondria isolated from third instar wandering female larvae was assayed using the XF24 Extracellular Flux Analyser (Seahorse Bioscience, MA, USA) at 23° C using complex I and complex II substrates [91]. Respiration rates were expressed as oxygen consumption rate with the unit of pmol of oxygen consumption per min per unit of citrate synthase activity (pmol. min^−1^.U CS^−1^) (dx.doi.org/10.17504/protocols.io.rrud56w).

For the Western blot, groups of 10 female third instar wandering larvae were collected, frozen in liquid nitrogen and were homogenised in 2x lysis buffer (50 mM TRIS, pH 8.0, 300 mM NaCl, 2 mM EDTA, 1% SDS, 2% Triton X-100) with protease inhibitor cocktail (Roche 11873580001). Blots were labelled with monoclonal antibodies to the β-subunit of ATP synthase (Abcam AB14730) diluted 1/1500, actin (Abcam AB8224) diluted 1/25,000, and the NDUFS3 subunit of complex I (Abcam AB14711) diluted 1/800 [37] (dx.doi.org/10.17504/protocols.io.sd7ea9n).

For the Blue native polyacrylamide gel electrophoresis (BN-PAGE), mitochondria were extracted from female third instar wandering larvae. Mitochondria were solubilized with digitonin, and mitochondrial proteins separated by BN-PAGE [207] using the NativePAGE Novex Bis-Tris gel System (Invitrogen Life Technologies, MA, USA). Complex I activity was visualised by staining the gel in 2 mM Tris-HCl, pH 7.4, 0.1 mg/ml NADH and 2.5 mg/ml nitrotetrazolium Blue [207] (dx.doi.org/10.17504/protocols.io.r67d9hn).

### Study 4: An electron transport system complex I mutation in Dahomey drives the population cage results

#### Effect of dietary inhibitors on immature development

We treated larvae with the electron transport system inhibitor rotenone. Rotenone (Sigma R8875) was solubilised in DMSO to prepare a 5 mM stock. We first set up a dilution series of rotenone in the 1:2 P:C and 1:16 P:C diets to determine a rotenone concentration that did not impact survival. The dilution series contained 5 rotenone concentrations from 25 μM to 0.78 μM in steps that halved the concentration in each dilution. From the dilution series, we determined an assay concentration of 3.13 μM. These preliminary studies showed that DMSO slowed development of both mitotypes on both diets by ~3h, and increased mortality by ~15%. For 3 d development, we counted the numbers of flies eclosing in each diet using 5 replicates and then standardised them to controls (dx.doi.org/10.17504/protocols.io.rs8d6hw).

We also treated larvae with the ETS inhibitor paraquat. Paraquat was solubilised in water to a concentration of 5 mM and added to food to a final concentration of 3.13 μM. Mortality increased by ~40% compared to the controls. For 3 d development, we counted the numbers of flies eclosing in each diet using 5 replicates and then standardised them to controls (dx.doi.org/10.17504/protocols.io.rs8d6hw).

#### Effect of rotenone on complex I activity, SOD activity and larval weight

We conducted assays of complex I activity as described above (dx.doi.org/10.17504/protocols.io.rs8d6hw). SOD activity in third instar larvae was determined by a standard photometric assay [14]. The dry weight of third instar larvae was measured as previously described using a Sartorius Microbalance (AG Gottingen, Germany) [15].

#### Testing development times with a second pair of mitotypes

The number of Madang (Papua New Guinea) females with the ND4 mutation and Victoria Falls (Zimbabwe) females without the ND4 mutation was determined over a 3 d window as described above (dx.doi.org/10.17504/protocols.io.rqzd5x6).

### Study 5: Transcriptomics and metabolomics

Here, RNA and metabolites were extracted from female third instar wandering larvae sourced from the side of the bottle that had developed on 1:2 and 1:16 P:C diets [108, 208]. In uncrowded conditions, on a fixed light/dark regimen, larval wandering is highly synchronous and begins some 24 h before pupation (at 25° C) [108].

#### Transcriptomics

RNA was extracted from 4 replicates/mitotype/diet using a TruSeq kit (Illumina, CA, USA) with poly(A) pull-down. RNA quality was verified by Agilent Bioanalyser (Agilent Technologies, CA, USA) with all samples having an RNA Integrity Number greater than 8. Samples were sequenced on an Illumina Hiseq2000 sequencer at the Ramaciotti Center to produce 100 bp paired-end reads. At least 80 million read pairs were generated per sample. Reads were mapped to the NCBI *D*. *melanogaster* genome GCF_000001215.4_Release_6_plus_ISO1_MT using the Subread aligner [209] and assigned to genes using featureCounts [210]. Genes were filtered as not expressed if they failed to achieve at least 0.25 counts per million in at least 4 samples. Trimmed means of M-values (TMM) scale normalisation was applied [211] and read counts were transformed to log2-counts-per-million using the edgeR package [211]. Statistical bioinformatics analysis used the limma package [212]. To adjust for unwanted nuisance technical effects, extra surrogate variables were estimated by performing a singular value decomposition of the residuals, with emphasis on highly variable genes. Differential expression between groups was assessed using empirical Bayes t-statistics allowing for an abundance trend in the standard errors and robust estimation of the Bayesian hyperparameters [213]. Benjamini-Hochberg’s method was used to control the false discovery rate (FDR). KEGG pathway analyses were conducted using the kegga function in limma. To produce heatmaps, log-count-per-million values were first batch corrected using the removeBatchEffect function of the limma package [212] (dx.doi.org/10.17504/protocols.io.rs9d6h6).

We employed RT-qPCR to validate the RNA seq data and quantify select genes for pathway analyses. For RNA extraction, 6 female third instar wandering larvae were homogenised in TRI reagent (Sigma) in a Precellys 24 homogeniser (Bertin Technologies, île-de-France, France). RNA was extracted with the standard TRI reagent protocol. 1.5 μg of total RNA was treated with DNase I Amplification Grade (Sigma). cDNA was prepared from 1.5 μg RNA template in 20 μl reaction mixture using a ProtoScript cDNA synthesis kit (New England Biolabs, MA, USA). The comparative cycle threshold (Ct) method was used to analyse the RT-qPCR studies.

The expression of mtDNA genes was quantified by the following primers: srRNA forward 5’-TGGCGGTATTTTAGTCTATCT-3’, reverse 3’-AAGCTACACCTTGATCTGATA-5’; lrRNA forward 5’-AGTCTAACCTGCCCACTGAAA-3’, reverse 3’-AGGGTCTTCTCGTCTTTTAAA-5’. ATP6 forward 5’-AGAATAGCGGGTGTTCCTTGA-3’, reverse 3’-CCATCAGGTCATAATGGATCT-5’; ND4 forward 5’-AACTGGAGCTTCAACATGAGC-3’, reverse 3’-AGCCAGAACGTTTACAAGCTG-5’; and ND6 forward 5’-AATTCATCCATTAGCTTTAGG-3’, reverse 3’-AGAGGCTAAAGATGTTACGTA-5’.

The expression of nuclear genes was quantified by the following primers:

*bmm* forward 5’-AAGTATGCACCGCATCTGTTG-3’, reverse 3’-CAAATCGCAGAGGAGACAGC-5’; *CrebB* forward 5’-ATGGACAACAGCATCGTCGA-3’, reverse 3’-ACGACATCGACCACGTCATT-5’; *eloF* forward 5’-GCACATTGATTGGCTATCTGCT-3’, reverse 3’- GATTTGGTAGGCTTTCAGGACA-5’; *ERR* forward 5’-GACCTCTCTATCCTGCGATTTG-3’, reverse 3’-CCACTTGTACCACTTCCTTTCAG-5’; *GlyP* forward 5’-TCCACCCTGAGGGACTACTAC-3’, reverse 3’-GGTGTTGGTCAGTGAGCGAC-5’; *GstE1* forward 5’- TCTTCTTCGATGCCAGTGTAATC-3’, reverse 3’-CACTGGCATCGAAGAAGAGAC-5’ *GstE5* forward 5’- GGTAACTACATTTGGGACTCGC-3’, reverse 3’- ATCTCTGGGATACAGGGCATC-5’; *Ilp2* forward 5’-ATGAGCAAGCCTTTGTCCTTC-3’, reverse 3’-ACCTCGTTGAGCTTTTCACTG-5’; *N* forward 5’-GTCGGCGACTACTGTGAACAC-3’, reverse 3’-GTTGCGAAAGGTCACCTGACA-5’; *TFAM* 5’-AACCGCTGACTCCCTACTTTC-3’, reverse 3’- CGACGGTGGTAATCTGGGG-5’; and *Zw* forward 5’- TTTGACGGCAAGATTCCGCA-3’, reverse 3’- CACCAGAGCGTGGGGTAGA-5’.

The RT-qPCR program included an initial step of 95° C, followed by 40 cycles of 95° C for 10 s and 60° C for 30 s. To verify that a single product was amplified amplification was followed by a melting curve from 72° C to 95° C, rising by steps of 0.5° C [14]. The mRNA levels were expressed as the relative fold change against the normalised *rp49* (*rp49* forward 5’-AGCATACAGGCCCAAGATCG-3’, reverse 3’-TGTTGTCGATACCCTTGGGC-5.) and *Actin88* (*Actin88* forward 5’-TCGATCATGAAGTGCGACGT-3’, reverse 3’-ACCGATCCAGACGGAGTACT-5’) mRNA. RNA seq studies showed that the expression of *Rp49* and *Actin88* mRNA were not influenced by the range of diets included in this study. t-tests were used to determine significance. Benjamini-Hochberg’s correction was used to control the FDR (dx.doi.org/10.17504/protocols.io.rtcd6iw).

#### Metabolomics

Female third instar wandering larvae were weighed, and methanol was added to make up each sample to 20 mg/ml. Metabolites were extracted using an ultrasonic probe (30 s), 1 h incubation at 4° C and then centrifugation to remove particulates. 100 μl aliquots of the supernatant were derivatised [214] before mass spectrometric interrogation with an Agilent GC/MSD system (Agilent Technologies, CA, USA) controlled by Chemstation software. The GC inlet temperature was set to 230° C. 1 μl of derivatised sample was injected in splitless mode, using helium as a carrier gas at constant-flow of 1 ml/min. Chromatographic separation was performed on a 30 m SH-RXi-5Sil MS column (Shimadzu, NSW, Australia) with 0.25 mm internal diameter and 0.25 μm film thickness. The oven temperature was programmed at 70° C for 2 min, then ramped at 15° C/min to 320° C, and held 8 min. Electron ionisation mass spectra were recorded at 1.4 scans/s over the range *m/z* 50–700. The MSD auxiliary temperature, source temperature, and quadrupole temperature were set to 280° C, 230° C, and 150° C, respectively. Analytes were identified using the NIST 2011-Wiley Mass Spectra Library. GC peaks containing mass spectra with a match quality (spectral purity) of more than 70% were considered to be tentatively identified. No internal standard was employed. Normalisation was performed by maintaining a constant sample mass per volume. Peak areas were log-transformed and statistical analysis used the limma package [212]. To determine the isomers of sugars we employed standards for 2-deoxy-D-glucose (Sigma D8375), fructose (Sigma F0127), galactose (Sigma PHR1206), glucose-6-phosphate (Sigma G7879), maltose (Sigma M5895) and mannose (Sigma M6020). Identified sugars with multiple peaks were then compared to the retention time of the standards to identify the most likely isomer. Metabolites not hypothesised to be from *Drosophila* were excluded from subsequent analyses. One of these was Longicamphor that we hypothesised was derived from the burning of *Camphor laurel* woodchips in making the treacle used in the diets. Benjamini-Hochberg’s correction was used to control the FDR (dx.doi.org/10.17504/protocols.io.rtad6ie).

#### Inhibitor assay

For the glycolysis inhibitor assay 2-Deoxy-D-Glucose (Sigma D8375) was solubilised in water to prepare a 500 mM stock. The stock solutions were added to the 1:2 and 1:16 P:C diets to a final concentration of 5 mM 2-Deoxy-D-Glucose. Females that eclosed in a 3 d window were collected and counted, and eclosion percentage was calculated.

### Study 6: Response of *Drosophila* larvae to the 1:2 P:C and 1:16 P:C diets

#### Basal ROS levels and oxidative stress

Female, late wandering, third-instar larvae were collected, washed in 1x phosphate buffered saline and added to 1.5 ml Eppendorf tubes containing 100 μl of mitochondrial isolation buffer (154 mM KCl, 1 mM EDTA, ph 7.4), mitochondria were isolated, and Bradford assay was conducted to measure protein. Basal ROS production was measured by Amplex Red assay [204] (dx.doi.org/10.17504/protocols.io.r69d9h6).

#### Superoxide levels

Larvae were dissected in PBS to produce muscle fillets. Fillets were incubated in 20 mM 2',7'-dichlorodihydrofluorescein diacetate (H_2_DCFDA) for 30 min at 23° C. Fillets were washed twice in PBS, fixed in 2% paraformaldehyde for 10 min at 23° C, mounted on glass slides and imaged by confocal laser scanning microscope at excitation/emission 495/520 nm. Pixel intensity was measured by ImageJ (https://imagej.nih.gov/ij/) corrected to the background measurements (dx.doi.org/10.17504/protocols.io.ufuetnw).

#### MtDNA copy number and steady-state ATP levels

To determine mtDNA copy number, total DNA was extracted using DNeasy Blood and Tissue Kit (Qiagen 69582). The relative fold change of mtDNA copy number was determined by quantitative PCR (qPCR) using a 72 well Rotorgene-3000 (Corbett Research, Cambridge, UK) with SYBR Green (Bio-Rad, CA, USA). The mtDNA copy number was quantified by amplifying ND4 and the nuclear gene *Act88* and corroborated independently by amplifying lrRNA and the nuclear gene *rp49* as previously described. The mtDNA copy number was expressed as fold change of mean mtDNA copy number relative to Alstonville (dx.doi.org/10.17504/protocols.io.rtdd6i6).

ATP was extracted from female third instar wandering larvae harbouring Alstonville and Dahomey mtDNA raised on 1:2 P:C and 1:16 P:C diets (7 replicates/mitotype/diet) [215]. The extracted metabolites were analysed using liquid chromatography (LC) electrospray ionisation tandem mass spectrometry (ESI-MS/MS) [216]. ATP levels were determined by quantifying the area under the curve (dx.doi.org/10.17504/protocols.io.rted6je).

### Study 7: Mitohormetic responses in Dahomey larvae fed 1:16 P:C food

To test specific hypotheses, we replaced sucrose as the dietary sugar. The 1:16 P:C diet was prepared without the addition of sucrose. Then, 200 ml of food was combined with 1.87 g of either sucrose (Sigma S0389) as the control, sorbitol (Sigma S1876), fructose (Sigma F0127), mannose (Sigma M6020), fucose (Sigma F2252) or xylose (Sigma X3877). Each new diet was poured into 8 bottles. Equal amounts of eggs harbouring Alstonville or Dahomey mtDNA were added to each food and microbiome was added after 2 d. Flies were kept at 23° C, 50% humidity on a 12 h light/dark cycle. Emerging adult female flies were counted over 3 d, and percentage eclosion of each mitotype was calculated (dx.doi.org/10.17504/protocols.io.rtfd6jn).

For inhibitors, freshly prepared aldose reductase (polyol pathway) inhibitor (Epalrestat, Sigma SML0527) and carnitine palmitoyltransferase-1 inhibitor (Etomoxir, Sigma E1905) were solubilised in water to make a 5 mM stock. The stock solutions were added to the 1:16 P:C diet to final concentrations of 25 μM Epalrestat, 12.5 μM Etomoxir. Methodology followed that described above for 2-Deoxy-D-Glucose.

#### Upregulation of the polyol pathway in Dahomey

Fructose levels were quantified using a photometric kit following the manufacturer’s instructions (Abacm AB83380) (dx.doi.org/10.17504/protocols.io.rv8d69w).

To quantify larval feeding, 50 second instar female larvae from each mitotype were transferred to a petri dish containing the corresponding dye-labelled food. Dye-labelled foods were produced by combining 72 ml of the food while at 60° C, with 8 ml FD&C blue 1 dye (0.5% w/v). Larvae were allowed to feed for 60 min, and 48 larvae with dye visible in their guts were collected, homogenized and absorbance measured (dx.doi.org/10.17504/protocols.io.rw7d7hn).

#### Increased β-oxidation of fatty acids in Dahomey

Triglyceride levels were quantified using a photometric kit following the manufacturer’s instructions (Abcam AB65336) (dx.doi.org/10.17504/protocols.io.rv9d696).

The ß**-**oxidation assay was modified from adults for larvae [217]. Briefly, larvae were collected 36 h after egg collection and fed with 1 μCi of ^14^C-labelled palmitic acid (prepared in 1:16 P:C diet) for 9 d. Groups of 10 were transferred to glass vials (20 ml), and KOH-saturated (100 μl of 5% KOH) filter paper (2.1 cm diameter circle, 1 μm pore) suspended above the larvae. The radiolabelled CO_2_ from larval respiration was trapped as potassium bicarbonate with KOH-saturated filter paper. After 5 h, this KOH-saturated filter paper was transferred to a 6 ml plastic scintillation vial containing 4 ml of scintillation cocktail (Ecoscint A) and radioactivity was measured using a scintillation counter. The amount of CO_2_ generated from ß-oxidation was expressed as DPM. larva^-1^. h^-1^ (dx.doi.org/10.17504/protocols.io.rq3d5yn).

Acetyl-CoA from both mitochondrial and cytosolic extractions were quantified using a photometric kit (Abcam AB87546) following the manufacturer’s instructions (dx.doi.org/10.17504/protocols.io.rv5d686).

NAD^+^ and NADH metabolites were extracted from female third instar wandering larvae harbouring Alstonville and Dahomey mtDNA raised on 1:2 P:C and 1:16 P:C diets (7 replicates/mitotype/diet) [215]. The extracted metabolites were analysed using liquid chromatography (LC) electrospray ionisation tandem mass spectrometry (ESI-MS/MS) [216]. The NAD^+^/NADH ratio was calculated as relative differences in peak areas between NAD^+^ and NADH metabolites (dx.doi.org/10.17504/protocols.io.rted6je).

To quantify larvae starvation survival, 12 early third instar female larvae from each mitotype were put into 6 vials. Each vial contained a 6 x 3 cm piece of filter paper, wetted with ddH_2_O twice daily. Larvae were observed 4 times daily by gentle prodding to determine if they were alive. Larvae that died within the first 24 h were excluded as death was due to handling. Mean time to death was recorded (dx.doi.org/10.17504/protocols.io.rw9d7h6).

### Study 8: Mitohormetic responses in Alstonville larvae fed 1:16 P:C food

#### Increased glycogen metabolism

Glycogen levels were quantified using a photometric kit (AB169558) following the manufacturer’s instructions (dx.doi.org/10.17504/protocols.io.rv7d69n)

To quantify larval movement, 48 third instar female larvae were placed in 0.5% agar in Petri dishes. Individual larvae placed in quadrants of the petri dish were observed for 60 s, and larvae trails were immediately traced onto the lid. The distance moved by each larva was measured independently by two investigators and the mean movement/larvae determined. The intraclass correlation between investigators was 0.96 showing high repeatability of the measurement (dx.doi.org/10.17504/protocols.io.rxad7ie).

To test specific hypotheses, we replaced sucrose as the dietary sugar as described above with 1.868 g of gluconate (Sigma G1951) in 200 ml of food (dx.doi.org/10.17504/protocols.io.rtfd6jn).

#### Upregulation of the pentose phosphate pathway in Alstonville

Glucose 6-phosphate-dehydrogenase (G6PD) activity was quantified in 96-well microplates at 23° C [218]. Enzyme activity was expressed as nmol. min^−1^. mg^−1^of protein (dx.doi.org/10.17504/protocols.io.rwbd7an).

### Statistical analyses

Unless otherwise stated, all data are biological replicates and statistically analysed by ANOVA followed by Student’s t-tests to determine difference (JMP software 12, SAS Institute, NC, USA). Biological replicates are parallel measurements of biologically distinct samples. Where the numbers of Dahomey larvae eclosing in 3 d was compared between dietary sugars (sorbitol, fructose, mannose, fucose, xylose and gluconate) and the control diet with the diets supplemented with an inhibitor (Epalrestat, and Etomoxir)we conducted Dunnett’s tests. Data were checked for normality using a Shapiro-Wilks W test and outliers removed before statistical analyses using box plots. Values that were greater than ± 1.5 interquartile range were categorised as an outlier and excluded from the data set. No statistical methods were used to predetermine sample size.

## Supporting information

S1 Fig Population cage studies. **(A**) Illustration of the steps for all population cage studies (~850 flies/cage). Eggs and then squashed flies were introduced into bottles. The squashed flies contained gut microbes and standardised the microbiome each generation. Flies developed in the bottles and the stopper was removed so flies could randomly mate in the population cages for 3 d. Eggs were collected on oviposition resources in population cages and then washed 3x in 0.12% bleach to surface sterilise them. **(B)** Illustration of the initial population cage study. The four mitotypes that competed against each other had a constant *w*^1118^ nuclear genetic background with unique mtDNA types (Alst, Alstonville; Dah, Dahomey; Jap, Japan; *w*^1118^). The four diets had 1:2, 1:4, 1:8 and 1:16 Protein: Carbohydrate (P:C) ratios (n = 3 cages for each diet). (**C**) Time to pupation (n = 100 larvae/mitotype/diet with 32 Alstonville larvae and 8 Dahomey larvae not reaching pupation on the 1:2 P:C diet, and with 25 Alstonville larvae and 5 Dahomey larvae not reaching pupation). (**D**) Percentage of flies eclosing in a 6 d window (n = 4 bottles/mitotype/diet). (**E**) Fertility (n = 10 vials/mitotype/diet). Plotted data were mean± s.e.m. * p< 0.05 as calculated by t-tests (see text).
(TIF)

S2 Fig Microbiome in larval guts. The proportion of bacteria was influenced by mitotype when larvae were fed the 1:16 P:C food but not the 1:2 P:C diet (n = 6 biological rep/mitotype/diet for the 1:2 P:C diet and n = 9 biological rep/mitotype/diet for the 1:16 P:C diet, see text).
(TIF)

S3 Fig Quaternary structural modelling. (**A**) The *Drosophila* supercomplex showing the predicted movement of protons in yellow. The V161L ND4 mutation is circled in yellow and the D40N COIII mutation in orange. (**B**) COIII mutation showing the site of mutation in orange circle and structurally related residues. The mutation site does not appear to interact with any other residues.
(TIF)

S4 Fig Oxygen consumption rate. **(A)** Activity of complex IV (n = 5 rep/mitotype/diet). **(B)** Activity of complex V (n = 6 rep/mitotype on the 1:2 P:C diet, and n = 7 rep/mitotype on the 1:16 P:C diet). **(C)** Oxygen consumption rate of extracted mitochondria with succinate as the substrate (n = 6 biological rep/mitotype/diet) did not differ significantly between mitotypes on either diet (see text). **(D)** Superoxide of muscle tissue stained with H2DCFDA (left panel) and their quantified pixel intensity (right panel, n = 6 rep/mitotype/diet). ANOVA showed significant main effects of mitotype, diet and their interaction (F_1,20_ = 7.19, p = 0.01, F_1,20_ = 40.76, p< 0.0001. F_1,20_ = 8.23, p = 0.01). t-test showed a significant difference in superoxide on the 1:2 P:C diet (t_10_ = 5.165, p = 0.0004), but no difference on the 1:16 P:C diet (t_10_ = 0.11, p = 0.91). Bars show mean± s.e.m.
(TIF)

S5 Fig Rotenone treatment assays showing grouping. (**A**) Adding rotenone to the Alstonville diet created a Dahomey phenocopy. This phenocopy developed more quickly than Alstonville controls when fed the 1:16 P:C food showing that partial inhibition of complex I was beneficial. Adding rotenone to the Dahomey fly food created a disease model and these larvae developed more slowly on both diets (n = 5 biological rep/mitotype/diet with and without rotenone treatment). (**B**) Complex I activity was decreased in the phenocopy, mimicking the Dahomey mitotype (n = 5 biological rep/mitotype/diet with and without rotenone treatment). (**C**) SOD activity increased in the rotenone treatment on the 1:2 P:C diet. On both diets SOD activity in the phenocopy was not different from the Dahomey mitotype (n = 5 biological rep/mitotype/diet with and without rotenone treatment). (**D**) Weight of the phenocopy was significantly different from the Dahomey mitotype on both diets (n = 5 biological rep/mitotype/diet with and without rotenone treatment). Bars (mean ± s.e.m. Groups not connected by the same letter differ significantly, according to LSMeans differences t test.
(TIF)

S6 Fig Paraquat treatment assay. Alstonville larvae treated with paraquat produced a phenocopy of the Dahomey control. ANOVA of the effects on development showed a significant effect of paraquat treatment (F_1, 32_ = 22.97, p< 0.0001) but no significant effect of mitotype or diet (F_1, 32_ = 2.67, p = 0.11, F_1, 32_ = 0, p = 1, respectively). In regards to the two-way interactions, mitotype-by-diet, diet-by-paraquat were significant and mitotype-by-paraquat were significant (F_1, 32_ = 29.09, p< 0.0001, F_1, 32_ = 16.83, p = 0.0003, F_1, 32_ = 10.52, p = 0.003, respectively). The three-way interaction was significant (F_1, 32_ = 26.56, p< 0.0001). Conducting a t-test on the Dahomey control and Alstonville paraquat treatment (phenocopy) showed no difference on the 1:2 (t_8_ = 1.68, p = 0.13) or 1:16 (t_8_ = 2.17, p = 0.06) P:C diets (n = 5 biological rep/mitotype/diet with and without paraquat treatment). Bars (mean± s.e.m). Groups not connected by the same letter differ significantly, according to LSMeans differences t test. N.S. denotes not significant (p> 0.05) by t-test.
(TIF)

S7 Fig Glucose-6-phosphate dehydrogenase (G6PD) activity. Activity was determined spectrophotometrically from the rate of reduction of NADP (n = 8 biological rep/mitotype). Bars (mean ± s.e.m). * p< 0.05, as calculated by t-tests (see text).
(TIF)

S1 Table Differences between the mitochondrial genomes of the Dahomey, Madang, Alstonville and Victoria Falls fly mitotypes. Position is taken from the alignment of GenBank No’s KP843845, KP843849, KP843842 and KP843854, respectively accessed on 12 April 2018. Syn is synonymous, Nonsyn is nonsynonymous, ItSpace is intervening spacer region and Con is consensus. ^1^ Complex V (M185I). ^2^ Complex IV (D40N), ^3^ Complex I (V161L), ^4^ The G499A (complementary strand).
(DOCX)

S2 Table RNA-seq results for Dahomey vs Alstonville with FDR< 0.05 (**A**) 1:2 P:C diet (**B**) 1:16 P:C diet. Positive fold change indicates up-regulated in Dahomey, while negative fold change indicates up-regulated in Alstonville.
(XLSX)

S3 Table KEGG pathways differentially expressed between mitotypes according to RNA-seq profiling (P< 0.01). **(A)** Alstonville up-regulated/Dahomey down-regulated when larvae are fed the 1:2 P:C food. **(B)** Alstonville down-regulated/Dahomey up-regulated when larvae are fed the 1:2 P:C food. **(C)** Dahomey up-regulated/Alstonville down-regulated when larvae are fed the 1:16 P:C food. **(D)** Dahomey down-regulated/Alstonville up-regulated when larvae are fed the 1:16 P:C food. Columns show the number of genes in the pathway (N), the number that are significantly up (Up) and down (Down) regulated and the P-values corresponding to the up and down counts.
(DOCX)

S4 Table Top 5 Gene Ontology terms comparing Dahomey with Alstonville on the 1:2 P:C and 1:16 P:C diets. **(A)** Alstonville up-regulated/Dahomey down-regulated when larvae are fed the 1:2 P:C food. **(B)** Alstonville down-regulated/Dahomey up-regulated when larvae are fed the 1:2 P:C food. **(C)** Dahomey up-regulated/Alstonville down-regulated when larvae are fed the 1:16 P:C food **(D)** Dahomey down-regulated/Alstonville up-regulated when larvae are fed the 1:16 P:C food. Columns show the number of genes in the pathway (N), the number that are significantly up (Up) and down (Down) regulated and the P-values corresponding to the up and down counts.
(DOCX)

S5 Table Differentially expressed metabolites from whole third instar female wandering larvae as assessed by GC/MS for the **(A)** 1:2 P:C diet and **(B)** 1:16 P:C diet. Values are false discovery rates (FDR). (+) Indicates up-regulated in Dahomey while (-) indicates up-regulated in Alstonville. Peak area is relative to Alstonville larvae. Peak area value is mean± s.e.m.
(DOCX)

S6 Table RNA-seq and RT-qPCR results for Dahomey vs Alstonville. (**A**) 1:2 P:C diet (**B**) The 1:16 P:C diet. Values are false discovery rates (FDR) as determined by moderated t-tests for RNA-seq or t-test for RT-qPCR. (+) Indicates up-regulated in Dahomey while (-) indicates up-regulated in Alstonville. MtDNA genes were: rRNA is *ribosomal ribonucleic acid*, ATP6 is *ATPase subunit 6*, ND is *NADH-ubiquinone oxidoreductase*. Nuclear genes were: *Bmm* is *brummer*, *CrebB* is *Cyclic-AMP response element binding protein B*, *eloF is elongase F*, *ERR* is *estrogen-related receptor*, *GlyP* is *Glycogen phosphorylase*, *GstE1* is *Glutathione S transferase E1*, *GstE5* is *Glutathione S transferase E5*, *Ilp2* is *Insulin-like peptide 2*, *N* is *Notch*, *TFAM* is *mitochondrial transcription factor A*, *Zw* is *Zwischenferment*.
(DOCX)

S7 Table Differentially expressed mitochondrial ribosomal proteins in larvae fed the 1:16 P:C diet. Value are false discovery rates (FDR) as determined by moderated t-tests for RNA-seq. (+) Indicates up-regulated in Dahomey while (-) indicates up-regulated in Alstonville.
(DOCX)

S1 Data Additional data used in the generation of figures in the manuscript and supporting information. (XLSX)
