# Supplementary figures and images for: Genotype to phenotype: Diet-by-mitochondrial DNA haplotype interactions drive metabolic flexibility and organismal fitness
Source: PLoS Genet. 2018 Nov 6;14(11):e1007735. doi: 10.1371/journal.pgen.1007735 (PMC6219761; doi:10.1371/journal.pgen.1007735)

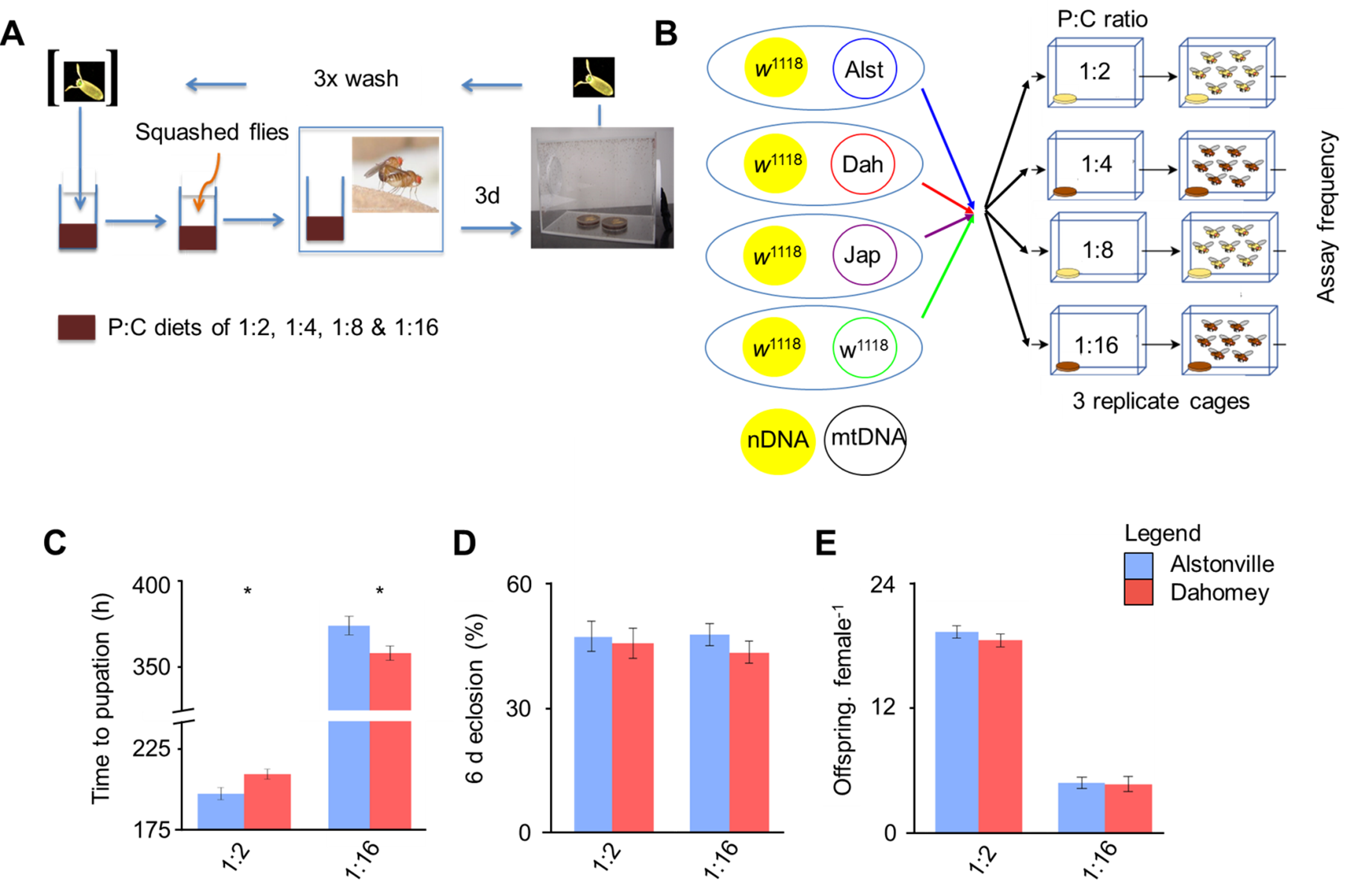

Supplement: S1 Fig — (A) Illustration of the steps for all population cage studies (~850 flies/cage). Eggs and then squashed flies were introduced into bottles. The squashed flies contained gut microbes and standardised the microbiome each generation. Flies developed in the bottles and the stopper was removed so flies could randomly mate in the population cages for 3 d. Eggs were collected on oviposition resources in population cages and then washed 3x in 0.12% bleach to surface sterilise them. (B) Illustration of the initial population cage study. The four mitotypes that competed against each other had a constant w1118 nuclear genetic background with unique mtDNA types (Alst, Alstonville; Dah, Dahomey; Jap, Japan; w1118). The four diets had 1:2, 1:4, 1:8 and 1:16 Protein: Carbohydrate (P:C) ratios (n = 3 cages for each diet). (C) Time to pupation (n = 100 larvae/mitotype/diet with 32 Alstonville larvae and 8 Dahomey larvae not reaching pupation on the 1:2 P:C diet, and with 25 Alstonville larvae and 5 Dahomey larvae not reaching pupation). (D) Percentage of flies eclosing in a 6 d window (n = 4 bottles/mitotype/diet). (E) Fertility (n = 10 vials/mitotype/diet). Plotted data were mean± s.e.m. * p< 0.05 as calculated by t-tests (see text). (TIF) [file pgen.1007735.s001.TIF]

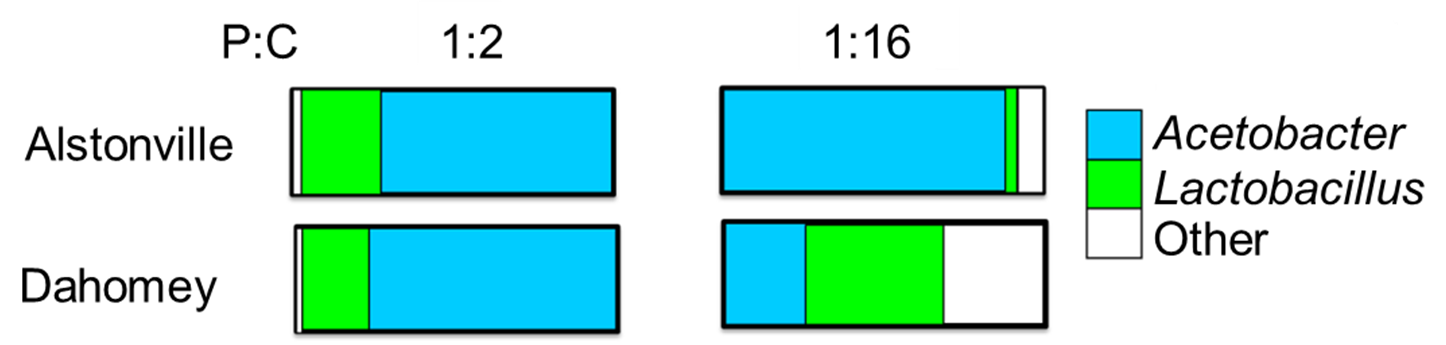

Supplement: S2 Fig — The proportion of bacteria was influenced by mitotype when larvae were fed the 1:16 P:C food but not the 1:2 P:C diet (n = 6 biological rep/mitotype/diet for the 1:2 P:C diet and n = 9 biological rep/mitotype/diet for the 1:16 P:C diet, see text). (TIF) [file pgen.1007735.s002.TIF]

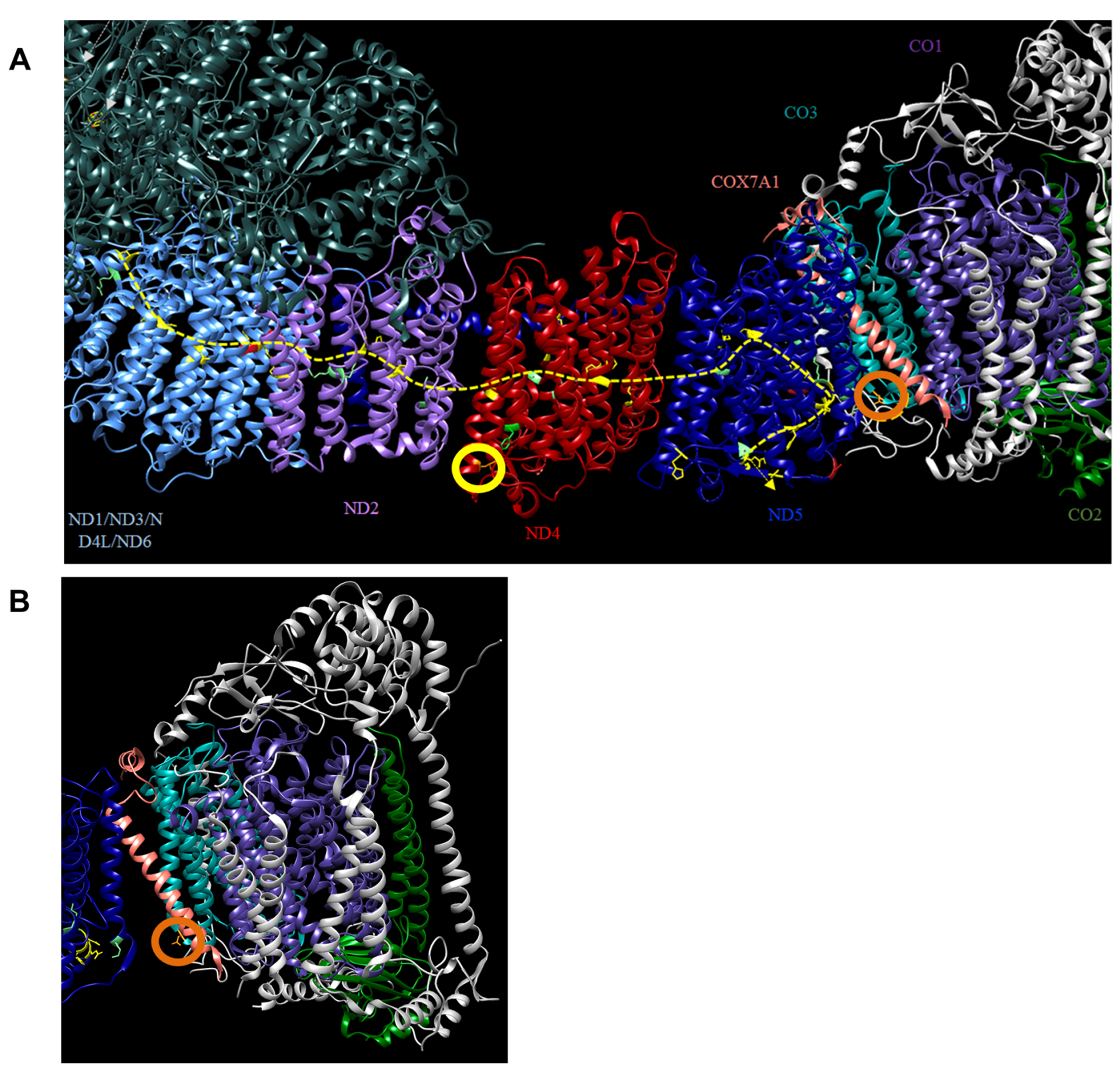

Supplement: S3 Fig — (A) The Drosophila supercomplex showing the predicted movement of protons in yellow. The V161L ND4 mutation is circled in yellow and the D40N COIII mutation in orange. (B) COIII mutation showing the site of mutation in orange circle and structurally related residues. The mutation site does not appear to interact with any other residues. (TIF) [file pgen.1007735.s003.TIF]

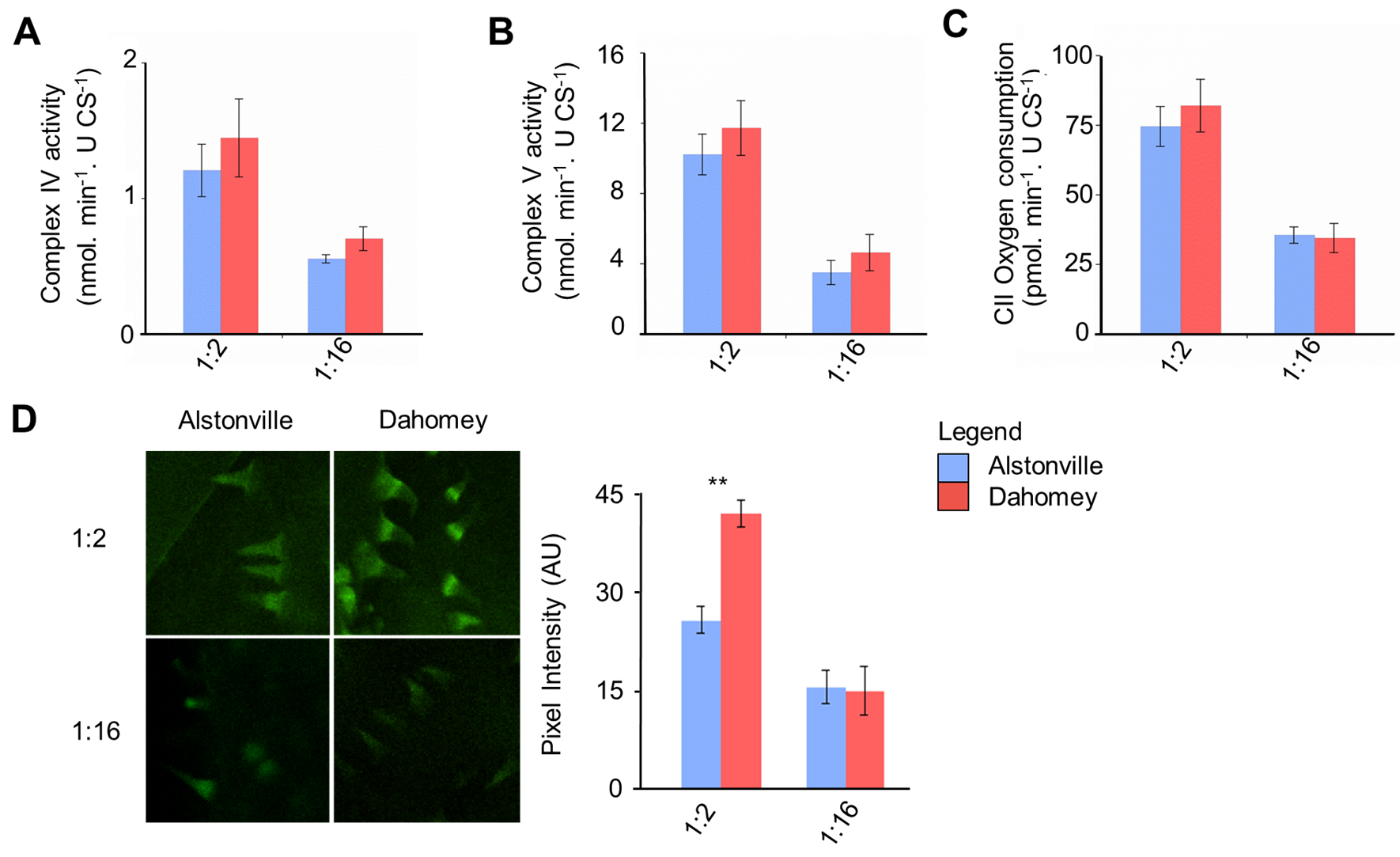

Supplement: S4 Fig — (A) Activity of complex IV (n = 5 rep/mitotype/diet). (B) Activity of complex V (n = 6 rep/mitotype on the 1:2 P:C diet, and n = 7 rep/mitotype on the 1:16 P:C diet). (C) Oxygen consumption rate of extracted mitochondria with succinate as the substrate (n = 6 biological rep/mitotype/diet) did not differ significantly between mitotypes on either diet (see text). (D) Superoxide of muscle tissue stained with H2DCFDA (left panel) and their quantified pixel intensity (right panel, n = 6 rep/mitotype/diet). ANOVA showed significant main effects of mitotype, diet and their interaction (F1,20 = 7.19, p = 0.01, F1,20 = 40.76, p< 0.0001. F1,20 = 8.23, p = 0.01). t-test showed a significant difference in superoxide on the 1:2 P:C diet (t10 = 5.165, p = 0.0004), but no difference on the 1:16 P:C diet (t10 = 0.11, p = 0.91). Bars show mean± s.e.m. (TIF) [file pgen.1007735.s004.tif]

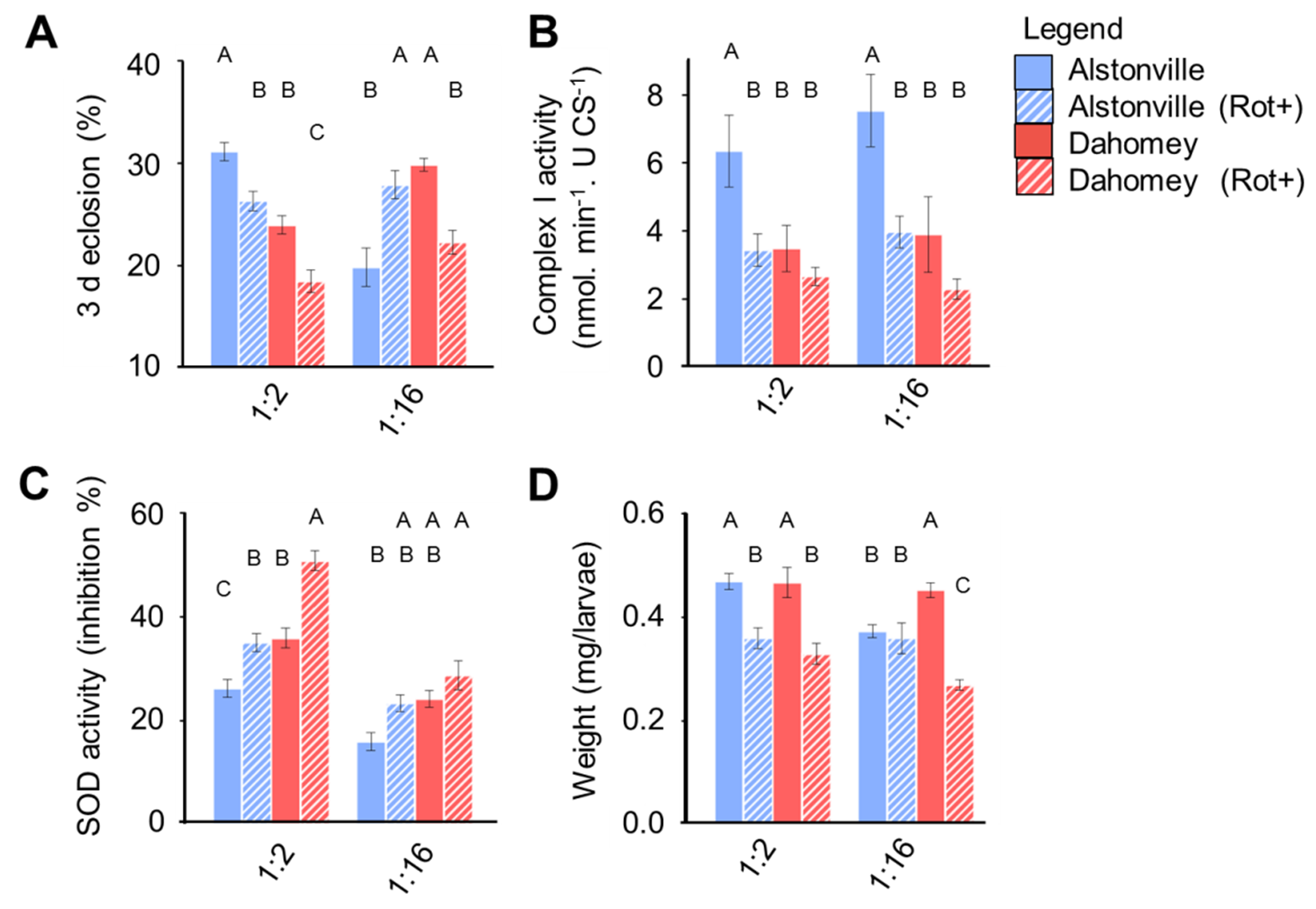

Supplement: S5 Fig — (A) Adding rotenone to the Alstonville diet created a Dahomey phenocopy. This phenocopy developed more quickly than Alstonville controls when fed the 1:16 P:C food showing that partial inhibition of complex I was beneficial. Adding rotenone to the Dahomey fly food created a disease model and these larvae developed more slowly on both diets (n = 5 biological rep/mitotype/diet with and without rotenone treatment). (B) Complex I activity was decreased in the phenocopy, mimicking the Dahomey mitotype (n = 5 biological rep/mitotype/diet with and without rotenone treatment). (C) SOD activity increased in the rotenone treatment on the 1:2 P:C diet. On both diets SOD activity in the phenocopy was not different from the Dahomey mitotype (n = 5 biological rep/mitotype/diet with and without rotenone treatment). (D) Weight of the phenocopy was significantly different from the Dahomey mitotype on both diets (n = 5 biological rep/mitotype/diet with and without rotenone treatment). Bars (mean ± s.e.m. Groups not connected by the same letter differ significantly, according to LSMeans differences t test. (TIF) [file pgen.1007735.s005.TIF]

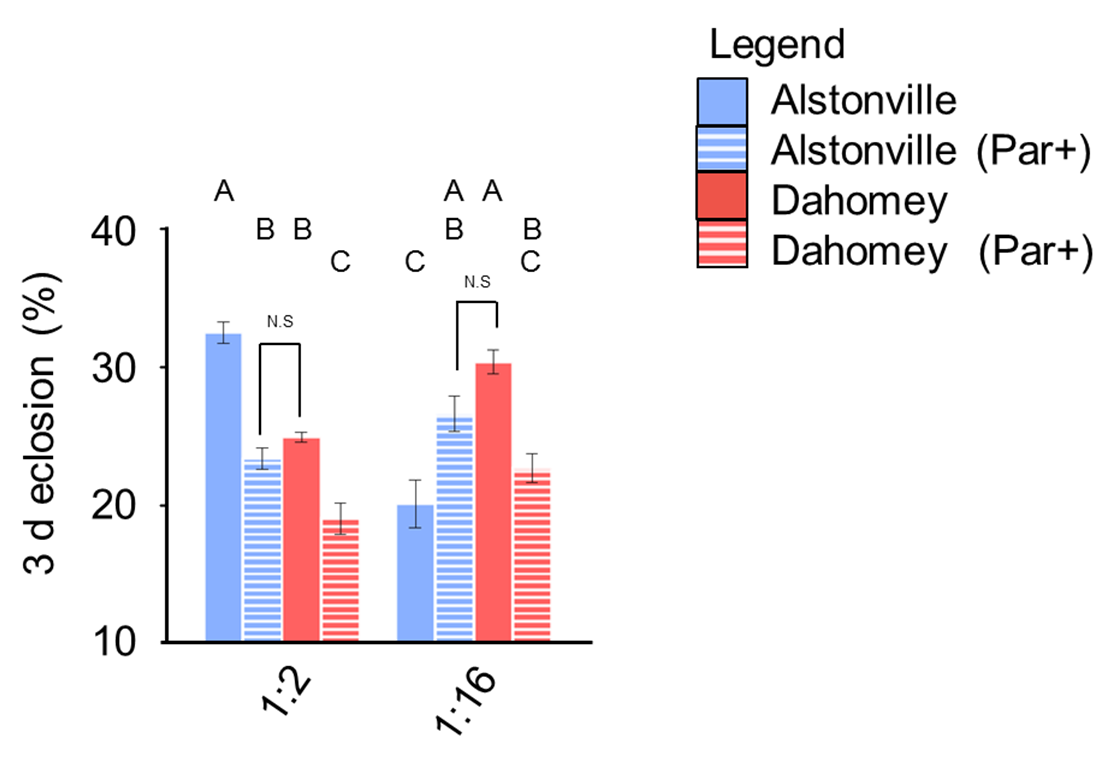

Supplement: S6 Fig — Alstonville larvae treated with paraquat produced a phenocopy of the Dahomey control. ANOVA of the effects on development showed a significant effect of paraquat treatment (F1, 32 = 22.97, p< 0.0001) but no significant effect of mitotype or diet (F1, 32 = 2.67, p = 0.11, F1, 32 = 0, p = 1, respectively). In regards to the two-way interactions, mitotype-by-diet, diet-by-paraquat were significant and mitotype-by-paraquat were significant (F1, 32 = 29.09, p< 0.0001, F1, 32 = 16.83, p = 0.0003, F1, 32 = 10.52, p = 0.003, respectively). The three-way interaction was significant (F1, 32 = 26.56, p< 0.0001). Conducting a t-test on the Dahomey control and Alstonville paraquat treatment (phenocopy) showed no difference on the 1:2 (t8 = 1.68, p = 0.13) or 1:16 (t8 = 2.17, p = 0.06) P:C diets (n = 5 biological rep/mitotype/diet with and without paraquat treatment). Bars (mean± s.e.m). Groups not connected by the same letter differ significantly, according to LSMeans differences t test. N.S. denotes not significant (p> 0.05) by t-test. (TIF) [file pgen.1007735.s006.TIF]

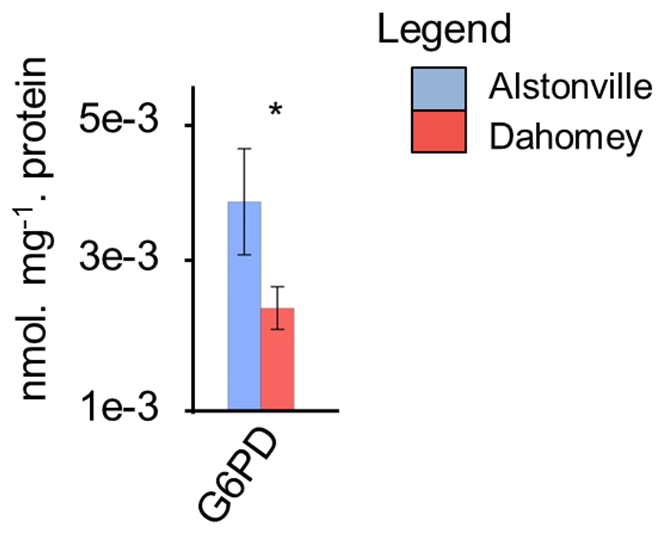

Supplement: S7 Fig — Activity was determined spectrophotometrically from the rate of reduction of NADP (n = 8 biological rep/mitotype). Bars (mean ± s.e.m). * p< 0.05, as calculated by t-tests (see text). (TIF) [file pgen.1007735.s007.TIF]
