# Supplementary material for: Genotype to phenotype: Diet-by-mitochondrial DNA haplotype interactions drive metabolic flexibility and organismal fitness
Source: PLoS Genet. 2018 Nov 6;14(11):e1007735. doi: 10.1371/journal.pgen.1007735 (PMC6219761; doi:10.1371/journal.pgen.1007735)
Supplement: S1 Table — Position is taken from the alignment of GenBank No’s KP843845, KP843849, KP843842 and KP843854, respectively accessed on 12 April 2018. Syn is synonymous, Nonsyn is nonsynonymous, ItSpace is intervening spacer region and Con is consensus. 1 Complex V (M185I). 2 Complex IV (D40N), 3 Complex I (V161L), 4 The G499A (complementary strand). (DOCX) [file pgen.1007735.s008.docx]

| Posn | Gene | Type | Con | Dahomey | Madang | Alstonville | Victoria Falls |
| --- | --- | --- | --- | --- | --- | --- | --- |
| 682 | ND2 | Nonsyn | A | • | • | • | T |
| 1512 | COI | Syn | Y | C | C | T | T |
| 1836 | COI | Syn | R | A | A | G | G |
| 4616 | ATP6 | Nonsyn^1^ | T | • | • | A | • |
| 4853 | COIII | Nonsyn^2^ | G | A | • | • | • |
| 5243 | COIII | Nonsyn | C | • | • | • | A |
| 5963 | ItSpace | Silent | A | • | • | • | T |
| 5967 | ItSpace | Silent | T | • | • | • | A |
| 9056 | ND4 | Nonsyn^3^ | M | A | A | C | C |
| 10673 | Cyt*b* | Syn | C | • | • | T | • |
| 12123 | ND1 | Syn | Y | T | T | C | C |
| 12372 | ND1 | Syn | Y | T | T | C | C |
| 13552 | lrRNA | Silent^4^ | Y | T | T | C | C |
| 14667 | srRNA | Silent | T | • | • | C | • |
| 14829 | srRNA | Silent | T | • | • | A | • |
| 15051 | A+T | Silent | A | • | • | T | • |
| 15113 | A+T | Silent | T | • | • | A | • |
| 15142 | A+T | Silent | W | A | A | T | T |
| 15227 | A+T | Silent | T | • | • | A | • |
| 15247 | A+T | Silent | T | • | • | • | A |
| 15255 | A+T | Silent | T | • | • | • | A |
| 15272 | A+T | Silent | W | A | A | T | T |
| 15392 | A+T | Silent | T | • | • | A | • |
| 15633 | A+T | Silent | C | • | • | T | • |
| 15672 | A+T | Silent | A | • | • | • | G |
| 15883 | A+T | Silent | W | A | T | A | T |
| 15892 | A+T | Silent | A | • | C | • | • |
| 15902 | A+T | Silent | W | A | T | T | A |
| 15904 | A+T | Silent | A | • | • | • | T |
| 15970 | A+T | Silent | W | A | A | T | T |
| 15971 | A+T | Silent | W | T | T | A | A |
| 16004 | A+T | Silent | W | T | T | A | A |
| 16022 | A+T | Silent | A | • | • | • | T |
| 16024 | A+T | Silent | A | • | • | • | T |
| 16065 | A+T | Silent | R | A | A | G | G |
| 16227 | A+T | Silent | A | • | T | • | • |
| 16229 | A+T | Silent | A | • | T | • | • |
| 16250 | A+T | Silent | T | • | C | • | • |
| 16397 | A+T | Silent | T | • | • | C | • |
| 16420 | A+T | Silent | A | • | • | T | • |
| 16429 | A+T | Silent | A | • | • | G | • |
| 16452 | A+T | Silent | A | • | • | T | • |
| 16453 | A+T | Silent | G | • | • | A | • |
| 16568 | A+T | Silent | A | • | • | • | T |
| 16587 | A+T | Silent | A | • | • | T | • |
| 16589 | A+T | Silent | T | • | • | A | • |
| 16798 | A+T | Silent | T | • | A | • | • |
| 16710 | A+T | Silent | T | • | A | • | • |
| 16751 | A+T | Silent | R | A | A | G | G |
| 16933 | A+T | Silent | T | • | • | A | • |
| 16935 | A+T | Silent | A | • | • | T | • |
| 17032 | A+T | Silent | A | • | • | • | G |
| 17051 | A+T | Silent | A | • | T | • | • |
| 17053 | A+T | Silent | T | A | • | • | • |
| 17054 | A+T | Silent | R | A | A | G | G |
| 17091 | A+T | Silent | T | • | • | • | C |
| 17108 | A+T | Silent | T | • | A | • | • |
| 17117 | A+T | Silent | G | • | A | • | • |
| 17121 | A+T | Silent | A | T | • | • | • |
| 17255 | A+T | Silent | G | • | • | A | • |
| 17398 | A+T | Silent | T | • | • | A | • |
| 17400 | A+T | Silent | A | • | • | T | • |
| 17602 | A+T | Silent | T | • | • | • | G |
| 17609 | A+T | Silent |  | • | • | • | G |
| 17690 | A+T | Silent | T | • | • | A | • |
| 17717 | A+T | Silent | T | • | • | A | • |
| 18066 | A+T | Silent | C | • | • | T | • |
| 18069 | A+T | Silent | T | • | G | • | • |
| 18147 | A+T | Silent | T | • | • | A | • |
| 18148 | A+T | Silent | W | T | A | A | T |
| 18155 | A+T | Silent | W | A | A | T | T |
| 18156 | A+T | Silent | W | A | A | T | T |
| 18160 | A+T | Silent | T | • | • | • | A |
| 18162 | A+T | Silent | A | • | • | • | - |
| 18174 | A+T | Silent | -/T | - | - | T | T |
| 18184 | A+T | Silent | A | T | • | - | • |
| 18370 | A+T | Silent | G | A | • | • | • |
| 18536 | A+T | Silent | G | • | • | T | • |
| 18543 | A+T | Silent | A | T | • | • | • |
| 18606 | A+T | Silent | Y | T | C | C | T |
| 18608 | A+T | Silent | W | T | A | A | T |
| 18614 | A+T | Silent | T | A | • | • | • |
| 18615 | A+T | Silent | T | A | • | • | • |
| 18616 | A+T | Silent | A | • | • | T | • |
| 18622 | A+T | Silent | W | A | T | A | T |
| 18835 | A+T | Silent | G | • | • | C | • |
| 19073 | A+T | Silent | T | • | • | - | • |
| 19089 | A+T | Silent | W | A | T | A | T |
| 19095 | A+T | Silent | T | - | A | • | • |
| 19116 | A+T | Silent | T | A | - | • | • |
| 19117 | A+T | Silent | A | • | • | • | - |
| 19232 | A+T | Silent | G | A | • | • | • |
| 19375 | A+T | Silent | T | • | • | A | • |
| 19358 | A+T | Silent | A | • | • | T | • |
