# Supplementary material for: Genotype to phenotype: Diet-by-mitochondrial DNA haplotype interactions drive metabolic flexibility and organismal fitness
Source: PLoS Genet. 2018 Nov 6;14(11):e1007735. doi: 10.1371/journal.pgen.1007735 (PMC6219761; doi:10.1371/journal.pgen.1007735)
Supplement: S3 Table — (A) Alstonville up-regulated/Dahomey down-regulated when larvae are fed the 1:2 P:C food. (B) Alstonville down-regulated/Dahomey up-regulated when larvae are fed the 1:2 P:C food. (C) Dahomey up-regulated/Alstonville down-regulated when larvae are fed the 1:16 P:C food. (D) Dahomey down-regulated/Alstonville up-regulated when larvae are fed the 1:16 P:C food. Columns show the number of genes in the pathway (N), the number that are significantly up (Up) and down (Down) regulated and the P-values corresponding to the up and down counts. (DOCX) [file pgen.1007735.s010.docx]

| **A** |  |  |  |  |  | |
| --- | --- | --- | --- | --- | --- | --- |
| KEGG pathways upregulated in  Alstonville on 1:2 P:C diet | N | Up | Down | P Up | P Down | |
| Protein Processing in endoplasmic reticulum | 124 | 0 | 4 | 1.00 | 0.00486 | |
| **B** |  |  |  |  |  | |
| KEGG pathways upregulated in  Dahomey on 1:2 P:C diet | N | Up | Down | P Up | P Down | |
| Metabolism of xenobiotics by cyt P450 | 57 | 4 | 2 | 2.149e-05 | 0.0431 | |
| Drug metabolism - cyto P450 | 57 | 4 | 2 | 1.49e-05 | 0.0431 | |
| Ascorbate and aldarate metabolism | 26 | 2 | 0 | 2.72e-03 | 1.00 |  |
| Retinol metabolism | 27 | 2 | 0 | 2.93e-03 | 1.00 |  |
| Porphyrin and chlorophyll metabolism | 38 | 2 | 0 | 5.77e-03 | 1.00 |  |
| Pentose and glucuronate interconversions | 40 | 2 | 0 | 6.38e-03 | 1.00 |  |
| Drug metabolism - other enzymes | 43 | 2 | 0 | 7.35e-03 | 1.00 |  |
| **C** |  |  |  |  |  |  |
| KEGG pathways upregulated in  Dahomey on 1:16 P:C diet | N | Up | Down | P Up | P Down |  |
| DNA replication | 35 | 23 | 0 | 1.49e-18 | 1.00 |  |
| Mismatch repair | 20 | 10 | 0 | 4.37e-07 | 1.00 |  |
| Notch signalling pathway | 22 | 10 | 1 | 1.34e-06 | 0.99 |  |
| Nucleotide excision repair | 37 | 12 | 0 | 8.08e-06 | 1.00 |  |
| Base excision repair | 20 | 8 | 0 | 5.07e-05 | 1.00 |  |
| FoxO signalling pathway | 51 | 13 | 9 | 6.09e-05 | 0.65 |  |
| Fanconi anemia pathway | 26 | 9 | 2 | 6.48e-05 | 0.97 |  |
| Insect hormone biosynthesis | 14 | 6 | 0 | 3.01e-04 | 1.00 |  |
| Wnt signalling pathway | 74 | 14 | 4 | 9.28e-04 | 0.99 |  |
| RNA transport | 127 | 20 | 10 | 9.53e-04 | 0.99 |  |
| Other types of O-glycan biosynthesis | 12 | 5 | 0 | 1.17e-03 | 1.00 |  |
| Homologous recombination | 20 | 6 | 1 | 2.65e-03 | 0.99 |  |
| Pyrimidine metabolism | 78 | 13 | 6 | 4.59e-03 | 0.99 |  |
| Hedgehog signalling pathway | 24 | 6 | 1 | 7.15e-03 | 0.99 |  |
| **D** |  |  |  |  |  | |
| KEGG pathways upregulated in  Alstonville on 1:16 P:C diet | N | Up | Down | P Up | P Down | |
| Oxidative phosphorylation | 135 | 0 | 89 | 1.00 | 3.83e-35 | |
| Metabolic pathways | 862 | 47 | 220 | 0.99 | 1.58e-09 | |
| Citrate cycle (TCA cycle) | 41 | 0 | 22 | 1.00 | 5.46e-07 | |
| Endocytosis | 119 | 0 | 44 | 1.00 | 1.70e-06 | |
| Ribosome | 131 | 0 | 46 | 1.00 | 5.14e-06 | |
| Glycerophospholipid metabolism | 59 | 2 | 22 | 0.94 | 6.46e-04 | |
| Carbon metabolism | 108 | 3 | 32 | 0.99 | 4.05e-03 | |
| Phagosome | 71 | 0 | 22 | 1.00 | 9.22e-03 | |
