# Supplementary material for: Genotype to phenotype: Diet-by-mitochondrial DNA haplotype interactions drive metabolic flexibility and organismal fitness
Source: PLoS Genet. 2018 Nov 6;14(11):e1007735. doi: 10.1371/journal.pgen.1007735 (PMC6219761; doi:10.1371/journal.pgen.1007735)
Supplement: S4 Table — (A) Alstonville up-regulated/Dahomey down-regulated when larvae are fed the 1:2 P:C food. (B) Alstonville down-regulated/Dahomey up-regulated when larvae are fed the 1:2 P:C food. (C) Dahomey up-regulated/Alstonville down-regulated when larvae are fed the 1:16 P:C food (D) Dahomey down-regulated/Alstonville up-regulated when larvae are fed the 1:16 P:C food. Columns show the number of genes in the pathway (N), the number that are significantly up (Up) and down (Down) regulated and the P-values corresponding to the up and down counts. (DOCX) [file pgen.1007735.s011.docx]

**A.**

| **Dahomey v Alstonville 1:2 P:C Up** | N | | Up | | | Down | | | P Up | P.Down | | |
| --- | --- | --- | --- | --- | --- | --- | --- | --- | --- | --- | --- | --- |
| *Biological Process*  Regulation of production of miRNAs | 1 | | 1 | | | 0 | | | 0.0034 | 1 | |  |
| Negative regulation of of miRNAs | 1 | | 1 | | | 0 | | | 0.0034 | 1 | |  |
| Regulation of pre-miRNA processing | 1 | | 1 | | | 0 | | | 0.0034 | 1 | |  |
| Negative regulation of pre-miRNA… | 1 | | 1 | | | 0 | | | 0.0034 | 1 | |  |
| Negative regulation of posttranscriptional .. | 2 | | 1 | | | 0 | | | 0.0068 | 1 | |  |
| *Cellular Component* |  |  | |  | | |  | | | |  | |
| Integral component of endoplasmic reticulum | 16 | 1 | | 0 | | | 0.0534 | | | | 1 | |
| Intrinsic component of endoplasmic retic.. | 18 | 1 | | 0 | | | 0.0599 | | | | 1 | |
| Heterotrimeric G-protein complex | 20 | 1 | | 0 | | | 0.0663 | | | | 1 | |
| Extrinsic component of cytoplasmic side … | 22 | 1 | | 0 | | | 0.0727 | | | | 1 | |
| Extracellular matrix | 151 | 2 | | 0 | | | 0.0940 | | | | 1 | |
| *Molecular Function* |  | |  | |  | | |  | | |  | |
| UDP-glycosyltransferase activity | 89 | | 3 | | 0 | | | 0.0034 | | | 1 | |
| L-ascorbate:sodium symporter activity | 1 | | 1 | | 0 | | | 0.0034 | | | 1 | |
| L-ascorbic acid transporter activity | 1 | | 1 | | 0 | | | 0.0034 | | | 1 | |
| Hydroxyisourate hydrolase activity | 1 | | 1 | | 0 | | | 0.0034 | | | 1 | |
| Glucuronosyltransferase activity | 29 | | 2 | | 0 | | | 0.0043 | | | 1 | |

**B.**

| **Dahomey v Alstonville 1:2 P:C Down** | N | Up | | | Down | | P Up | | | | P.Down |
| --- | --- | --- | --- | --- | --- | --- | --- | --- | --- | --- | --- |
| *Biological Process*  Polytene chromosome puffing | 7 | 0 | | | 2 | | 1 | | | | 0.0003 |
| Heat shock-mediated chromosome puffing | 7 | 0 | | | 2 | | 1 | | | | 0.0003 |
| Response to methotrexate | 11 | 0 | | | 2 | | 1 | | | | 0.0008 |
| Response to temperature stimulus | 121 | 1 | | | 4 | | 0.341 | | | | 0.0014 |
| Cellular response to heat | 21 | 0 | | | 2 | | 1 | | | | 0.0032 |
| *Cellular Component* |  | |  | | |  | |  |  | | |
| Synaptic vesicle membrane | 3 | | 0 | | | 1 | | 1 | 0.0122 | | |
| Signal recognition particle receptor … | 4 | | 0 | | | 1 | | 1 | 0.0163 | | |
| Eukaryotic translation elongation factor…. | 5 | | 0 | | | 1 | | 1 | 0.0203 | | |
| ELL-EAF complex | 8 | | 0 | | | 1 | | 1 | 0.0322 | | |
| Peroxisome | 78 | | 0 | | | 2 | | 1 | 0.0403 | | |
| *Molecular Function* |  | | |  | |  | |  | |  | |
| Histidine-tRNA ligase activity | 1 | | | 0 | | 1 | | 1 | | 0.0040 | |
| Pyruvate dehydrogenase kinase activity | 1 | | | 0 | | 1 | | 1 | | 0.0040 | |
| Sulfinoalanine decarboxylase activity | 1 | | | 0 | | 1 | | 1 | | 0.0040 | |
| (S)-2-hydroxy-acid oxidase activity | 1 | | | 0 | | 1 | | 1 | | 0.0040 | |
| Glycolate oxidase activity | 1 | | | 0 | | 1 | | 1 | | 0.0040 | |

**C.**

| **Dahomey v Alstonville 1:16 P:C Up** | N | Up | | | | | Down | | | P Up | | P.Down | | | |
| --- | --- | --- | --- | --- | --- | --- | --- | --- | --- | --- | --- | --- | --- | --- | --- |
| *Biological Process*  Nucleic acid metabolic process | 51  1551 | 290 | | | | | 94 | | | 2.55e-39 | 1 | |  |  |  |
| Mitotic cell cycle | 581 | 155 | | | | | 73 | | | 5.65e-38 | 0.997 | |  |  |  |
| Cell cycle | 827 | 190 | | | | | 109 | | | 6.90e-37 | 0.998 | |  |  |  |
| Cell cycle process | 659 | 158 | | | | | 87 | | | 1.09e-32 | 0.994 | |  |  |  |
| Anatomical structure development | 2877 | 418 | | | | | 522 | | | 3.13e-31 | 0.005 | |  |  |  |
| *Cellular Component* |  | | |  | |  | | |  | |  | | |  |  |
| Nucleus | 1914 | | | 335 | | 133 | | | 4.68e-40 | | 1 | | | |  |
| Chromosomal part | 318 | | | 86 | | 15 | | | 1.63e-21 | | 1 | | | |  |
| Intracellular organelle | 3834 | | | 480 | | 644 | | | 7.94e-21 | | 0.369 | | | |  |
| Chromosome, centromeric region | 67 | | | 36 | | 5 | | | 1.02e-20 | | 0.991 | | | |  |
| Organelle | 3881 | | | 484 | | 648 | | | 1.08e-20 | | 0.450 | | | |  |
| *Molecular Function* |  | |  | |  | | |  | | |  | | | |  |
| DNA binding | 709 | | 186 | | 42 | | | 7.99e-45 | | | 1 | | | |  |
| Nucleic acid binding | 1425 | | 257 | | 109 | | | 1.84e-31 | | | 1 | | | |  |
| Nucleic acid binding transcription factor ... | 421 | | 106 | | 32 | | | 1.16e-23 | | | 1 | | | |  |
| Sequence-specific DNA transcription fac... | 421 | | 106 | | 32 | | | 1.16e-23 | | | 1 | | | |  |
| Organic cyclic compound binding | 2367 | | 337 | | 323 | | | 6.30e-22 | | | 1 | | | |  |

**D.**

| **Dahomey v Alstonville 1:16 P:C Down** | N | | | Up | | | Down | | P Up | | P.Down |
| --- | --- | --- | --- | --- | --- | --- | --- | --- | --- | --- | --- |
| *Biological Process*  ATP metabolic process | 105 | | | 0 | | | 67 | | 1 | | 1.69e-27 |
| Purine nucleoside triphosphate metabolic … | 109 | | | 1 | | | 67 | | 1 | | 4.22e-26 |
| Purine ribonucleoside triphosphate met .. | 109 | | | 1 | | | 67 | | 1 | | 4.22e-26 |
| Nucleoside triphosphate metabolic process | 112 | | | 2 | | | 68 | | 1 | | 5.11e-26 |
| Ribonucleoside triphosphate process | 110 | | | 1 | | | 67 | | 1 | | 9.07e-26 |
| *Cellular Component* |  | |  | | |  | |  | |  | |
| Mitochondrial part | 394 | | 4 | | | 189 | | 1 | | 2.07e-49 | |
| Mitochondrion | 598 | | 17 | | | 246 | | 1 | | 4.25e-49 | |
| Cytoplasmic part | 2041 | | 114 | | | 557 | | 1 | | 6.29e-43 | |
| Cytoplasm | 2659 | | 186 | | | 660 | | 1 | | 4.67e-37 | |
| Mitochondrial membrane part | 139 | | 1 | | | 89 | | 1 | | 2.39e-36 | |
| *Molecular Function* |  |  | | |  | | |  | |  | |
| Hydrogen ion transmembrane transporter .. | 96 | 0 | | | 53 | | | 1 | | 5.76e-18 | |
| NADH dehydrogenase activity | 42 | 0 | | | 31 | | | 1 | | 3.58e-16 | |
| Substrate-specific transmembrane transp… | 577 | 29 | | | 171 | | | 1 | | 8.35e-16 | |
| Substrate-specific transporter activity | 637 | 34 | | | 183 | | | 1 | | 2.16e-15 | |
| Ion transmembrane transporter activity | 503 | 27 | | | 152 | | | 1 | | 6.28e-15 | |
