# Supplementary material for: Genotype to phenotype: Diet-by-mitochondrial DNA haplotype interactions drive metabolic flexibility and organismal fitness
Source: PLoS Genet. 2018 Nov 6;14(11):e1007735. doi: 10.1371/journal.pgen.1007735 (PMC6219761; doi:10.1371/journal.pgen.1007735)
Supplement: S5 Table — Differentially expressed metabolites from whole third instar female wandering larvae as assessed by GC/MS for the (A) 1:2 P:C diet and (B) 1:16 P:C diet. Values are false discovery rates (FDR). (+) Indicates up-regulated in Dahomey while (-) indicates up-regulated in Alstonville. Peak area is relative to Alstonville larvae. Peak area value is mean± s.e.m. (DOCX) [file pgen.1007735.s012.docx]

**A**

| Metabolites  (Whole larvae) | FDR | Peak Area  (Relative) |
| --- | --- | --- |
| Hexose | 0.0007 (+) | 16.98 ± 0.89 |
| Trehalose | 0.0011 (+) | 3.21 ± 2.91 |
| D-Maltose | 0.0019 (+) | 5.00 ± 0.62 |
| Lactate | 0.0019 (+) | 3.67 ± 0.18 |
| Glutaric acid | 0.0037 (+) | 2.58 ± 0.17 |
| L-Maltose | 0.0060 (+) | 4.03 ± 0.37 |
| Alanine | 0.0079 (+) | 1.48 ± 0.10 |
| Xanthine | 0.0079 (+) | 3.29 ± 0.34 |
| L-Mannose | 0.0093 (+) | 2.80 ± 0.47 |
| Arachidic acid | 0.0128 (+) | 2.30 ± 0.34 |
| Galactose | 0.0128 (+) | 5.51 ± 1.13 |
| Stearic acid | 0.0128 (+) | 2.81 ± 0.47 |
| D-Mannose | 0.0135 (+) | 3.22 ± 0.18 |
| Glucose | 0.0148 (+) | 3.05 ± 0.17 |
| Cysteine | 0.0160 (+) | 2.47 ± 0.37 |
| Palmitic acid | 0.0175 (+) | 2.73 ± 0.47 |
| Succinate | 0.0004 (-) | 0.46 ± 0.04 |
| Beta Alanine | 0.0011 (-) | 0.08 ± 0.01 |
| Maleic acid | 0.0123 (-) | 0.47 ± 0.04 |

**B**

| Metabolites  (Whole larvae) | FDR | Peak Area  (Relative) |
| --- | --- | --- |
| Lactate | 0.0001 (+) | 3.12 ± 0.31 |
| Glutaric acid | 0.0009 (+) | 1.98 ± 0.2 |
| D-Mannose | 0.0020 (+) | 2.28 ± 0.24 |
| Stearic acid | 0.0027 (+) | 1.83 ± 0.19 |
| Xylose | 0.0041 (+) | 2.07 ± 0.40 |
| Sorbitol | 0.0043 (+) | 33.13 ± 8.00 |
| Inosine | 0.0084 (+) | 1.76 ± 0.19 |
| Alanine | 0.0090 (+) | 1.26 ± 0.10 |
| Inositol | 0.0159 (+) | 2.77 ± 0.11 |
| Succinate | 0.0234 (+) | 1.11 ± 0.03 |
| Galactofuranoside | 0.0234 (+) | 1.99 ± 0.11 |
| Palmitic acid | 0.0318 (+) | 1.27 ± 0.05 |
| Gluconate | 0.0026 (-) | 0.46 ± 0.08 |
| Aspartic acid | 0.0039 (-) | 0.62 ± 0.04 |
| Xanthine | 0.0334 (-) | 0.54 ± 0.03 |
