# Supplementary material for: Genotype to phenotype: Diet-by-mitochondrial DNA haplotype interactions drive metabolic flexibility and organismal fitness
Source: PLoS Genet. 2018 Nov 6;14(11):e1007735. doi: 10.1371/journal.pgen.1007735 (PMC6219761; doi:10.1371/journal.pgen.1007735)
Supplement: S6 Table — (A) 1:2 P:C diet (B) The 1:16 P:C diet. Values are false discovery rates (FDR) as determined by moderated t-tests for RNA-seq or t-test for RT-qPCR. (+) Indicates up-regulated in Dahomey while (-) indicates up-regulated in Alstonville. MtDNA genes were: rRNA is ribosomal ribonucleic acid, ATP6 is ATPase subunit 6, ND is NADH-ubiquinone oxidoreductase. Nuclear genes were: Bmm is brummer, CrebB is Cyclic-AMP response element binding protein B, eloF is elongase F, ERR is estrogen-related receptor, GlyP is Glycogen phosphorylase, GstE1 is Glutathione S transferase E1, GstE5 is Glutathione S transferase E5, Ilp2 is Insulin-like peptide 2, N is Notch, TFAM is mitochondrial transcription factor A, Zw is Zwischenferment. (DOCX) [file pgen.1007735.s013.docx]

**A**

| Region | Gene | RNA-seq  Control  FDR | RT-qPCR  Control |
| --- | --- | --- | --- |
| mtDNA | srRNA | 0.13 | 0.62 |
|  | 1rRNA | 0.47 | 0.27 |
|  | ATP6 | 0.90 | 0.59 |
|  | ND4 | 0.63 | 0.80 |
|  | ND6 | 0.86 | 0.77 |
| Nuclear | *bmm* | 0.83 | 0.34 |
|  | *eloF* | 0.96 | 0.42 |
|  | *GstE1* | 2.22e^-06^ (+) | 0.001 (+) |
|  | *GstE5* | 2.55e^-05^ (+) | 0.037 (+) |
|  | *TFAM* | 0.88 | 0.84 |

**B**

| Region | Gene | RNA-seq  Control  FDR | qPCR  Control |
| --- | --- | --- | --- |
| mtDNA | srRNA | 0.94 | 0.21 |
|  | lrRNA | 0.34 | 0.81 |
|  | ATP6 | 0.03 (-) | 0.009 (-) |
|  | ND4 | 0.006 (-) | 0.047 (-) |
|  | ND6 | 0.21 | 0.87 |
|  | *bmm* | 0.03 (+) | 0.004 (+) |
|  | *CrebB* | 0.01 (-) | 0.046 (-) |
|  | *eloF* | 0.01 (+) | 0.013 (+) |
|  | *ERR* | 0.16 | 0.628 |
|  | *GlyP* | 0.62 | 0.939 |
|  | *Ilp2* | 0.022 (-) | 0.021 (-) |
|  | *N* | 0.0007 (+) | 0.001 (+) |
|  | *TFAM* | 0.12 (-) | 0.89 (-) |
|  | *Zw* | 0.01 (-) | 0.034 (-) |
