# Supplementary material for: Genotype to phenotype: Diet-by-mitochondrial DNA haplotype interactions drive metabolic flexibility and organismal fitness
Source: PLoS Genet. 2018 Nov 6;14(11):e1007735. doi: 10.1371/journal.pgen.1007735 (PMC6219761; doi:10.1371/journal.pgen.1007735)
Supplement: S7 Table — Value are false discovery rates (FDR) as determined by moderated t-tests for RNA-seq. (+) Indicates up-regulated in Dahomey while (-) indicates up-regulated in Alstonville. (DOCX) [file pgen.1007735.s014.docx]

| Gene | RNA-seq  FDR |
| --- | --- |
| mRpL1 | 0.4563 (-) |
| mRpL11 | 0.6000 (-) |
| mRpL13 | 0.4959 (-) |
| mRpL15 | 0.7058 (-) |
| mRpL17 | 0.4571 (-) |
| mRpL18 | 0.3976 (-) |
| mRpL19 | 0.4891 (-) |
| mRpL2 | 0.3055 (-) |
| mRpL21 | 0.3732 (-) |
| mRpL24 | 0.4973 (-) |
| mRpL27 | 0.5656 (-) |
| mRpL28 | 0.4176 (-) |
| mRpL3 | 0.3512 (-) |
| mRpL35 | 0.4238 (-) |
| mRpL37 | 0.5145 (-) |
| mRpL38 | 0.3193 (-) |
| mRpL39 | 0.4213 (-) |
| mRpL4 | 0.4833 (-) |
| mRpL41 | 0.5110 (-) |
| mRpL43 | 0.4478 (-) |
| mRpL45 | 0.3294 (-) |
| mRpL46 | 0.3738 (-) |
| mRpL49 | 0.5409 (-) |
| mRpL51 | 0.4656 (-) |
| mRpL54 | 0.4419 (-) |
| mRpS11 | 0.4223 (-) |
| mRpS17 | 0.3426 (-) |
| mRpS18A | 0.4105 (-) |
| mRpS2 | 0.3379 (-) |
| mRpS25 | 0.4294 (-) |
| mRpS28 | 0.3781 (-) |
| mRpS29 | 0.3279 (-) |
| mRpS30 | 0.3550 (-) |
| mRpS31 | 0.4487 (-) |
| mRpS33 | 0.6070 (-) |
| mRpS34 | 0.2966 (-) |
| mRpS35 | 0.4937 (-) |
| mRpS5 | 0.5322 (-) |
| mRpS7 | 0.3634 (-) |
| mRpS9 | 0.4676 (-) |
